# A web-based survey on self-management for patients with inflammatory bowel disease in Japan

**DOI:** 10.1371/journal.pone.0287618

**Published:** 2023-07-17

**Authors:** Masakazu Nagahori, Takahito Imai, Mikiko Nakashoji, Ai Tairaka, Jovelle L. Fernandez

**Affiliations:** 1 Department of Gastroenterology and Hepatology, Tokyo Medical and Dental University, Tokyo, Japan; 2 Japan Medical Office, Takeda Pharmaceutical Company Limited, Tokyo, Japan; Changhua Christian Hospital, TAIWAN

## Abstract

**Background/Aims:**

Self-management (SMN) is a recognized component of care for chronic conditions, yet its importance in the context of inflammatory bowel disease (IBD) is unclear. This study evaluates the status of SMN and its relationship with quality of life (QOL) in Japanese patients with IBD.

**Methods:**

A web-based survey was conducted among adult (≥20 years old) Japanese patients with ulcerative colitis (UC) or Crohn’s disease (CD). Registered members of an online IBD information platform completed a 45-item survey covering demographics, diet, treatment, physical condition, stress management, financial concerns, support services, and QOL. SMN was operationally defined by dietary and lifestyle behaviours, and contingency analysis was used to test for associated factors. Individual-level contributions to SMN were identified with logistic regression.

**Results:**

There were 372 responses to the survey (211 with UC, 161 with CD). Approximately 60% of participants practiced SMN and these patients were 4–24% more likely to report positive QOL than those who did not. SMN was more common in patients with CD than those with UC. SMN practice was also associated with IBD-related hospitalisation/surgery and consultation with others about IBD (e.g. physicians, nurses, patients).

**Conclusions:**

The results of this study suggest an association between the practice of SMN and positive QOL in patients with IBD in Japan.

## Introduction

Inflammatory bowel disease (IBD) is characterized by chronic or relapsing inflammation of the gastrointestinal tract and is clinically diagnosed as either ulcerative colitis (UC) or Crohn’s disease (CD). Although IBD is historically more common in Western countries, it has become a global disease [1]. The prevalence of IBD is increasing in Asia [2], possibly because people are consuming more Western diets and more sterile living conditions are impairing natural immunity. In the Japanese population, the number of patients per 100,000 has risen from 18.1 to 172.9 for UC and 5.9 to 55.6 for CD from 1991 to 2014; there are estimated to be around 220,000 patients with UC and 71,000 patients with CD in Japan based on issued medical certificates [2, 3].

IBD tends to develop early in life, with symptoms that include chronic diarrhea, bloody stools, abdominal pain, and an increased risk of cancer [4]. Patients must cope with a prolonged and complicated disease that markedly degrades patient quality of life (QOL), with pervasive symptoms that interfere with work, social relationships, and life activities [5–8]. There are currently no curative therapies for IBD; therefore, treatment goals focus on inducing remission and preventing relapse. However, achieving these goals requires patients to cope independently and without full-time assistance from health care professionals. Such coping behaviours constitute self-management (SMN), a form of care with widely recognized benefits, as exemplified by patients with chronic diseases such as diabetes [9]. SMN is an essential component for treating chronic illness and the concept is currently being developed for patients with IBD [4, 10, 11].

The general definition of SMN is *an individual’s capacity to manage symptoms*, *treatment*, *lifestyle changes*, *and physical/psychological consequences in the context of chronic disease* [12]. SMN falls under the general definition of self-care, which is *an individual’s self-interested participation in life-sustaining and health-promoting activities that meet their functional requirements* [13]. While SMN has been investigated in patients with IBD [14, 15], key knowledge gaps remain. For example, there are no validated test questions on SMN for patients with IBD. In contrast, the validated 5-question Self-Management Self-Test has been developed for patients with depression [16]. In addition, there are few studies of SMN and outcomes for QOL in patients with IBD, particularly in Japan. Here, we developed test questions on SMN for patients with IBD in Japan and conducted a web-based survey to evaluate the status of SMN and its relationship with QOL and factors such as medical treatment. We also placed the analysis within a broader context by asking patients with IBD to list the types of support they need.

## Methods

### Study design and participants

This observational web-based survey of Japanese adult patients with IBD was conducted between February and March of 2020. The survey probed contexts, issues, and needs for SMN in patients diagnosed with either UC or CD who were ≥ 20 years old (S1 Text, section 1.1.2). Patients were stratified into groups based on their survey responses and statistically analysed based on their practice of SMN, diagnosis with UC or CD, and treatment with Bio/JAKi. Further analyses identified factors associated with SMN status using logistic regression.

### Data source and recruitment

The target number of patients was N ≥ 300 (UC: n ≥ 150, CD: n ≥ 150), based on the feasibility of recruiting a sufficient sample from the population within a short time frame whilst meeting the requirements for statistical analysis. Registered members of the IBD Plus panel, owned by QLife Inc. (Tokyo, Japan), were invited to participate in a web-based survey via a direct email sent from QLife in February 2020 (S1 Text, section 1.2). QLife is a commercial medical information company that services consumers, marketers and medical professionals. Patients and their families who are members of the IBD Plus panel have access to information on aspects of the disease, including its pathology and treatment. QLife designed and implemented the survey and provided the raw data and summary tables to Takeda Pharmaceutical Company Ltd (Tokyo, Japan).

QLife sent an invitation email containing documentation to inform members about the study and responders provided their informed consent by following the survey link (S1 Text, section 1.2). The first 3 questions of the survey (and 1 demographic question, see below) were used to screen participants for eligibility (S1 Text, section 1.1; and S2 Text, Screening Questions). Eligible members: i) had been diagnosed with ulcerative colitis or Crohn’s disease at a hospital, ii) had visited a medical institution for treatment within a year, iii) visited a medical institution more than once every 4 months, iv) were 20 years of age or older (S1 Text, section 1.1).

### Survey procedure

Participants first completed a 12-item demographic questionnaire (questions D1–12; S2 Text, Demographic Questions) followed by the main 33-item survey (questions Q1–33; S2 Text, Main Survey). Questions and options were shown together on a screen and presented one at a time in a specified order; respondents were able to review their answers and return to previous questions. There have been no previous validations of SMN-based surveys for patients with IBD, so this survey was based on previous research [11, 17, 18] and the Japanese Self-Care Agency Questionnaire [19]; it covered 7 categories: i) diet (3 items), ii) treatment (9 items), iii) symptoms/physical condition (8 items), iv) stress (3 items), v) financial concerns (4 items), vi) support services and tools (5 items), vii) QOL (1 item).

### Quality control

QLife was responsible for checking the integrity and accuracy of the study data, including identifying and rectifying false settings, errors, and malicious entries. Multiple entries were prohibited with checks on responder IP addresses. Responders with both UC and CD diagnoses (n = 4) were excluded to ensure data independence. Responders who returned an incomplete survey were also excluded.

### Operational definition of SMN

Our analysis requires an operational definition of SMN to classify participants. We assumed that patients practice SMN if they respond positively to the following 3 test questions about their present experiences regarding diet and lifestyle (S2 Text, Main Survey): *(Q2) How often do you check the nutrition labelling when buying food*? (positive: Always, Often; negative: Rarely, Never); *(Q3) Are you able to manage the type and amount of food you eat depending on the situation*? (positive: I’ve been able to manage it well, I’ve been able to manage it to some extent; negative: I haven’t been able to manage it much, I haven’t been able to manage it at all); *(Q18) Are you able to change your diet*, *take leave or rest*, *or visit a doctor depending on your physical condition*? (positive: I have been able to change my behaviour at any time, I have been able to change my behaviour to some degree; negative: I haven’t been able to change my behaviour very much, I have been unable to change my behaviour).

The relationship aspect of SMN was evaluated with 4 questions that asked about consultations with others (Q9, 14, 15, 26; S2 Text, Main Survey). Participants were asked to list all types of people that they consult with (e.g., healthcare professionals, friends and family, other patients, online, nobody), for each of the following topics: i) treatment of IBD; ii) lifestyle (eg, diet, exercise, sleep); iii) physical condition/symptoms; and iv) employment. Responses were analysed qualitatively and quantitatively. Two classification schemes were used for qualitative analysis: i) no consultation versus at least one type of consultation partner (presence/absence) and ii) two or fewer types versus three or more types of consultation partner (limited/diverse). The two different schemes were used because we assumed that consultation patterns would be concentrated toward doctors and family members; hence, those with three or more types of partners are likely to represent patients with greater potential to discuss issues surrounding IBD. The quantitative analysis used the absolute number of partners reported.

### Variables and outcomes

Analytical variables were determined from responses to single survey items, with positive-negative categorical definitions based on scale thresholds as stated here or in S2 Text. Continuous responses were analysed numerically. The effectiveness of SMN was evaluated as QOL with *(Q33) How do you perceive your daily life with UC or CD*? (positive: I can live very well with UC/CD, I can live well with UC/CD; negative: I cannot live comfortably with UC/CD, I cannot live with UC/CD). Predictor variables used to characterize patients covered demographics, disease status, diet, treatment, physical condition/symptoms and stress management (see S2 Text for all 45 survey items). Importantly, patients that listed biologics or Janus kinase inhibitors in their prescribed medications (Item D5) were categorized into the Bio/JAKi sub-group, as coded by the authors (see S1 Text, section 2.1).

The types of support that patients require were assessed with a direct question to mark all relevant sources from: financial and employment, symptom control (eg, food, body), mental support, other (see S1 Text, section 2.2). Participants could also provide a free description for their response, which was categorized by authors.

### Analysis procedures

Patients were categorized by diagnosis with UC or CD, the operational definition of dietary and lifestyle SMN, and presence or absence of Bio/JAKi treatment. The associations between these classifications and other survey responses were explored with contingency tables using chi-squared and Fisher’s exact tests (except for age and IBD duration, which were analysed with two-sample t-tests). Logistic regression models were used to better identify factors associated with the categorization of SMN for all patients with IBD, patients with UC, and patients with CD. The predictive factors for each model were chosen by first performing separate logistic regressions for each item from the full response set (excluding Q30 [*What kind of support do you want to get for UC or CD*?] because it has no direct relationship with SMN) and selecting factors with P < 0.050. Selected predictors were checked for multicollinearity using their variance inflation factors (VIF < 10), and a full model containing all selected factors was reduced by forward-backward-stepwise selection to produce the inference model. The statistical analysis was conducted using SAS (version 9.3, copyright 2011 SAS Institute Inc., Cary NC, USA). Differences were considered statistically significant at P < 0.05 (* P < 0.050, ** P < 0.010, *** P < 0.001).

### Ethical considerations

Subject screening and survey content were approved by the Ethics Committee of Tokyo Medical and Dental University (M2019-274) and they conform to the provisions of the Declaration of Helsinki. Participants provided their written informed consent by responding to the invitation email and following the link to the survey (see S1 Text, section 1.2 for subject briefing and consenting information). Participant data is maintained and stored by QLife Inc. (Tokyo, Japan) and high-level authorities at QLife maintain the means to connect survey responses with personal information. However, records for consenting participants were provided to Takeda and the authors by QLife in an anonymized format. The study was made following STROBE [20] and CHERRIES [21] guidelines. More details about ethical concerns and data management are in S1 Text, sections 1.2, 1.3.

## Results

### Patient characteristics

In total, 372 respondents met the criteria and were evaluated, including 211 patients with UC and 161 with CD (Table 1; S1 Text, section 1.1). Participants had a mean age of 42±12 years old, and 10±10 years of disease duration on average. Male participants represented 41% of patients with UC and 57% of those with CD.

**Table 1:** Patient characteristics for all patients and by diagnosis.

Characteristic, n (%)	All (n = 372)	UC (n = 211)	CD (n = 161)	P value
Age (years), mean ± SD	42.0±11.8	43.9±11.9	39.5±11.1	<0.001
Sex				0.005
Male	178 (47.9)	87 (41.2)	91 (56.5)	
Female	194 (52.2)	124 (58.8)	70 (43.5)	
IBD duration (years), mean ± SD	10.3±9.9	8.7±8.9	12.3±10.8	0.001
IBD severity				0.061
Remission	116 (31.2)	59 (28.0)	57 (35.4)	
Mild	104 (28.0)	64 (30.3)	40 (24.8)	
Moderate	119 (32.0)	75 (35.6)	44 (27.3)	
Severe	13 (3.5)	6 (2.8)	7 (4.4)	
Unknown	20 (5.4)	7 (3.3)	13 (8.1)	
Medical institution visited				0.019
University hospital	128 (34.4)	61 (28.9)	67 (41.6)	
General hospital	174 (46.8)	104 (49.3)	70 (43.5)	
Clinic	69 (18.6)	46 (21.8)	23 (14.3)	
Others	1 (0.3)	0 (0.00)	1 (0.6)	
Treatment				
Biologics ^a^	131 (35.2)	46 (21.8)	85 (52.8)	<0.001
JAKi	10 (2.7)	10 (4.7)	0 (0.00)	
Bio/JAKi ^a^	141 (37.9)	56 (26.5)	85 (52.8)	<0.001
5-ASA	309 (83.1)	183 (86.7)	126 (78.3)	
Steroids	65 (17.5)	54 (25.6)	11 (6.8)	
Immunomodulators	92 (24.7)	36 (17.1)	56 (34.8)	
Immunosuppressants	5 (1.3)	3 (1.4)	2 (1.2)	
Others ^b^	53 (14.3)	7 (3.3)	46 (28.6)	
Bowel movements during the past week				0.481
0–2/day	185 (49.7)	112 (53.1)	73 (45.3)	
3–4/day	108 (29.0)	58 (27.5)	50 (31.1)	
5–9/day	65 (17.5)	33 (15.6)	32 (19.9)	
≥ 10/day	14 (3.8)	8 (3.8)	6 (3.7)	
Relapses in the previous year				0.001
0	149 (40.1)	67 (31.8)	82 (50.9)	
1–2	146 (39.3)	98 (46.5)	48 (29.8)	
≥3	32 (8.6)	21 (10.0)	11 (6.8)	
Always/chronic ≥6mo	45 (12.1)	25 (11.9)	20 (12.4)	
History of hospitalization				<0.001
No	108 (29.0)	89 (42.2)	19 (11.8)	
Yes	264 (71.0)	122 (57.8)	142 (88.2)	
History of surgery				<0.001
No	279 (75.0)	201 (95.3)	78 (48.5)	
Yes	93 (25.0)	10 (4.7)	83 (51.6)	
Employed ^c^				0.716
No	93 (25.0)	50 (23.7)	43 (26.7)	
Yes	252 (67.7)	141 (66.8)	111 (68.9)	
Other	27 (7.3)	20 (9.5)	7 (4.4)	
Annual income				0.223
< 4 million yen	248 (66.7)	135 (64.0)	113 (70.2)	
≥ 4 million yen	124 (33.3)	76 (36.0)	48 (29.8)	

Ulcerative colitis (UC) and Crohn’s disease (CD) were compared using Fisher’s tests for categorical variables and t-tests for continuous variables (age, IBD duration).

^a^ Test performed for binary value of ‘applicable’ or ‘not applicable’ for the corresponding treatment.

^b^ Includes Ciprofloxacin, Metronidazole, Elemental diet, Hiroshima Kampo, Indigo naturalis.

^c^ Test compared ‘No’ and ‘Yes’, but not ‘Other.’

5-ASA, mesalamine; CD, Crohn’s disease; IBD, inflammatory bowel disease; JAKi, Janus kinase inhibitors; SD, standard deviation; UC, ulcerative colitis.

### SMN practice and QOL

**Fig 1:**
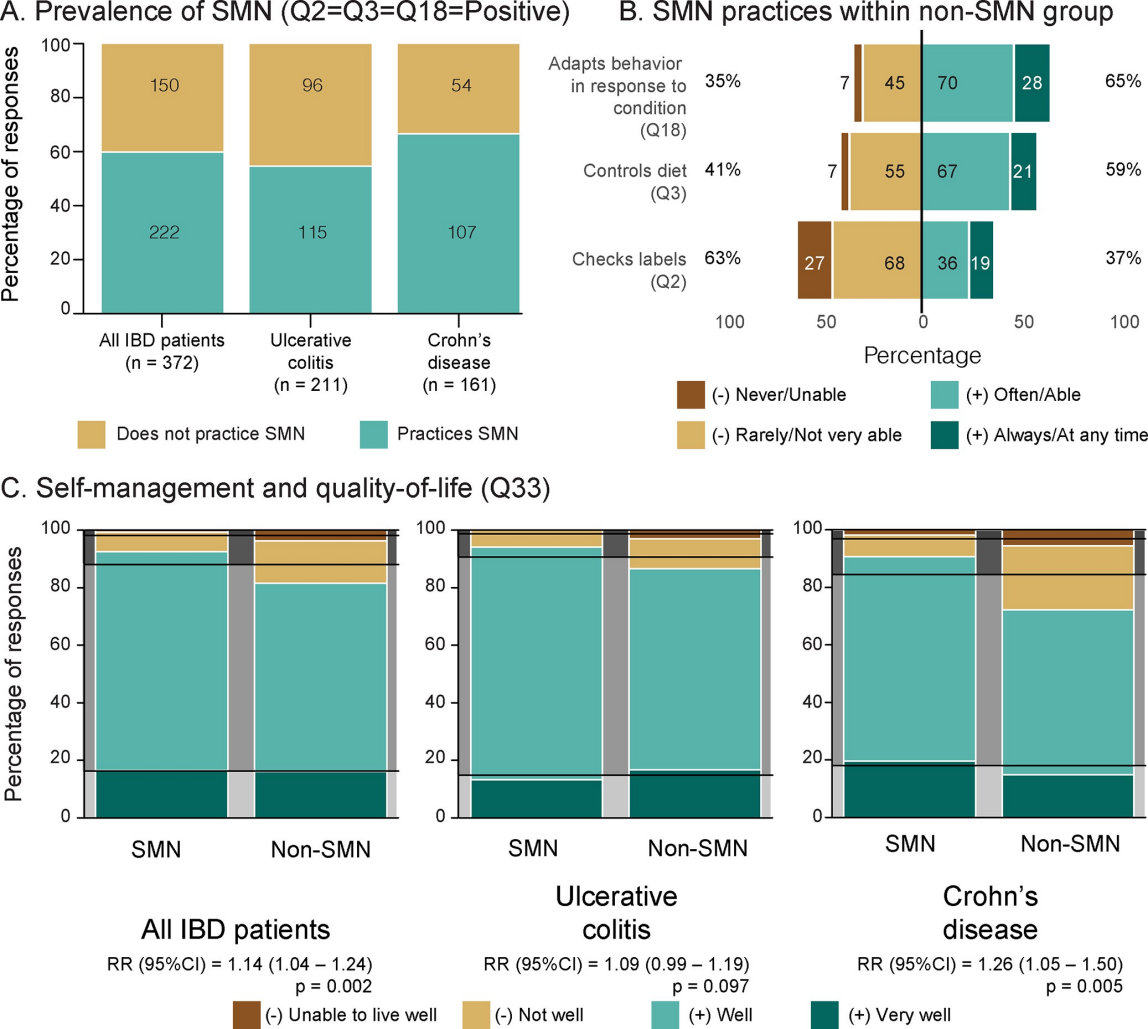
SMN of IBD. (A) Proportion of patients who practice SMN according to our criteria. (B) Prevalence of component SMN practices within the non-SMN group among all patients with IBD. (C) Associations between SMN and quality of life according to Fisher’s exact test and risk ratios, with expected proportions marked by black lines. For complete wording of the questions, see S2 Text; Main Survey). CI: confidence interval. IBD: inflammatory bowel disease. OR: odds ratio. RR: risk ratio. SMN: self-management.

The majority of patients with IBD practiced SMN (59.7%) according to our operational definition and the practice was more common among patients with CD (66.5%) than those with UC (54.5%; Fig 1A). However, even patients that did not fully practice SMN according to our criteria still performed some of the component practices (Fig 1B). For example, the majority of non-SMN patients with IBD controlled their diets (Q3) and adapted their behaviour in response to their conditions (Q18); however, relatively few checked dietary labels (Q2).

The effectiveness of SMN was determined by asking participants to report their QOL with regards to living with UC or CD. Here, most patients responded that they can live well with IBD (Fig 1C). However, QOL was significantly associated with patients that practiced SMN. Specifically, patients with IBD that practiced SMN were 4% to 24% more likely to report a positive QOL than those who did not. This result was mainly associated with patients diagnosed with CD, who were significantly around 5% to 50% more likely to report better QOL if they practiced SMN, whereas the outcome for patients with UC was only borderline significant (Fig 1C; S1 Text, section 2.2).

### Differences between SMN and non-SMN groups

**Fig 2:**
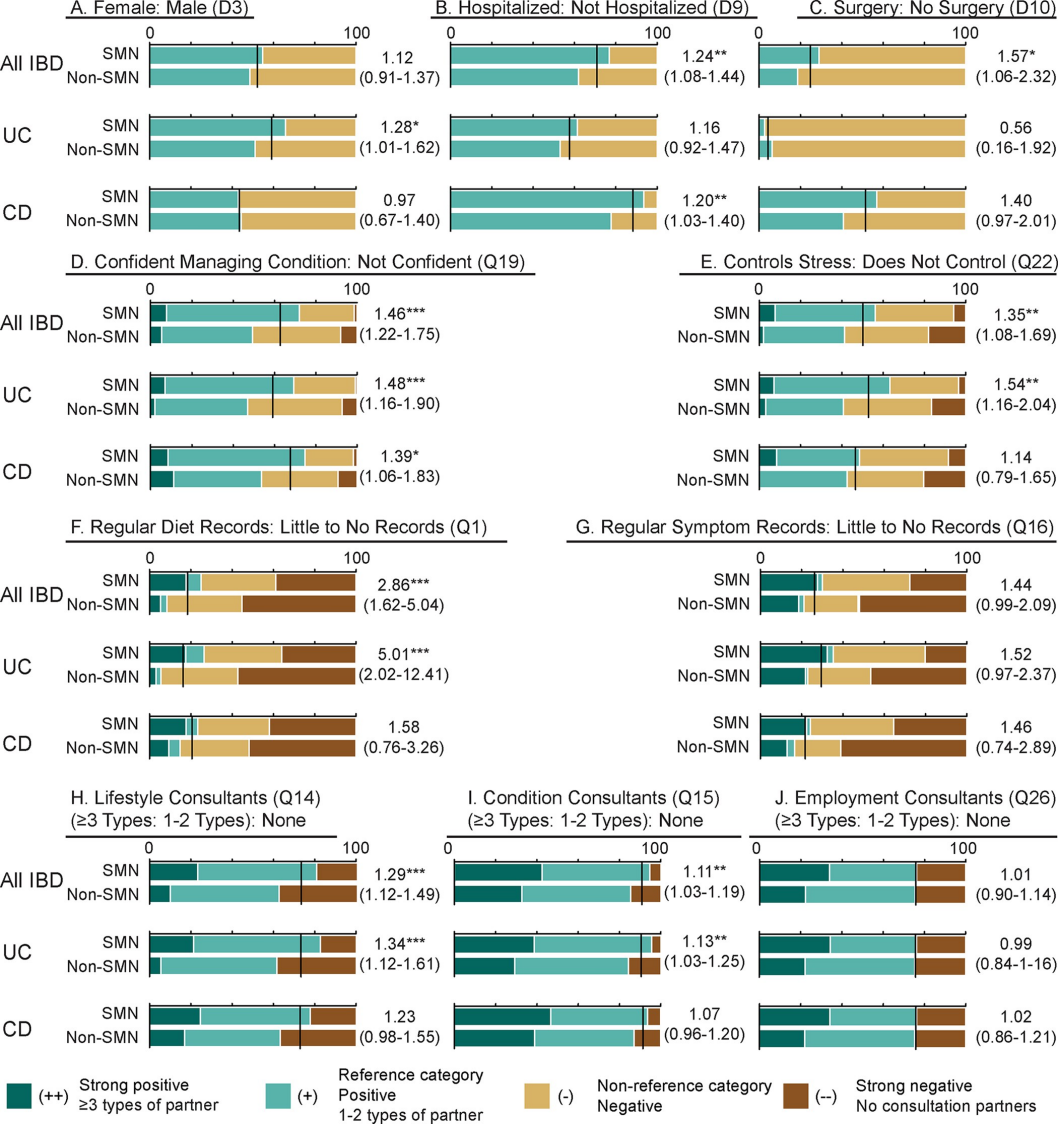
Results for Fisher’s exact tests of association between SMN and selected responses. See S1 Text (section 2.2) for full result set and S2 Text for complete question wording, threshold criteria and responses. Black lines show the expected proportions between categories, independent of SMN classification. Results for each test shown as risk ratios (95% CI), with P-value: * P ≤ 0.050; ** P ≤ 0.010, *** P ≤ 0.001. Note that graphical grading for ‘Consultants’ are relevant to classification schemes i (presence/absence: green vs brown) and ii (≥3/<3: dark green vs remainder), but the statistical results refer only to scheme i. CI: confidence interval. IBD: inflammatory bowel disease. RR: risk ratio. SMN: self-management.

Notable comparisons are shown in Fig 2 and full results are provided in S1 Text, section 2.2. There were no associations between SMN and patient sex among all patients with IBD, although patients with UC who practiced SMN were disproportionately female (Fig 2A). SMN users were 8% to 44% more likely to have had a history of hospitalization for IBD compared with non-SMN users, with this trend being most pronounced in patients with CD (Fig 2B). Similarly, SMN users were significantly more likely to have undergone resection surgery than non-SMN users, although this association was absent for patients with UC and borderline for patients with CD when considered independently (Fig 2C).

Patients in the SMN group were significantly more likely to report confidence in managing their physical condition (Fig 2D) and a sense of control when managing IBD-related stress (Fig 2E). This association was strongest for patients with UC. By contrast, patients with CD that use SMN tended to be confident living with their condition but there was no difference in their reported stress management (Fig 2D and 2E). Similarly, keeping frequent records of diet was a practice significantly associated with SMN for patients with UC but not for patients with CD (Fig 2F). Indeed, patients with UC who practice SMN were 5 times as likely to keep frequent diet records (several times a month or more) as patients with UC who do not practice SMN. Conversely, keeping symptom records was not significantly associated with practicing SMN, although the association approached significance for patients with UC and all patients with IBD together (Fig 2G).

There were significant associations between the operational definition of SMN (i.e. awareness, planning, decision making, and action components) and consultation with others about IBD (i.e. the relational component), as patients that use SMN were more likely to have at least one type of person to consult with about IBD than those that do not practice SMN (Fig 2H–2J). Similarly, SMN users tended to have a greater likelihood of consulting with diverse partner types (≥3) than non-users. This general tendency was strongest for patients with UC with regards to consultation about lifestyle, with SMN users having a 12% to 61% greater likelihood of consulting (Fig 2H). Furthermore, patients with UC that practiced SMN were approximately 4 times as likely to consult about lifestyle with 3 or more types of people than non-SMN users (RR = 4.01, 95% CI: 1.59–10.10, P = 0.001; S1 Text, section 2.2). SMN was also associated with a small but significant likelihood to consult about physical symptoms/conditions, and again this pattern was more apparent among patients with UC than those with CD (Fig 2I). Considering employment and treatment-related concerns, the associative trend between SMN and consultation was not significant for the presence/absence criterion (Fig 2J) but SMN users were more likely to have a diversity of (≥3 types) of consultation partners for employment (RR = 1.56, 95CI: 1.09–2.21, P = 0.015) and treatment concerns (RR = 1.44, 95% CI: 1.09–1.90, P = 0.009) than non-SMN users (S1 Text, section 2.2).

### Predicting the use of SMN

**Fig 3:**
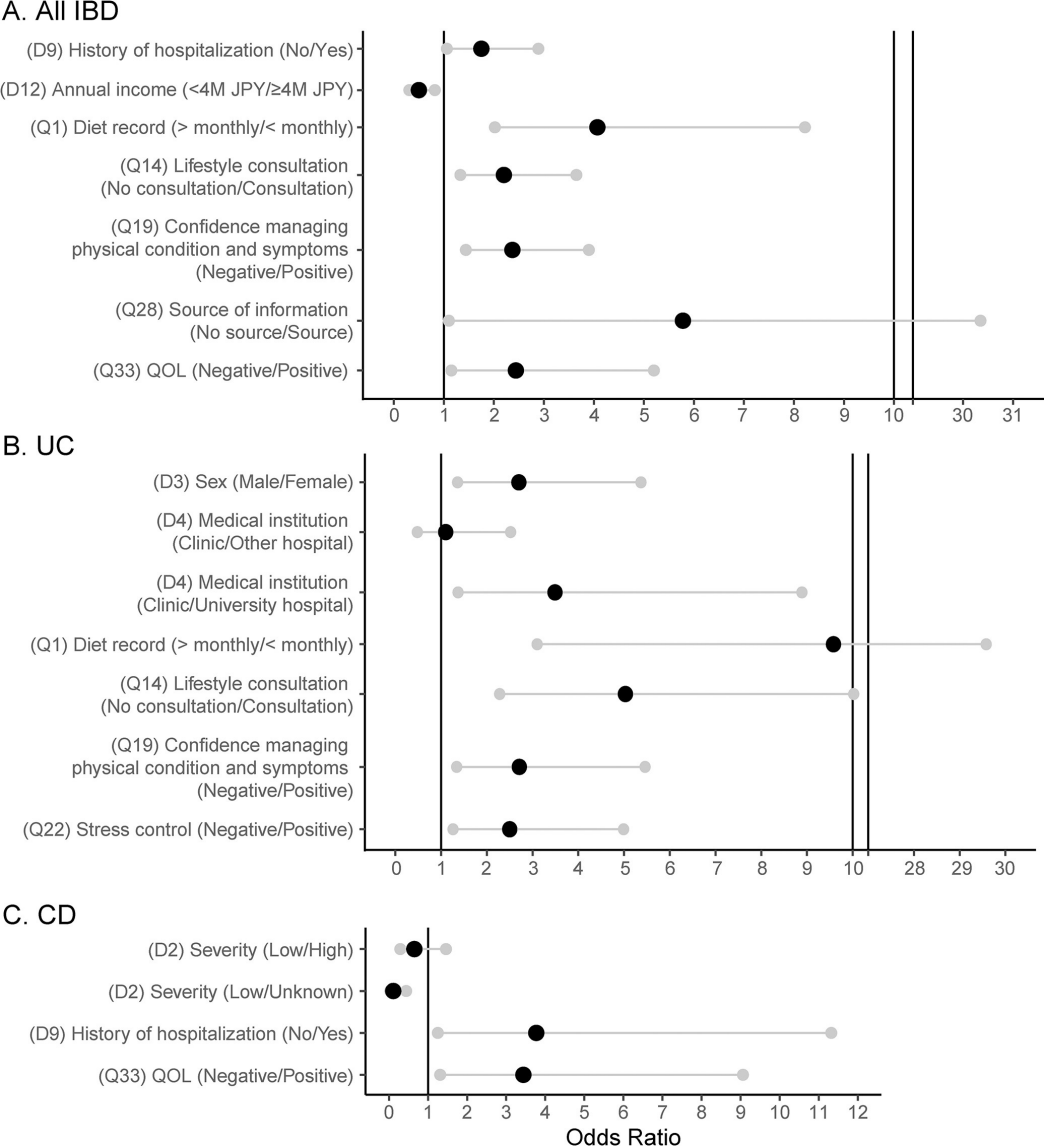
Odds ratios and 95% confidence intervals for selected logistic regression models testing for associations with SMN. (A) All patients with IBD. (B) Patients with UC. (C) Patients with CD. Cuts in the axis, due to wide intervals for (A) and (B), are represented by vertical lines. For complete wording of the questions, see S2 Text. Parentheticals following the factor represent reference level/tested factor, with thresholds presented in S2 Text. CI: confidence interval. IBD, inflammatory bowel disease. MJPY: millions of Japanese Yen. QOL: quality of life. SMN: self-management.

Logistic regression was used to identify factors that correspond with individual likelihoods to practice SMN (Fig 3). Among all patients with IBD, SMN was significantly more likely to be practiced by those who had a history of hospitalization (χ^2^ = 4.87, P = 0.027). Furthermore, SMN was significantly more likely to be practiced in patients who consulted others about lifestyle (χ^2^ = 9.41, P = 0.002) and had a source for information about IBD (χ^2^ = 4.29, P = 0.038). The practice of keeping diet records was also significantly associated with SMN (χ^2^ = 15.33, P < 0.001). Patients who had confidence managing their physical condition (χ^2^ = 11.52, P = 0.001) and who reported a positive QOL (χ^2^ = 5.34, P = 0.021) were also significantly more likely to practice SMN. Finally, SMN was practiced more by patients with lower income (χ^2^ = 7.65, P = 0.006).

SMN practice among patients with UC was significantly associated with females (χ^2^ = 8.02, P = 0.005) and visitations to university hospitals (χ^2^ = 9.70, P = 0.008). Keeping diet records (χ^2^ = 15.42, P < 0.001) and consulting others about lifestyle (χ^2^ = 15.95, P < 0.001) were also predictive of SMN, as were confidence in managing physical conditions (χ^2^ = 7.72, P = 0.005) and stress (χ^2^ = 6.83, P = 0.009). Among patients with CD, patients who practiced SMN were likely to have had a history of hospitalization (χ^2^ = 5.58, P = 0.018) and were also more likely to report positive QOL (χ^2^ = 6.26, P = 0.012) and less severe symptoms (χ^2^ = 9.67, P = 0.008).

## Discussion

In general, there is no consensus on the definition of SMN or its outcome measures in the field of IBD. This stands in contrast to definitions of SMN in chronic conditions such as diabetes, for which there are clearly defined monitoring and treatment procedures. At the current level of understanding for IBD, SMN is a more nebulous concept closer to intractable conditions such as depression and other neurological disorders. Therefore, developing the concept of SMN for patients with IBD necessitates the use of arbitrary survey constructs to understand how patients take care of themselves and the outcomes this has for their lives. This is the first study to evaluate SMN practices in the context of adult Japanese patients with IBD. We operationally defined SMN based on dietary and lifestyle behaviours and found that SMN was practiced by most respondents and was significantly associated with better QOL. However, there were significant differences between patients with UC and CD that may correspond with differences in their patient journeys and contact with healthcare professionals who may help to promote SMN. Further, associations between SMN and hospitalization, surgery and treatment with Bio/JAKi suggest that key events or increased contact with healthcare professionals may facilitate the practice of SMN.

The findings of the present analysis are limited by the unvalidated 3 test questions on ‘dietary and lifestyle SMN’ in patients with IBD. These questions put less emphasis on other aspects of SMN, such as therapeutic treatment of stress and physical condition. Nevertheless, we consider the associations measured in the present analysis to reflect the broader concept of SMN and its outcomes. Specifically, patients that answered positively to the 3 classifying questions were also significantly more likely to practice record-keeping and have a diversity of consultation partners. Together, these behaviours better reflect the broader concept of SMN of an intractable condition, which involves awareness, planning, decision making, action and relationships [16]. Furthermore, the associations between dietary and lifestyle SMN were significantly associated with confidence in managing symptoms and stress, speaking to broader concepts of QOL outcomes. Therefore, the results of this study will help form the basis for more refined and validated tools to classify SMN and QOL in the context of IBD.

### SMN of IBD in Japanese adult patients

The age and disease profiles for participants in this survey reflect the population of Japanese adult patients with IBD. However, the proportions of patients with UC and CD in this sample (57% and 43%, respectively) markedly differed from the proportions in a 2016 survey of 1035 people (77% and 23%, respectively) [22] and estimates of the Japanese population (76% and 24%, respectively) [3].

Despite its voluntary nature, approximately 60% of surveyed patients practiced dietary and lifestyle SMN according to our operational definition. Furthermore, doctors and families frequently discussed lifestyle, symptoms, and treatment with patients. These results suggest that adult patients with IBD in Japan engage with SMN to some extent. However, this interpretation has some limitations. First, all participants were members of the IBD Plus panel, which is a specialized information and service provider for patients with IBD. Therefore, it is likely that the sampled participants are more informed and conscious about IBD and have more positive attitudes toward treatment than the general population of patients with IBD. Second, respondents had lived with IBD for 10 years on average and may thus have become proficient in managing their condition. However, there is little evidence to suggest that implementation of SMN behaviour increases with disease duration. One study shows that young patients with IBD (10 to 21 years old) tend to become more proficient with SMN as they age, but this was independent of disease duration [23]. Third, approximately 60% of the sample reported mild severity or remission concerning their current status (Table 1), and IBD SMN tends to be easier when symptoms are calm [24]. Finally, the survey was only available online and thereby excludes those without internet access.

The practice of dietary and lifestyle SMN, as well as consultation with nurses and other patients with IBD, was slightly more common amongst patients with CD than those with UC. Further, the multivariate analysis suggested that factors contributing to SMN practice differed between patients with CD and UC. For example, history of hospitalization was associated with SMN in patients with CD but not in those with UC. These results may reflect differences in background characteristics, disease courses, and patient journeys, and a consequent need to consider SMN by diagnosis [25, 26]. Specifically, patients with CD tend to have longer histories of involvement with healthcare professionals and more frequent visits to medical institutions than patients with UC, who tend to receive treatment in an outpatient setting. Patients with CD are often also provided directions by healthcare providers about the impact of nutrition on their symptoms [27]. Such differences may be reflected in our study, as patients with CD had an average of almost 4 more years since disease onset and were more likely to have been hospitalized or received surgery. Thus, patients with CD in our study may have had more opportunities to communicate with healthcare providers and other patients to learn about their disease, whilst opportunities may have been more limited for patients with UC. In addition, the events of hospitalization and surgery may have been severe enough to motivate patients toward a more active role in their treatment [22]. Overall, contact with healthcare providers and the education they provide may have a meaningful influence on all aspects of SMN, including awareness, planning, decision making, action and relationships. This suggests that dedicated support systems for patients with IBD may also help to improve their QOL.

Interestingly, keeping regular diet records was a significant predictive factor for dietary and lifestyle SMN among patients with UC but not patients with CD. This result arises despite there being less evidence supporting the role of diet in treating UC in comparison with CD [28] and the respectively lower degree of intervention from healthcare providers regarding diet control [27]. The reasons for this are unknown; however, it has been reported that many patients with UC have a belief that diet impacts disease activity [29]. Patients with UC who are able to keep regular diet records, despite a lack of evidence or intervention from healthcare providers, may therefore have a higher capacity for SMN than those who do not keep records.

### Associations between SMN and quality of life

This study finds clear evidence to support an association between dietary and lifestyle SMN and QOL, including greater confidence managing symptoms and stress. As mentioned previously, the reports for satisfactory QOL may be related to the sample under investigation because participants mostly reported low disease severity. Furthermore, the beliefs patients have about IBD are tied with their coping ability [30], and members of the IBD Plus panel may have greater awareness and understanding of their condition than the general population of patients with IBD.

Notably, very few participants kept records of their diet or symptoms. Even in the SMN group, ~25% of patients kept regular records. This lack of record keeping is important as self-monitoring can be a powerful component of self-care and SMN when understood correctly. Tracking behaviours allows for the identification of problematic foods and provides helpful records when health is deteriorating. Moreover, the essence of record-keeping lies in helping the patient understand their own patterns and habits [31]. Conceptually, self-monitoring comprises the interaction of self-awareness (of the body, sensations, cognitive processes, and habits) and information gathering (through observations and measurements) to guide independent action or consultation with other care providers [32]. The interactive dynamic between awareness and information gathering can thereby improve SMN and consequently QOL through enhanced recognition of symptoms, more efficient disease regulation, and more effective goal-setting [32]. There have been recent developments in self-monitoring tools and programs for patients with IBD [33–35]; however, there are relatively few studies of self-monitoring in the context of IBD compared with other chronic diseases such as diabetes and hypertension. Gathering evidence for the effectiveness of self-monitoring tools is thus an important avenue for future research in IBD. In this respect, our results suggest that the practice may be infrequent among patients with IBD. This may be because tracking behaviours and physical condition requires dedication and practice. In addition, the seemingly uncontrollable nature of IBD may reduce the motivation of patients to practice SMN. In this regard, self-monitoring practices tend to be common in patients who have diseases with controllable symptoms such as diabetes, asthma, or hypertension, and rare in those with uncontrollable diseases like rheumatism, migraine, or neurological disorders [36].

## Conclusion

This survey provides the first insights into the practice of SMN in adult patients with IBD in Japan. The findings suggest that dietary and lifestyle SMN contributed to improved QOL and was associated with a history of hospitalization and/or surgery, consultation with others, and keeping dietary records. SMN users also tended to have more confidence managing their physical condition and symptoms. Promoting education on SMN for patients with chronic IBD in Japan may therefore help to improve their QOL. The study was limited by our operational definition of SMN, which was an arbitrary and unvalidated construct comprising 3 questions relating to diet and lifestyle. However, the research objectives at this stage are to move toward more robust measurements and this analysis will help to develop the concept of SMN into a treatment framework for IBD. This includes the validation of important indices such as nutritional awareness, diet control, behavioural change, record-keeping and consultation with others. Such developments are necessary to properly treat this chronic illness by supplementing medical care with self-care.

## Supporting information

S1 Text Supplementary methods and results. Includes sections on 1) participant selection; 2) initial contact, consent and incentives; 3) data protection; 4) tables of desired support systems (financial/employment, symptom management, mental health); 5) full set of contingency tables and statistical tests.
(DOCX)

S2 Text Supplementary survey and responses. Full English version of the survey, showing frequency distribution of responses for each item, stratified by diagnosis.
(DOCX)
