# Supplementary material for: A web-based survey on self-management for patients with inflammatory bowel disease in Japan
Source: PLoS One. 2023 Jul 17;18(7):e0287618. doi: 10.1371/journal.pone.0287618 (PMC10351702; doi:10.1371/journal.pone.0287618)
Supplement: S1 Text — Includes sections on 1) participant selection; 2) initial contact, consent and incentives; 3) data protection; 4) tables of desired support systems (financial/employment, symptom management, mental health); 5) full set of contingency tables and statistical tests. (DOCX) [file pone.0287618.s001.docx]

A web-based survey on self-management for patients with inflammatory bowel disease in Japan

*Masakazu Nagahori, Takahito Imai, Mikiko Nakashoji, Ai Tairaka, Jovelle L Fernandez*

1. Supplementary Methods

# Participant selection

## Inclusion criteria

The participant meets *all* of the following conditions:

- Registered in IBD Plus Panel
- Diagnosed with ulcerative colitis or Crohn’s disease
- Provided informed consent
- Visited a medical institution for treatment of IBD within past year

## Exclusion criteria

The participant meets *any* of the following conditions:

- Under 20 years old
- Did not provide informed consent
- Visits a medical institution fewer than once every four months
- Returned incomplete or non-applicable survey


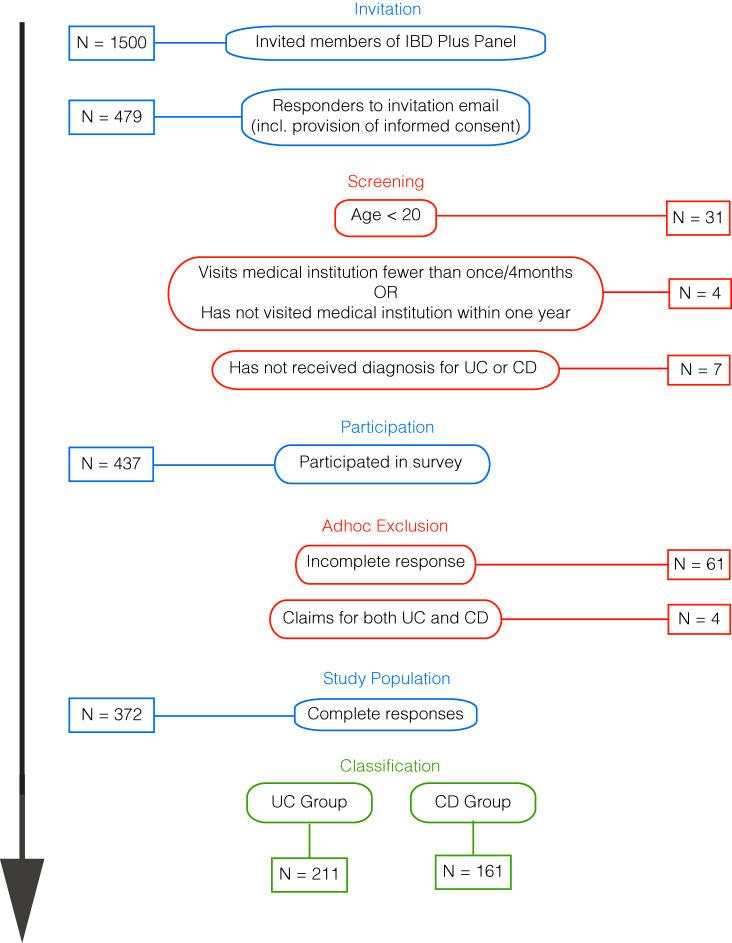


**Fig. S1.** **Identification of study population, showing criteria for inclusion, exclusion, and classification.**
*CD: Crohn’s disease. IBD: inflammatory bowel disease. UC: ulcerative colitis.*

# Initial contact, consent and incentives

The following is an English translation of the initial contact email sent to eligible members of the IBD Plus Panel. Members provided their informed consent in their reply to the email. Participants who provided a complete response and an e-mail address were given an electronic Amazon gift card worth 1000 Japanese Yen (~$9 USD circa 2020) by QLife.

We are conducting a survey targeting patients with inflammatory bowel disease (ulcerative colitis and Crohn's disease).

The objective of this survey is to clarify the implementation status, issues, and needs related to self-management in patients with inflammatory bowel disease in order to investigate the ideal way to self-manage inflammatory bowel disease in the future.

The survey period will be from early February 2020 to late February 2020. However, the survey may end without notice as soon as 300 responses are received.

This questionnaire is supervised by Dr. Masakazu Nagahori, Associate Professor of Gastroenterology at Tokyo Medical and Dental University. The survey is outsourced by Takeda Pharmaceutical Company Limited, and operated by QLife Inc. (hereafter referred to as “our company”), and is reviewed by an Ethics Committee.

Please read below about the handling of personal information and responses to the questionnaire.

1. **Person in charge of personal information**

Personal information collected in this questionnaire will be handled only by our company.

1. **Purpose of use of personal information**

To be used only for communication and sending a gift voucher.

1. **Personal information handling**

Our company shall, if all or part of the handling of personal information is outsourced, establish a management system that can appropriately protect personal information.

Personal information will be strictly managed by including a confidentiality agreement with any outsourcees after carefully selecting the outsourcee.

1. **Contact for inquiries on personal information**

Our company has appointed a personal information protection manager (affiliated with the Media Planning Office). Personal information is handled appropriately and safely. Please refer <here> for public information and contact information regarding handling of personal information.

1. **Detailed report on questionnaire response**

The responses maybe statistically analysed and used/re-used for academic purposes, market research, etc.

Even if the results are not presented in a scientific article or at a scientific meeting, the results will not contain any information that can identify individuals.

1. **Other**

For other usage of the services operated by our company, please refer to our service conditions of use <here>.

**Reduction of burden**

In order to reduce the burden associated with your participation in this survey, we will send an Amazon gift card worth 1000 Yen to those who have cooperated with us. (If you would like to receive an Amazon gift card please provide an e-mail address. If you do not require the gift card there is no need for you to provide your email)

**Participation in the survey**

Your participation in this study is voluntary. By ticking “*I agree with the handling of personal information and questionnaire responses*” at the end of this explanation screen and pressing the “*Proceed to survey*” button, you agree to participate in this survey. After answering all the questions, you can send your answers to our company system by pressing the “*Send Answers*” button.

If you wish to discontinue the use of your personal information after completing the questionnaire, please contact us.

**Handling of safety information**

QLife Inc. has been requested by Takeda Pharmaceutical Company Limited to report any adverse events (e.g., adverse reactions) related to Takeda products that are identified from your responses to this questionnaire. In the case that the adverse event is serious, QLife Inc. will ask you the name of your doctor and the institution to which they belong, and whether it is acceptable to inform Takeda Pharmaceutical Company Ltd.

# 1.3. Data protection

Takeda Pharmaceutical Company Ltd. will store data on a secure server and within a locked cabinet for a period of 3 years. After this time, material will be disposed of such that it cannot be restored: paper media will be shredded and electronic files will be deleted.

QLife Inc. will store data in a secure server and within a vault for a period of 5 years. After this time, material will be disposed of such that it cannot be restored (e.g. chemical dissolution, deletion).

Tokyo Medical and Dental University will store the survey responses and analysis results in a locked cabinet for 10 years after the publication of the article.

2. Supplementary Results

## 2.1. Treatment with biologics or Janus kinase inhibitors (Bio/JAKi) and self-management (SMN)

Patients using Bio/JAKi show a similar demographic profile to those not using Bio/JAKi, although Bio/JAKi users tend to be younger, have a history of hospitalization and earn less money; Bio/JAKi users were also more likely to visit university hospitals and less likely to visit clinics for treatment (Table S1). An association between SMN and Bio/JAKi was only present for patients with UC (Fig. S2A). Specifically, patients with UC that use Bio/JAKi medications were 12% to 80% more likely to also practice SMN than patients not using Bio/JAKi (section 2.3). Independently of SMN, patients treated with Bio/JAKi were 5% to 22% more likely to be satisfied with their treatment than those not using Bio/JAKi (Fig. S2B).


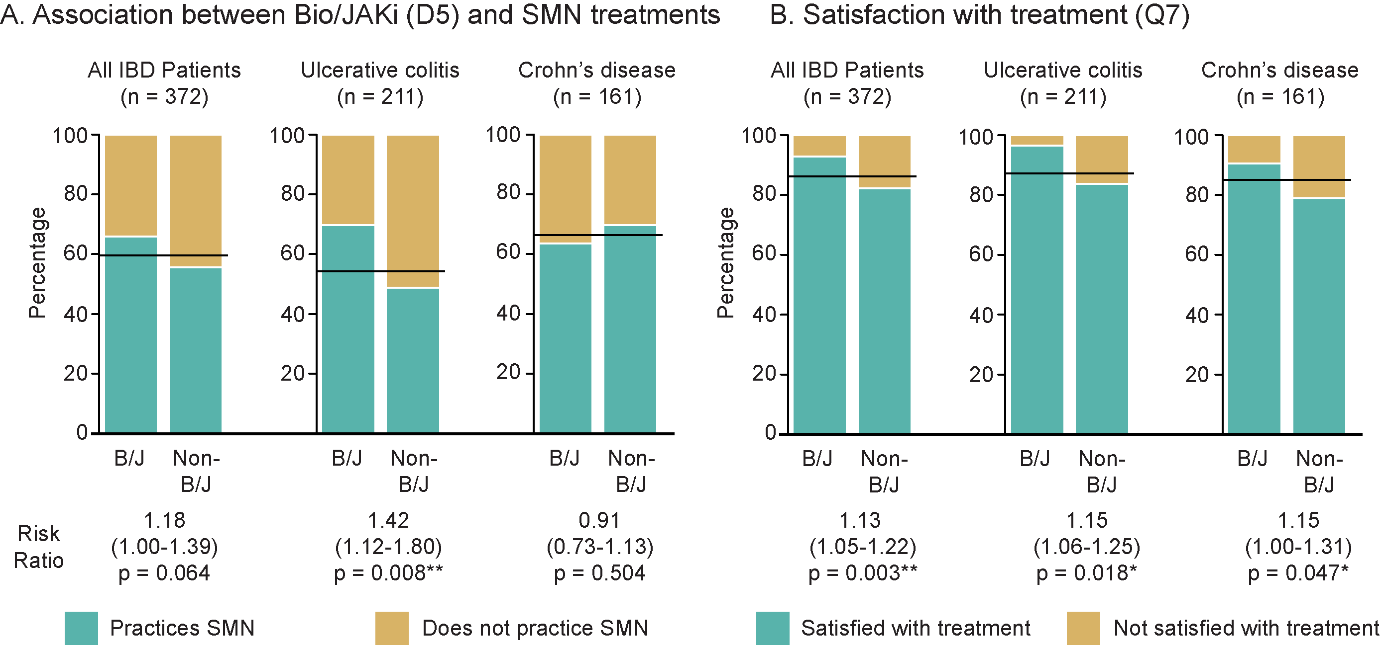


**Fig. S2. Associations involving treatment with Bio/JAKi. (A) The association between Bio/JAKi treatment and self-management practice. (B) The association between Bio/JAKi use and satisfaction with treatment. For complete wording of the questions, see S2 Text and Figures. Risk ratio (95% CI) and P-value for Fisher’s exact test are shown for each pairing. Black lines represent the expected proportions, independent of Bio/JAKi treatment. B/J: patients taking Bio/JAKi medications (as coded by response to D5). Bio/JAKi: biologics or Janus kinase inhibitors. CI: confidence interval. IBD: inflammatory bowel disease. Non-B/J: patients not taking Bio/JAKi medications. SMN: self-management.**

**Table S1. Patient characteristics by treatment with Bio/JAKi**

| **Characteristic, n (%)** | **Bio/JAKi  (n = 141)** | **Non-Bio/JAKi**  **(n = 231)** | **P value** |
| --- | --- | --- | --- |
| Age (years), mean ± SD | 39.6±11.2 | 43.4±11.9 | 0.002 |
| Sex |  |  | 0.831 |
| Male | 66 (46.8) | 112 (48.5) |  |
| Female | 75 (53.2) | 119 (51.5) |  |
| IBD duration (years), mean ± SD | 9.5 ± 8.4 | 10.7 ± 10.7 | 0.258 |
| IBD severity |  |  | 0.086 |
| Remission | 45 (31.9) | 71 (30.7) |  |
| Mild | 29 (20.6) | 75 (32.5) |  |
| Moderate | 54 (38.3) | 65 (28.1) |  |
| Severe | 4 (2.8) | 9 (3.9) |  |
| Unknown | 9 (6.4) | 11 (4.8) |  |
| Medical institution visited |  |  | 0.006 |
| University hospital | 60 (42.6) | 68 (29.4) |  |
| General hospital | 63 (44.7) | 111 (48.1) |  |
| Clinic | 17 (12.1) | 52 (22.5) |  |
| Others | 1 (0.7) | 0 (0.0) |  |
| Treatment |  |  |  |
| 5-ASA | 103 (73.1) | 206 (89.2) |  |
| Steroids | 19 (13.5) | 46 (19.9) |  |
| Immunomodulators | 39 (27.7) | 53 (22.9) |  |
| Immunosuppressants | 3 (2.1) | 2 (0.9) |  |
| Biologics | 131 (92.9) | 0 (0.0) |  |
| JAKi | 10 (7.1) | 0 (0.0) |  |
| Others ^a^ | 24 (17.0) | 29 (12.6) |  |
| Bowel movements during the past week |  |  | 0.167 |
| 0–2/day | 76 (53.9) | 109 (47.2) |  |
| 3–4/day | 34 (24.1) | 74 (32.0) |  |
| 5–9/day | 23 (16.3) | 42 (18.2) |  |
| ≥ 10/day | 8 (5.7) | 6 (2.6) |  |
| Relapses in the previous year |  |  | 0.253 |
| 0 | 58 (41.1) | 91 (39.4) |  |
| 1–2 | 59 (41.8) | 87 (37.7) |  |
| ≥ 3 | 13 (9.2) | 19 (8.2) |  |
| Always/chronic ≥ 6mo | 11 (7.8) | 34 (14.7) |  |
| History of hospitalization |  |  | <0.001 |
| No | 18 (12.8) | 90 (39.0) |  |
| Yes | 123 (87.2) | 141 (61.0) |  |
| History of surgery |  |  | 0.538 |
| No | 103 (73.1) | 176 (76.2) |  |
| Yes | 38 (27.0) | 55 (23.8) |  |
| Employed ^b^ |  |  | 0.062 |
| No | 44 (31.2) | 49 (21.2) |  |
| Yes | 90 (63.8) | 162 (70.1) |  |
| Other | 7 (5.0) | 20 (8.7) |  |
| Annual income |  |  | 0.042 |
| < 4 million yen | 103 (73.1) | 145 (62.8) |  |
| ≥ 4 million yen | 38 (27.0) | 86 (37.2) |  |

Bio/JAKi and Non-Bio/JAKi groups were compared using Fisher’s tests for categorical variables and t-tests for continuous variables (age, IBD duration).
^a^ Includes Ciprofloxacin, Metronidazole, Elemental diet, Hiroshima Kampo, Indigo Naturalis.
^b^ Test compared ‘No’ and ‘Yes’, but not ‘Other.’
5-ASA, mesalamine; Bio/JAKi, biologics or Janus kinase inhibitors; IBD, inflammatory bowel disease; JAKi, Janus kinase inhibitors; SD, standard deviation.

# 2.2. Desired support systems

The types of support that patients require were assessed with a direct question to mark all relevant sources from: financial and employment, symptom control (e.g. food, body), mental support, other. Participants could also provide a free description for their response, which was categorized by the authors.

In terms of existing support systems, all patients with IBD typically consulted doctors and family members for advice about lifestyle choices and treatment (Fig. S3). However, patients with CD tended to consult more with nurses and other patients with IBD than did patients with UC.


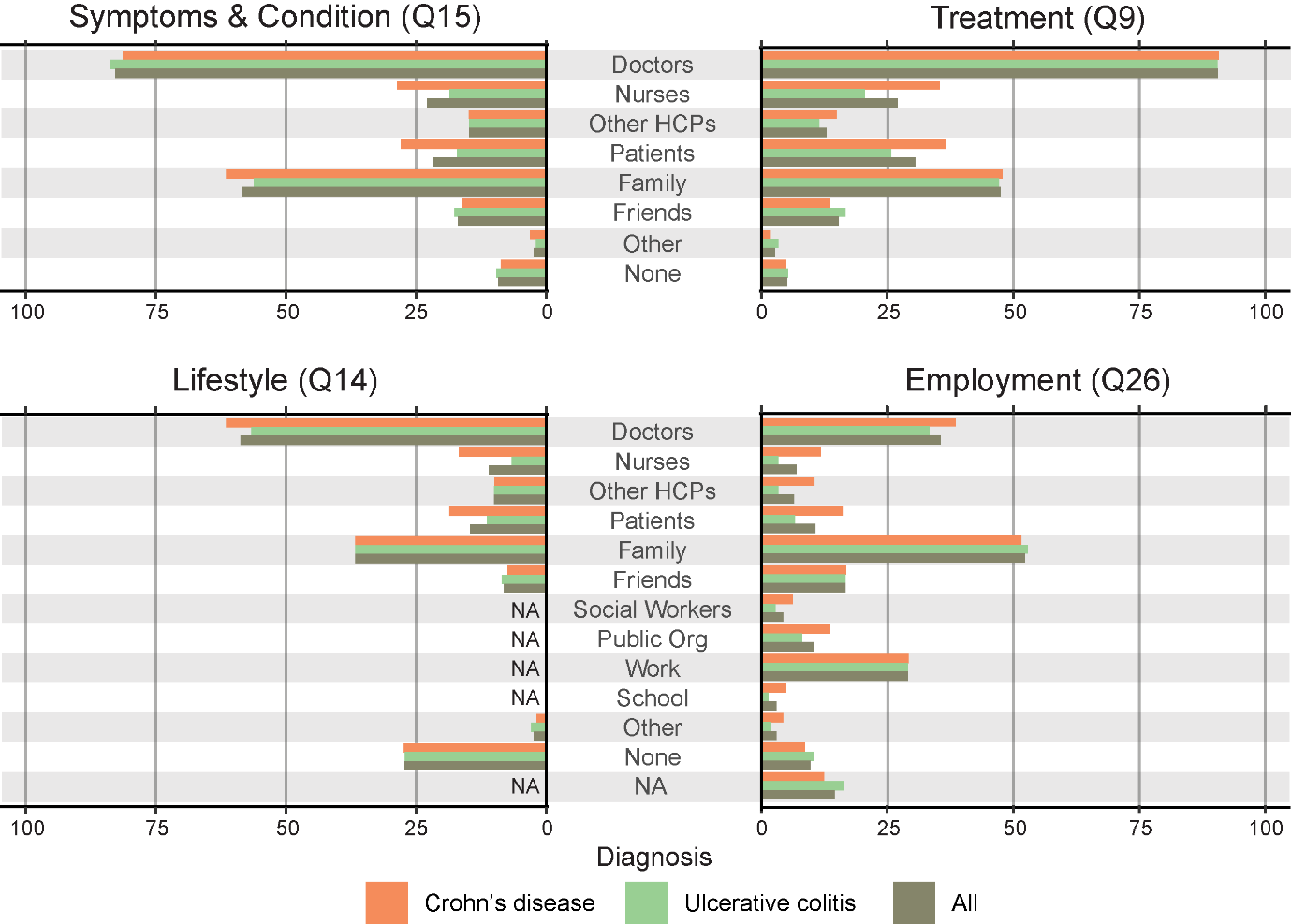
**Fig. S3.** Types of consultation partners. Data are based on a total of 372 patients with IBD, comprising 211 with UC and 161 with CD. For complete wording of the questions, see S2 Text and Figures. CD: Crohn’s disease. HCP: healthcare provider. IBD: inflammatory bowel disease. Org: organization/agency. UC: ulcerative colitis.

At a broad level, patients with IBD reported a wide range of desired support structures. Support from employers and financial assistance was the most desired, with up to 77% of patients (288/372) specifying some form of aid (Table S2). Patients further specified their needs from employers for mediation, equal employment, flexible working hours, and time off for medical appointments. In terms of financial aid, patients with IBD desired subsidies and exemptions concerning medical expenses and treatment costs. Moving on, up to 69% of patients were seeking support with symptom management (258/372) (Table S3). Here, participants desired better food-related information and access to expert support for symptom control. Finally, approximately 53% of responders desired mental health support (199/372) (Table S4). Responses suggest that patients with IBD want support from their community with access to helplines and support/advocacy groups. They also desired specialized consultation and discussion environments for IBD, as well as support with stress and anxiety.

**Table S2: Desired forms of employment and financial support reported by patients with IBD, n (%).** Results based on Q30: *What kind of support do you want to get for UC or CD? Please select all the support you would like to receive*: (financial/employment support, symptom management [food, body, etc], mental health support, Other), and Q31: *Please let us know what kind of support you have in mind for your selection in the previous question*: (free answer–no limit). Free answers categorized by authors. Main categories are in italics, stratified by sub-categories.

| **Item** | **UC**  **(n = 166)** | **CD**  **(n = 122)** |
| --- | --- | --- |
| *Employment* | *51 (30.7)* | *61 (50.0)* |
| Employment mediation | 17 (10.2) | 19 (15.6) |
| Flexible working hours | 13 (7.8) | 17 (13.9) |
| Equal employment for patients with intractable diseases | 14 (8.4) | 12 (9.8) |
| Allowing time off for medical appointments | 7 (4.2) | 13 (10.7) |
| *Medical expenses* | *77 (46.4)* | *33 (27.0)* |
| Subsidy/exemption of medical expenses | 43 (25.9) | 16 (13.1) |
| Reduced treatment costs | 18 (10.8) | 9 (7.4) |
| Increased allocations of public funds | 9 (5.4) | 4 (3.3) |
| Continuation of intractable disease designation | 7 (4.2) | 4 (3.3) |
| *Cost of living* | *24 (14.5)* | *20 (16.4)* |
| Living expenses support | 23 (13.9) | 15 (12.3) |
| Increase disability pension | 1 (0.6) | 3 (2.5) |
| Improve insurance coverage | 0 (0.0) | 3 (2.5) |
| *Community support* | *21 (12.7)* | *16 (13.1)* |
| Improve public awareness of IBD | 12 (7.2) | 13 (10.7) |
| Establishment of consultation space | 9 (5.4) | 3 (2.5) |

*CD: Crohn’s disease. IBD: inflammatory bowel disease. UC: ulcerative colitis.*

**Table S3: Desired forms of symptom management needs reported by patients with IBD, n (%).** Results based on Q30: *What kind of support do you want to get for UC or CD? Please select all the support you would like to receive*: (financial/employment support, symptom management [food, body, etc], mental health support, other), and Q31: *Please let us know what kind of support you have in mind for your selection in the previous question*: (free answer–no limit). Free answers categorized by authors. Main categories are in italics, stratified by sub-categories.

| **Item** | **UC**  **(n = 143)** | **CD**  **(n = 115)** |
| --- | --- | --- |
| *Food-related information* | *60 (42.0)* | *43 (37.4)* |
| Dietary restriction | 33 (23.1) | 17 (14.8) |
| Recipe information | 17 (11.9) | 20 (17.4) |
| Lipid and calorie control | 4 (2.8) | 5 (4.3) |
| Information on foods that fit IBD symptoms | 8 (5.6) | 2 (1.7) |
| *Symptom control* | *50 (35.0)* | *43 (37.4)* |
| What to do when symptoms worsen | 13 (9.1) | 8 (7.0) |
| Consultation services | 9 (6.3) | 12 (10.4) |
| Mobile application and online support | 7 (4.9) | 6 (5.2) |
| Access to information about IBD | 6 (4.2) | 6 (5.2) |
| Guidance for symptom improvement | 7 (4.9) | 6 (5.2) |
| Advice for living with IBD | 7 (4.9) | 2 (1.7) |
| Managing IBD-related physical conditions | 1 (0.7) | 3 (2.6) |
| *Patient-HCP communication* | *14 (9.8)* | *11 (9.6)* |
| Dietary guidance | 13 (9.1) | 8 (7.0) |
| Doctor's knowledge sharing | 1 (0.7) | 3 (2.6) |
| *Food services* | *12 (8.4)* | *12 (10.4)* |
| More food delivery services | 3 (2.1) | 7 (6.1) |
| Usage of IBD friendly ingredients | 6 (4.2) | 2 (1.7) |
| More food options when dining out | 3 (2.1) | 3 (2.6) |
| *Other* | *11 (7.7)* | *14 (12.2)* |
| New treatment agents | 2 (1.4) | 4 (3.5) |
| Patient-to-patient information | 0 (0.0) | 3 (2.6) |
| Guide for IBD management at work | 2 (1.4) | 0 (0.0) |
| Other | 7 (4.9) | 7 (6.1) |

*CD: Crohn’s disease. IBD: inflammatory bowel disease. UC: ulcerative colitis.*

**Table S4: Desired forms of mental health support reported by patients with IBD, n (%).** Results based on Q30: *What kind of support do you want to get for UC or CD? Please select all the support you would like to receive*: (financial/employment support, symptom management [food, body, etc], mental health support, Other), and Q31: *Please let us know what kind of support you have in mind for your selection in the previous question*: (free answer–no limit). Free answers categorized by authors. Main categories are in italics, stratified by sub-categories.

| **Item** | **UC**  **(n = 113)** | **CD**  **(n = 86)** |
| --- | --- | --- |
| *Medical* | *15 (13.3)* | *15 (17.4)* |
| Medical examination environment | 14 (12.4) | 14 (16.3) |
| Treatment cost subsidy | 1 (0.9) | 1 (1.2) |
| *Community* | *50 (44.2)* | *40 (46.5)* |
| Access to IBD helplines and support/advocacy groups | 17 (15.0) | 15 (17.4) |
| IBD patient-centred environment for consultation | 16 (14.2) | 11 (12.8) |
| Comfortable space to discuss IBD with other patients | 8 (7.1) | 7 (8.1) |
| Better understanding of IBD-related issues | 5 (4.4) | 3 (3.5) |
| Access to online support | 3 (2.7) | 2 (2.3) |
| Family cooperation | 1 (0.9) | 2 (2.3) |
| *Stress and anxiety* | *37 (32.7)* | *20 (23.3)* |
| Stress control | 16 (14.2) | 8 (9.3) |
| Support for anxiety | 14 (12.4) | 11 (12.8) |
| Relieve anxiety about the future | 6 (5.3) | 0 (0.0) |
| Anxiety about worsening symptoms | 2 (1.8) | 1 (1.2) |
| *Other* | *12 (10.6)* | *11 (12.8)* |

*CD: Crohn’s disease. IBD: inflammatory bowel disease. UC: ulcerative colitis.*

# 2.3. Contingency tables and t-tests

**SMN: Diagnosis (SC1)**

|  | CD | UC | Total |
| --- | --- | --- | --- |
| SMN | 107 | 115 | 222 |
| Non-SMN | 54 | 96 | 150 |
| Total | 161 | 211 | 372 |

**RR = 0.82 (0.70–0.97), *P* = 0.025**

**SMN: Quality-of-Life (Q33): All**

|  | Higher QOL | Lower QOL | Total |
| --- | --- | --- | --- |
| SMN | 205 | 17 | 222 |
| Non-SMN | 122 | 28 | 150 |
| Total | 327 | 45 | 372 |

**RR = 1.14 (1.04****–1.24), *P* = 0.002**

**SMN: Quality-of-Life (Q33): UC**

|  | Higher QOL | Lower QOL | Total |
| --- | --- | --- | --- |
| SMN | 108 | 7 | 115 |
| Non-SMN | 83 | 13 | 96 |
| Total | 191 | 20 | 211 |

**RR = 1.09 (0.99–1.19), *P* = 0.097**

**SMN: Quality-of-Life (Q33): CD**

|  | Higher QOL | Lower QOL | Total |
| --- | --- | --- | --- |
| SMN | 97 | 10 | 107 |
| Non-SMN | 39 | 15 | 54 |
| Total | 136 | 25 | 161 |

**RR = 1.26 (1.05–1.50), *P* = 0.005**

**SMN: Sex (D3): All**

|  | Female | Male | Total |
| --- | --- | --- | --- |
| SMN | 121 | 101 | 222 |
| Non-SMN | 73 | 77 | 150 |
| Total | 194 | 178 | 372 |

**RR = 1.12 (0.91–1.37), *P* = 0.291**

**SMN: Sex (D3): UC**

|  | Female | Male | Total |
| --- | --- | --- | --- |
| SMN | 75 | 40 | 115 |
| Non-SMN | 49 | 47 | 96 |
| Total | 124 | 87 | 211 |

**RR = 1.28 (1.01–1.62), *P* = 0.049**

**SMN: Sex (D3): CD**

|  | Female | Male | Total |
| --- | --- | --- | --- |
| SMN | 46 | 61 | 107 |
| Non-SMN | 24 | 30 | 54 |
| Total | 70 | 91 | 161 |

**RR = 0.97 (0.67–1.40), *P* = 0.868**

**SMN: Age (SC3): All**

|  | Mean | SD |
| --- | --- | --- |
| SMN | 41.4 | 11.7 |
| Non-SMN | 42.8 | 11.8 |

**t_318.91_ = 1.13, *P* = 0.260**

**SMN: Age (SC3): UC**

|  | Mean | SD |
| --- | --- | --- |
| SMN | 42.6 | 12.2 |
| Non-SMN | 45.4 | 11.4 |

**t_206.20_ = 1.73, *P* = 0.086**

**SMN: Age (SC3): CD**

|  | Mean | SD |
| --- | --- | --- |
| SMN | 40.1 | 11.1 |
| Non-SMN | 38.2 | 11.1 |

**t_107.03_ = 1.05, *P* = 0.295**

**SMN: Disease Duration (SC3 – D1): All**

|  | Mean | SD |
| --- | --- | --- |
| SMN | 10.7 | 10.3 |
| Non-SMN | 9.7 | 9.3 |

**t_339.68_ = 0.97, *P* = 0.332**

**SMN: Disease Duration (SC3 – D1): UC**

|  | Mean | SD |
| --- | --- | --- |
| SMN | 8.2 | 8.9 |
| Non-SMN | 9.4 | 8.9 |

**t_202.12_ = 0.99, *P* = 0.322**

**SMN: Disease Duration (SC3 – D1): CD**

|  | Mean | SD |
| --- | --- | --- |
| SMN | 13.3 | 11.1 |
| Non-SMN | 10.1 | 10.2 |

**t_114.90_ = 1.83, *P* = 0.071**

**SMN: Hospitalization (D9): All**

|  | Hospitalized | Not Hospitalized | Total |
| --- | --- | --- | --- |
| SMN | 171 | 51 | 222 |
| Non-SMN | 93 | 57 | 150 |
| Total | 264 | 108 | 372 |

**RR = 1.24 (1.08–1.44), *P* = 0.002**

**SMN: Hospitalization (D9): UC**

|  | Hospitalized | Not Hospitalized | Total |
| --- | --- | --- | --- |
| SMN | 71 | 44 | 115 |
| Non-SMN | 51 | 45 | 96 |
| Total | 122 | 89 | 211 |

**RR = 1.16 (0.92–1.47), *P* = 0.212**

**SMN: Hospitalization (D9): CD**

|  | Hospitalized | Not Hospitalized | Total |
| --- | --- | --- | --- |
| SMN | 100 | 7 | 107 |
| Non-SMN | 42 | 12 | 54 |
| Total | 142 | 19 | 161 |

**RR = 1.20 (1.03–1.40), *P* = 0.008**

**SMN: Surgery (D10): All**

|  | Surgery | No Surgery | Total |
| --- | --- | --- | --- |
| SMN | 65 | 157 | 222 |
| Non-SMN | 28 | 122 | 150 |
| Total | 93 | 279 | 372 |

**RR = 1.57 (1.06–2.32), *P* = 0.021**

**SMN: Surgery (D10): UC**

|  | Surgery | No Surgery | Total |
| --- | --- | --- | --- |
| SMN | 4 | 111 | 115 |
| Non-SMN | 6 | 90 | 96 |
| Total | 10 | 201 | 211 |

**RR = 0.56 (0.16–1.92), *P* = 0.518**

**SMN: Surgery (D10): CD**

|  | Surgery | No Surgery | Total |
| --- | --- | --- | --- |
| SMN | 61 | 46 | 107 |
| Non-SMN | 22 | 32 | 54 |
| Total | 83 | 78 | 161 |

**RR = 1.40 (0.97–2.01), *P* = 0.066**

**SMN: Dietary Record-Keeping (Q1): All**

|  | Frequent Records | Few to No Records | Total |
| --- | --- | --- | --- |
| SMN | 55 | 167 | 222 |
| Non-SMN | 13 | 137 | 150 |
| Total | 68 | 304 | 372 |

**RR = 2.86 (1.62–5.04), *P* < 0.001**

**SMN: Dietary Record-Keeping (Q1): UC**

|  | Frequent Records | Few to No Records | Total |
| --- | --- | --- | --- |
| SMN | 30 | 85 | 115 |
| Non-SMN | 5 | 91 | 96 |
| Total | 35 | 176 | 211 |

**RR = 5.01 (2.02–12.41), *P* < 0.001**

**SMN: Dietary Record-Keeping (Q1): CD**

|  | Frequent Records | Few to No Records | Total |
| --- | --- | --- | --- |
| SMN | 25 | 82 | 107 |
| Non-SMN | 8 | 46 | 54 |
| Total | 33 | 128 | 161 |

**RR = 1.58 (0.76–3.26), *P* = 0.223**

**SMN: Symptom Record-Keeping (Q16): All**

|  | Frequent Records | Few to No Records | Total |
| --- | --- | --- | --- |
| SMN | 66 | 156 | 222 |
| Non-SMN | 31 | 119 | 150 |
| Total | 97 | 275 | 372 |

**RR = 1.44 (0.99–2.09), *P* = 0.055**

**SMN: Symptom Record-Keeping (Q16): UC**

|  | Frequent Records | Few to No Records | Total |
| --- | --- | --- | --- |
| SMN | 40 | 75 | 115 |
| Non-SMN | 22 | 74 | 96 |
| Total | 62 | 149 | 211 |

**RR = 1.52 (0.97–2.37), *P* = 0.069**

**SMN: Symptom Record-Keeping (Q16): CD**

|  | Frequent Records | Few to No Records | Total |
| --- | --- | --- | --- |
| SMN | 26 | 81 | 107 |
| Non-SMN | 9 | 45 | 54 |
| Total | 35 | 126 | 161 |

**RR = 1.46 (0.74–2.89), *P* = =0.316**

**Bio/JAKi (D5): SMN: All**

|  | SMN | Non-SMN | Total |
| --- | --- | --- | --- |
| Bio/JAKi | 93 | 48 | 141 |
| No Bio/JAKi | 129 | 102 | 231 |
| Total | 222 | 150 | 372 |

**RR = 1.18 (1.00–1.39), *P* = 0.064**

**Bio/JAKi (D5): SMN: UC**

|  | SMN | Non-SMN | Total |
| --- | --- | --- | --- |
| Bio/JAKi | 39 | 17 | 56 |
| No Bio/JAKi | 76 | 79 | 155 |
| Total | 115 | 96 | 211 |

**RR = 1.42 (1.12–1.80), *P* = 0.008**

**Bio/JAKi (D5): SMN: CD**

|  | SMN | Non-SMN | Total |
| --- | --- | --- | --- |
| Bio/JAKi | 54 | 31 | 85 |
| No Bio/JAKi | 53 | 23 | 76 |
| Total | 107 | 54 | 161 |

**RR = 0.91 (0.73–1.13), *P* = 0.504**

**SMN: Treatment Satisfaction (Q7): All**

|  | Satisfied | Not Satisfied | Total |
| --- | --- | --- | --- |
| SMN | 199 | 23 | 222 |
| Non-SMN | 122 | 28 | 150 |
| Total | 321 | 51 | 372 |

**RR=1.10 (1.01–1.20), *P*=0.031**

**SMN: Treatment Satisfaction (Q7): UC**

|  | Satisfied | Not Satisfied | Total |
| --- | --- | --- | --- |
| SMN | 106 | 9 | 115 |
| Non-SMN | 78 | 18 | 96 |
| Total | 184 | 27 | 211 |

**RR=1.13 (1.02–1.27), *P*=0.023**

**SMN: Treatment Satisfaction (Q7): CD**

|  | Satisfied | Not Satisfied | Total |
| --- | --- | --- | --- |
| SMN | 93 | 14 | 107 |
| Non-SMN | 44 | 10 | 54 |
| Total | 137 | 24 | 161 |

**RR=1.07 (0.92–1.24), *P*=0.360**

**SMN: Confidence Managing Symptoms (Q19): All**

|  | Confident | Not Confident | Total |
| --- | --- | --- | --- |
| SMN | 160 | 62 | 222 |
| Non-SMN | 74 | 76 | 150 |
| Total | 234 | 138 | 372 |

**RR=1.46 (1.22–1.75), *P*<0.001**

**SMN: Confidence Managing Symptoms (Q19): UC**

|  | Confident | Not Confident | Total |
| --- | --- | --- | --- |
| SMN | 80 | 35 | 115 |
| Non-SMN | 45 | 51 | 96 |
| Total | 125 | 86 | 211 |

**RR=1.48 (1.16–1.90), *P*=0.001**

**SMN: Confidence Managing Symptoms (Q19): CD**

|  | Confident | Not Confident | Total |
| --- | --- | --- | --- |
| SMN | 80 | 27 | 107 |
| Non-SMN | 29 | 25 | 54 |
| Total | 109 | 52 | 161 |

**RR=1.39 (1.06–1.83), *P*=0.012**

**SMN: Ability to Manage Stress (Q22): All**

|  | Manages | Does Not Manage | Total |
| --- | --- | --- | --- |
| SMN | 124 | 98 | 222 |
| Non-SMN | 62 | 88 | 150 |
| Total | 186 | 186 | 372 |

**RR=1.35 (1.08–1.69), *P*=0.008**

**SMN: Ability to Manage Stress (Q22): UC**

|  | Manages | Does Not Manage | Total |
| --- | --- | --- | --- |
| SMN | 72 | 43 | 115 |
| Non-SMN | 39 | 57 | 96 |
| Total | 111 | 100 | 211 |

**RR=1.54 (1.16–2.04), *P*=0.002**

**SMN: Ability to Manage Stress (Q22): CD**

|  | Manages | Does Not Manage | Total |
| --- | --- | --- | --- |
| SMN | 52 | 55 | 107 |
| Non-SMN | 23 | 31 | 54 |
| Total | 75 | 86 | 161 |

**RR=1.14 (0.79–1.65), *P=0*.507**

**SMN: Treatment Continuation (Q12): All**

|  | Continuing | Not Continuing | Total |
| --- | --- | --- | --- |
| SMN | 217 | 5 | 222 |
| Non-SMN | 139 | 11 | 150 |
| Total | 356 | 16 | 372 |

**RR=1.05 (1.00–1.11), *P*=0.034**

**SMN: Treatment Continuation (Q12): UC**

|  | Continuing | Not Continuing | Total |
| --- | --- | --- | --- |
| SMN | 112 | 3 | 115 |
| Non-SMN | 91 | 5 | 96 |
| Total | 203 | 8 | 211 |

**RR=1.03 (0.97–1.09), *P*=0.473**

**SMN: Treatment Continuation (Q12): CD**

|  | Continuing | Not Continuing | Total |
| --- | --- | --- | --- |
| SMN | 105 | 2 | 107 |
| Non-SMN | 48 | 6 | 54 |
| Total | 153 | 8 | 161 |

**RR=1.10 (1.00–1.22), *P*=0.018**

**SMN: Treatment Consultation (Q9): All**

|  | Consults | Does Not Consult | Total |
| --- | --- | --- | --- |
| SMN | 213 | 9 | 222 |
| Non-SMN | 140 | 10 | 150 |
| Total | 353 | 19 | 372 |

**RR=1.03 (0.98–1.08), *P*=0.338**

**SMN: Treatment Consultation (Q9): UC**

|  | Consults | Does Not Consult | Total |
| --- | --- | --- | --- |
| SMN | 110 | 5 | 115 |
| Non-SMN | 90 | 6 | 96 |
| Total | 200 | 11 | 211 |

**RR=1.02 (0.96–1.09), *P*=0.552**

**SMN: Treatment Consultation (Q9): CD**

|  | Consults | Does Not Consult | Total |
| --- | --- | --- | --- |
| SMN | 103 | 4 | 107 |
| Non-SMN | 50 | 4 | 54 |
| Total | 153 | 8 | 161 |

**RR=1.04 (0.96–1.13), *P*=0.443**

**SMN: Treatment Consultation (Q9): All**

|  | Consults with ≥ 3 | Consults with < 3 | Total |
| --- | --- | --- | --- |
| SMN | 100 | 122 | 222 |
| Non-SMN | 47 | 103 | 150 |
| Total | 147 | 225 | 372 |

**RR=1.44 (1.09–1.90), *P*=0.009**

**SMN: Treatment Consultation (Q9): UC**

|  | Consults with ≥ 3 | Consults with < 3 | Total |
| --- | --- | --- | --- |
| SMN | 45 | 70 | 115 |
| Non-SMN | 28 | 68 | 96 |
| Total | 73 | 138 | 211 |

**RR=1.34 (0.91–1.97), *P*=0.147**

**SMN: Treatment Consultation (Q9): CD**

|  | Consults with ≥ 3 | Consults with < 3 | Total |
| --- | --- | --- | --- |
| SMN | 55 | 52 | 107 |
| Non-SMN | 19 | 35 | 54 |
| Total | 74 | 87 | 161 |

**RR=1.46 (0.97–2.19), *P*=0.065**

**SMN: Lifestyle Consultation (Q14): All**

|  | Consults | Does Not Consult | Total |
| --- | --- | --- | --- |
| SMN | 178 | 44 | 222 |
| Non-SMN | 93 | 57 | 150 |
| Total | 271 | 101 | 372 |

**RR=1.29 (1.12–1.49), *P*<0.001**

**SMN: Lifestyle Consultation (Q14): UC**

|  | Consults | Does Not Consult | Total |
| --- | --- | --- | --- |
| SMN | 95 | 20 | 115 |
| Non-SMN | 59 | 37 | 96 |
| Total | 154 | 57 | 211 |

**RR=1.34 (1.12–1.61), *P*=0.001**

**SMN: Lifestyle Consultation (Q14): CD**

|  | Consults | Does Not Consult | Total |
| --- | --- | --- | --- |
| SMN | 83 | 24 | 107 |
| Non-SMN | 34 | 20 | 54 |
| Total | 117 | 44 | 161 |

**RR=1.23 (0.98–1.55), *P*=0.062**

**SMN: Lifestyle Consultation (Q14): All**

|  | Consults with ≥ 3 | Consults with < 3 | Total |
| --- | --- | --- | --- |
| SMN | 50 | 172 | 222 |
| Non-SMN | 14 | 136 | 150 |
| Total | 64 | 308 | 372 |

**RR=2.41 (1.38–4.20), *P*=0.001**

**SMN: Lifestyle Consultation (Q14): UC**

|  | Consults with ≥ 3 | Consults with < 3 | Total |
| --- | --- | --- | --- |
| SMN | 24 | 91 | 115 |
| Non-SMN | 5 | 91 | 96 |
| Total | 29 | 182 | 211 |

**RR=4.01 (1.59–10.10), *P*=0.001**

**SMN: Lifestyle Consultation (Q14): CD**

|  | Consults with ≥ 3 | Consults with < 3 | Total |
| --- | --- | --- | --- |
| SMN | 26 | 81 | 107 |
| Non-SMN | 9 | 45 | 54 |
| Total | 35 | 126 | 161 |

**RR=1.46 (0.74–2.89), *P*=0.316**

**SMN: Condition Consultation (Q15): All**

|  | Consults | Does Not Consult | Total |
| --- | --- | --- | --- |
| SMN | 210 | 12 | 222 |
| Non-SMN | 128 | 22 | 150 |
| Total | 338 | 34 | 372 |

**RR=1.11 (1.03–1.19), *P*=.003**

**SMN: Condition Consultation (Q15): UC**

|  | Consults | Does Not Consult | Total |
| --- | --- | --- | --- |
| SMN | 110 | 5 | 115 |
| Non-SMN | 81 | 15 | 96 |
| Total | 191 | 20 | 211 |

**RR=1.13 (1.03–1.25), *P*=0.008**

**SMN: Condition Consultation (Q15): CD**

|  | Consults | Does Not Consult | Total |
| --- | --- | --- | --- |
| SMN | 100 | 7 | 107 |
| Non-SMN | 47 | 7 | 54 |
| Total | 147 | 14 | 161 |

**RR=1.07 (0.96–1.20), *P*=0.235**

**SMN: Condition Consultation (Q15): All**

|  | Consults with ≥ 3 | Consults with < 3 | Total |
| --- | --- | --- | --- |
| SMN | 94 | 128 | 222 |
| Non-SMN | 49 | 101 | 150 |
| Total | 143 | 229 | 372 |

**RR=1.30 (0.98–1.71), *P*=0.065**

**SMN: Condition Consultation (Q15): UC**

|  | Consults with ≥ 3 | Consults with < 3 | Total |
| --- | --- | --- | --- |
| SMN | 44 | 71 | 115 |
| Non-SMN | 28 | 68 | 96 |
| Total | 72 | 139 | 211 |

**RR=1.31 (0.89–1.93), *P*=0.191**

**SMN: Condition Consultation (Q15): CD**

|  | Consults with ≥ 3 | Consults with < 3 | Total |
| --- | --- | --- | --- |
| SMN | 50 | 57 | 107 |
| Non-SMN | 21 | 33 | 54 |
| Total | 71 | 90 | 161 |

**RR=1.20 (0.81–1.78), *P*=0.402**

**SMN: Employment Consultation (Q26): All**

|  | Consults | Does Not Consult | Total |
| --- | --- | --- | --- |
| SMN | 169 | 53 | 222 |
| Non-SMN | 113 | 37 | 150 |
| Total | 282 | 90 | 372 |

**RR=1.01 (0.90–1.14), *P*=0.902**

**SMN: Employment Consultation (Q26): UC**

|  | Consults | Does Not Consult | Total |
| --- | --- | --- | --- |
| SMN | 84 | 31 | 115 |
| Non-SMN | 71 | 25 | 96 |
| Total | 155 | 56 | 211 |

**RR=0.99 (0.84–1.16), *P*=1.000**

**SMN: Employment Consultation (Q26): CD**

|  | Consults | Does Not Consult | Total |
| --- | --- | --- | --- |
| SMN | 85 | 22 | 107 |
| Non-SMN | 42 | 12 | 54 |
| Total | 127 | 34 | 161 |

**RR=1.02 (0.86–1.21), *P*=0.839**

**SMN: Employment Consultation (Q26): All**

|  | Consults with ≥ 3 | Consults with < 3 | Total |
| --- | --- | --- | --- |
| SMN | 76 | 146 | 222 |
| Non-SMN | 33 | 117 | 150 |
| Total | 109 | 263 | 372 |

**RR=1.56 (1.09–2.21), *P*=0.015**

**SMN: Employment Consultation (Q26): UC**

|  | Consults with ≥ 3 | Consults with < 3 | Total |
| --- | --- | --- | --- |
| SMN | 34 | 81 | 115 |
| Non-SMN | 17 | 79 | 96 |
| Total | 51 | 160 | 211 |

**RR=1.67 (1.00–2.80), *P*=0.053**

**SMN: Employment Consultation (Q26): CD**

|  | Consults with ≥ 3 | Consults with < 3 | Total |
| --- | --- | --- | --- |
| SMN | 42 | 65 | 107 |
| Non-SMN | 16 | 38 | 54 |
| Total | 58 | 103 | 161 |

**RR=1.32 (0.82–2.13), *P*=0.297**

**SMN: Help from Understanding Symptoms (Q17): All**

|  | Helps | Does Not Help | Total |
| --- | --- | --- | --- |
| SMN | 189 | 33 | 222 |
| Non-SMN | 109 | 41 | 150 |
| Total | 298 | 74 | 372 |

**RR=1.17 (1.05–1.31), *P*=0.004**

**SMN: Help from Understanding Symptoms (Q17): UC**

|  | Helps | Does Not Help | Total |
| --- | --- | --- | --- |
| SMN | 104 | 11 | 115 |
| Non-SMN | 71 | 25 | 96 |
| Total | 175 | 36 | 211 |

**RR=1.22 (1.07–1.40), *P*=0.002**

**SMN: Help from Understanding Symptoms (Q17): CD**

|  | Helps | Does Not Help | Total |
| --- | --- | --- | --- |
| SMN | 85 | 22 | 107 |
| Non-SMN | 38 | 16 | 54 |
| Total | 123 | 38 | 161 |

***RR=1.13 (0.93–1.38), P=0.239***

*CD: Crohn’s disease. QOL: quality of life. RR: risk ratio. SMN: self-management. UC: ulcerative colitis.*
