# Supplementary material for: A web-based survey on self-management for patients with inflammatory bowel disease in Japan
Source: PLoS One. 2023 Jul 17;18(7):e0287618. doi: 10.1371/journal.pone.0287618 (PMC10351702; doi:10.1371/journal.pone.0287618)
Supplement: S2 Text — Full English version of the survey, showing frequency distribution of responses for each item, stratified by diagnosis. (DOCX) [file pone.0287618.s002.docx]

A web-based survey on self-management for patients with inflammatory bowel disease in Japan

*Masakazu Nagahori, Takahito Imai, Mikiko Nakashoji, Ai Tairaka, Jovelle L Fernandez*

Full Survey and Responses

**(single-answer):** one response allowed
**(multi-answer):** multiple responses allowed
**(free-answer):** response given in textbox and coded by authors. The text responses supporting this analysis are available from the corresponding author on reasonable request.

# Screening questions

The following questions were used to identify the study population. Participants who responded with any of the bold items for each of these questions were included in the main analysis.

**SC1.** For which of the following diseases have you been diagnosed in a hospital? **(multi-answer)**

- 1. Atopic dermatitis
  2. Allergic rhinitis
  3. **Ulcerative colitis**
  4. Irritable bowel syndrome
  5. Reflux esophagitis
  6. **Crohn’s disease**
  7. Asthma
  8. Chronic acute gastritis
  9. Chronic diarrhoea
  10. Chronic constipation
  11. None of the above

**SC2.** How often do you visit a hospital/clinic to treat UC or CD? **(single-answer)**

- 1. **Once a week.**
  2. **Once every 2 weeks.**
  3. **Once every 3 weeks.**
  4. **Once every 4 weeks (every month)**
  5. **Once every 2 months.**
  6. **Once every 3 months.**
  7. Once every four months or more.

**SC3.** Please tell us your current age. **(single-answer)**

(0–99) **(≥ 20)**

# Demographic questions

**D1.** Please tell us the age of onset of UC or CD. **(single-answer)**

(0–99)

*(age–age of onset = disease duration)*

**
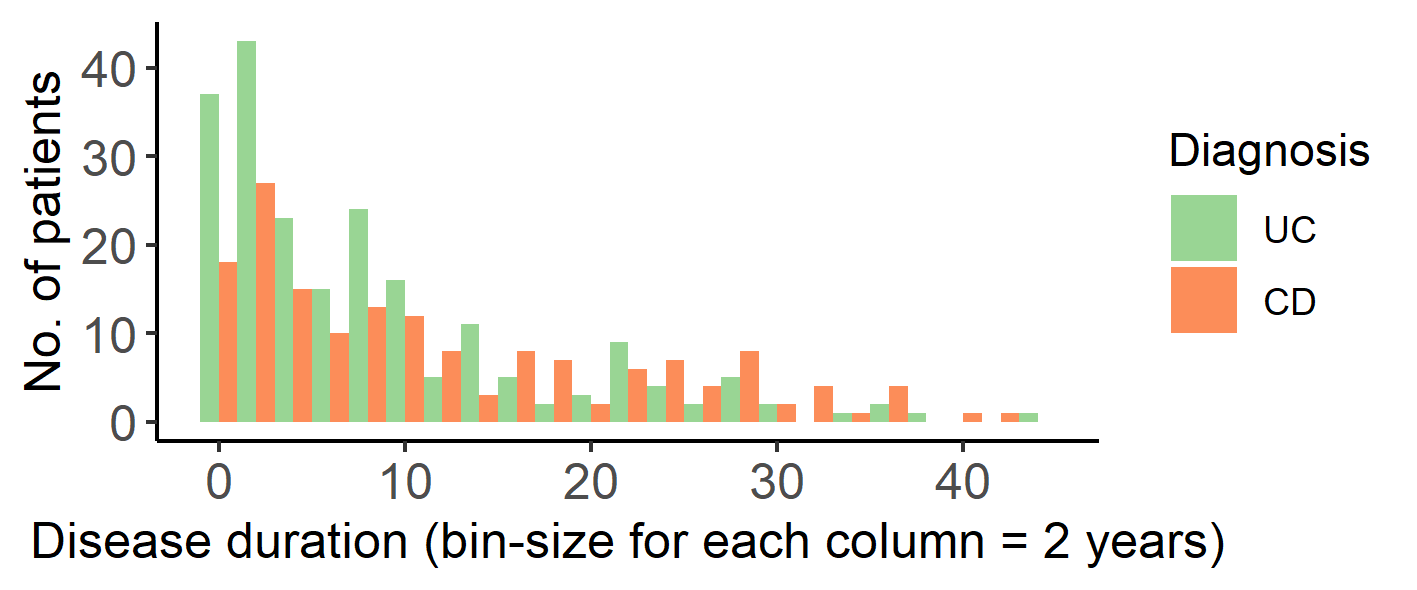
**

**D2.** Please rate the current severity of your condition. (If there is anything told by your doctor, please tell it to us.) **(single-answer)**


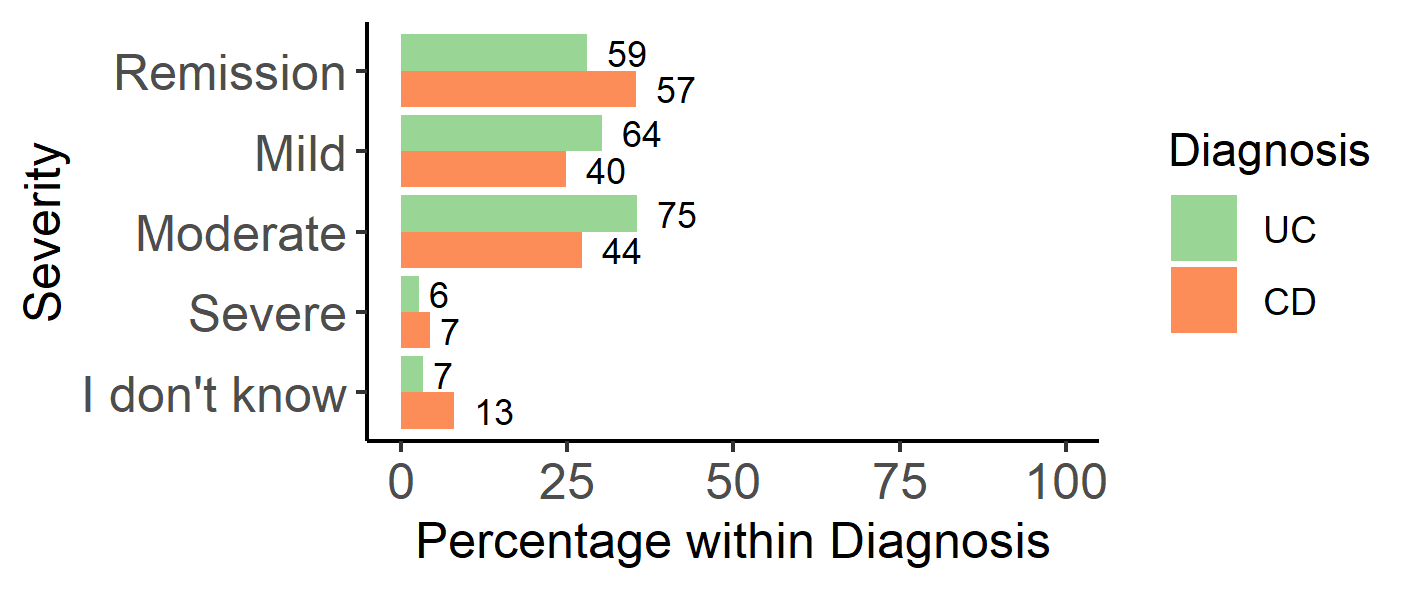


**D3.** Please tell us your sex. **(single-answer)**


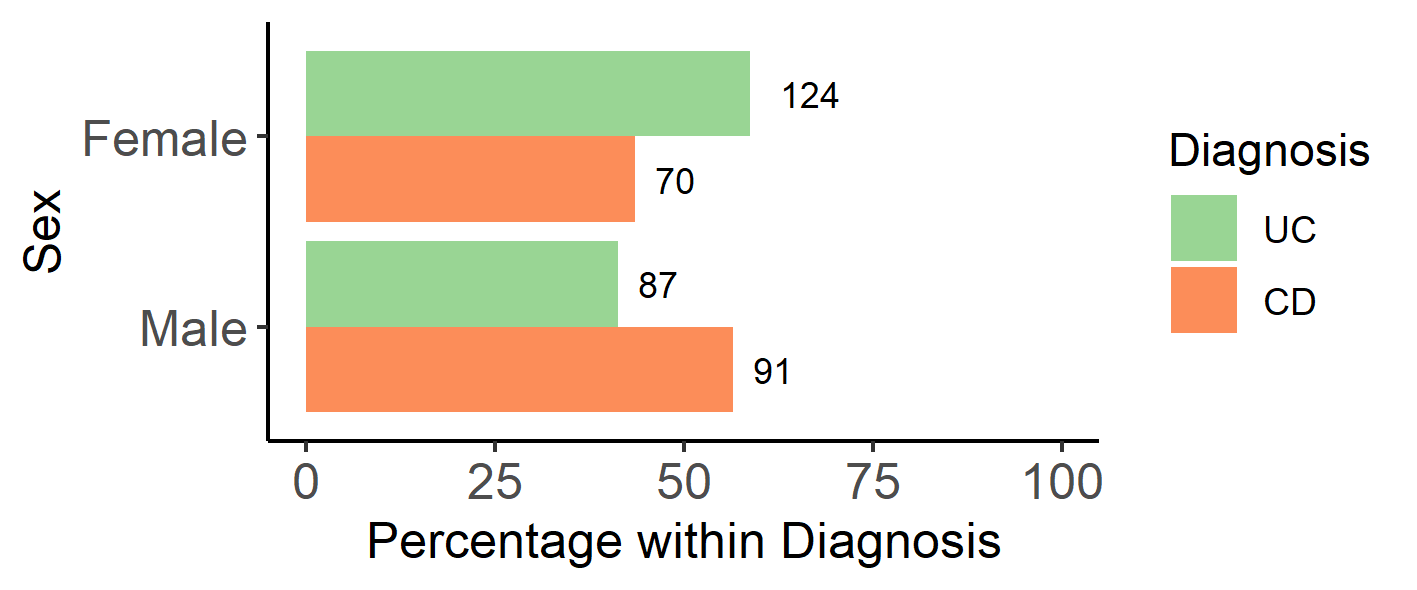


**D4.** What kind of medical institution do you usually visit? **(single-answer)**


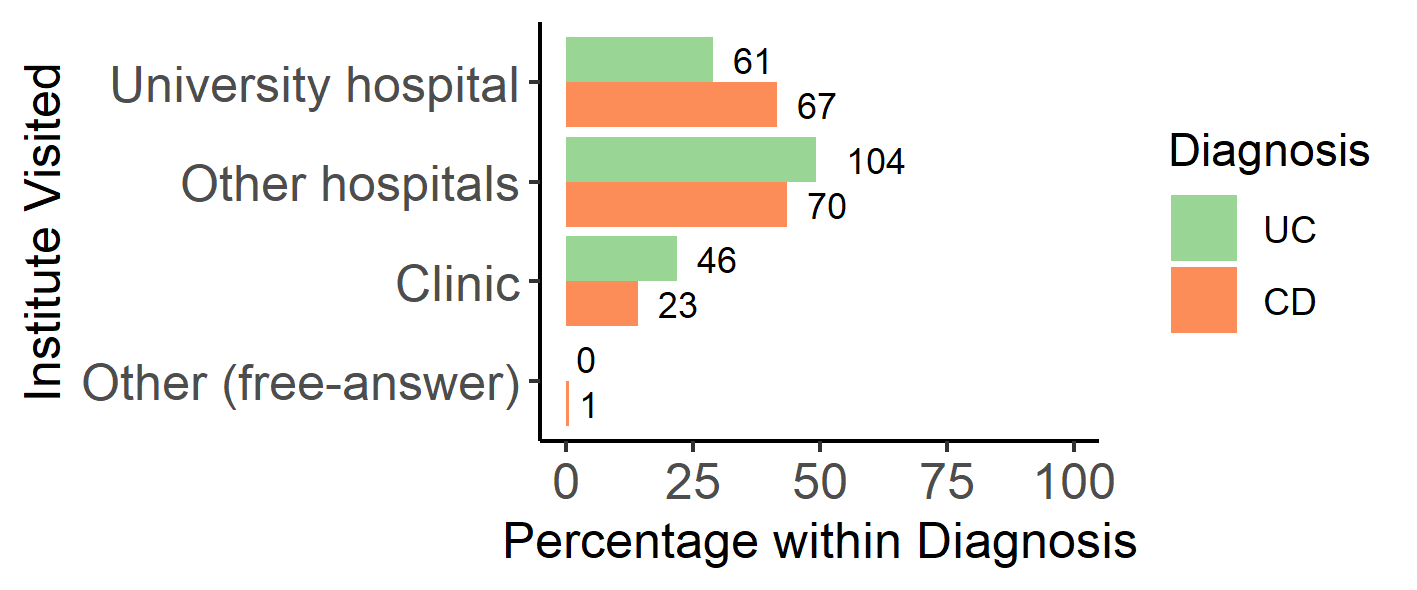


**D5.** What drugs are you currently prescribed for the treatment of UC or CD? Please enter the name of the drug. **(free-answer)**


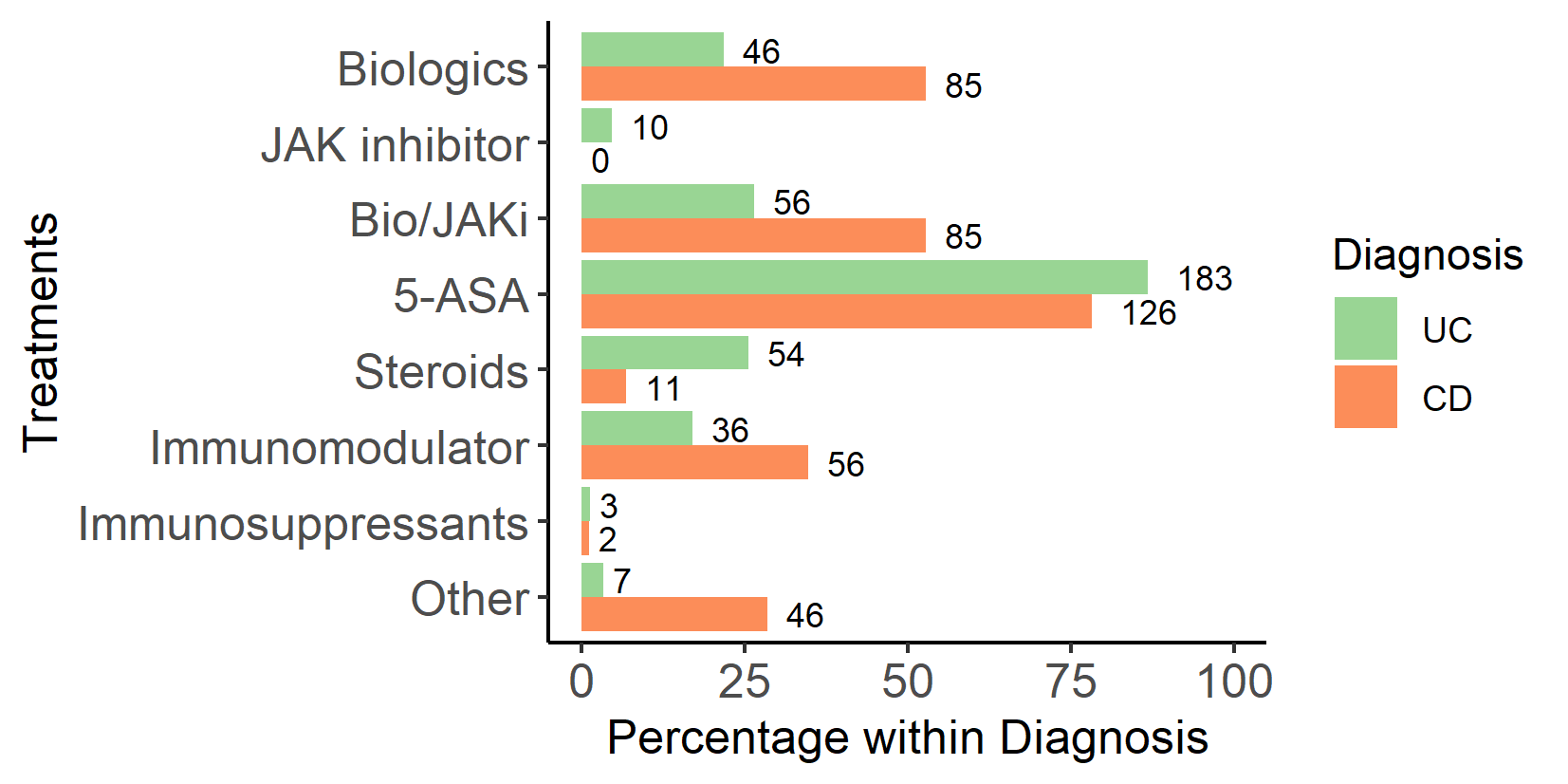


**D6.** What is the average number of bowel movements per day in the past week? **(single-answer)**


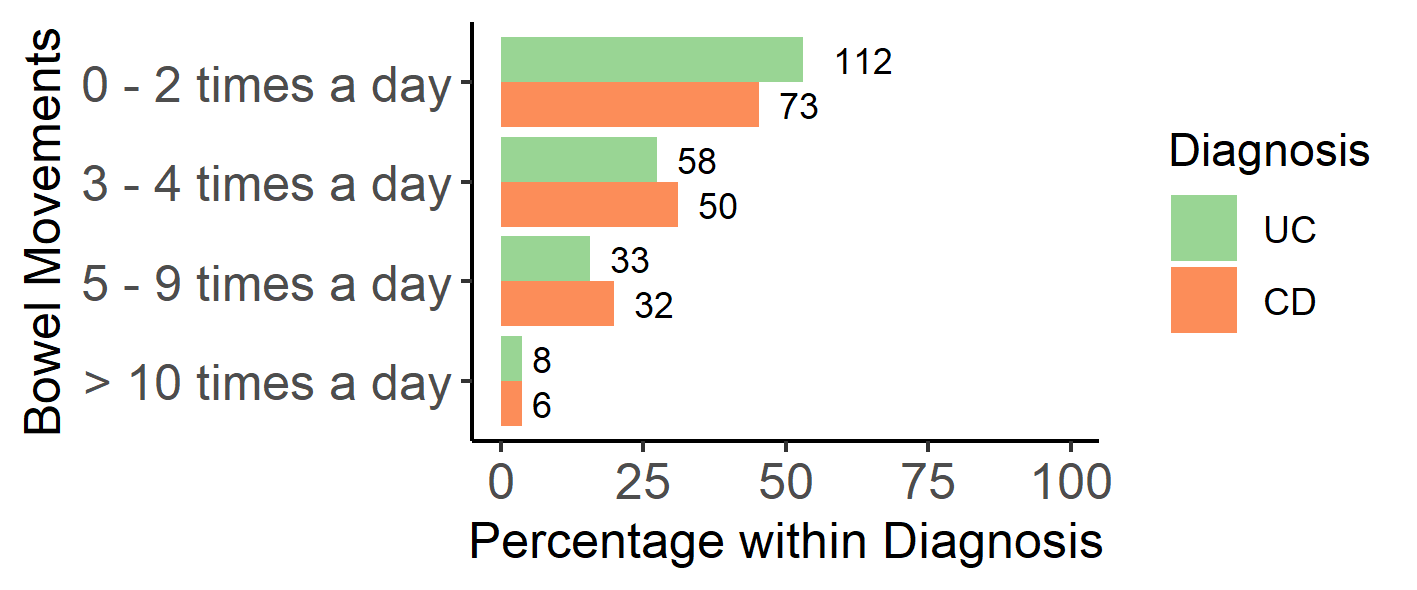


**D7.** How was your fever for the past week? **(single-answer)**

**
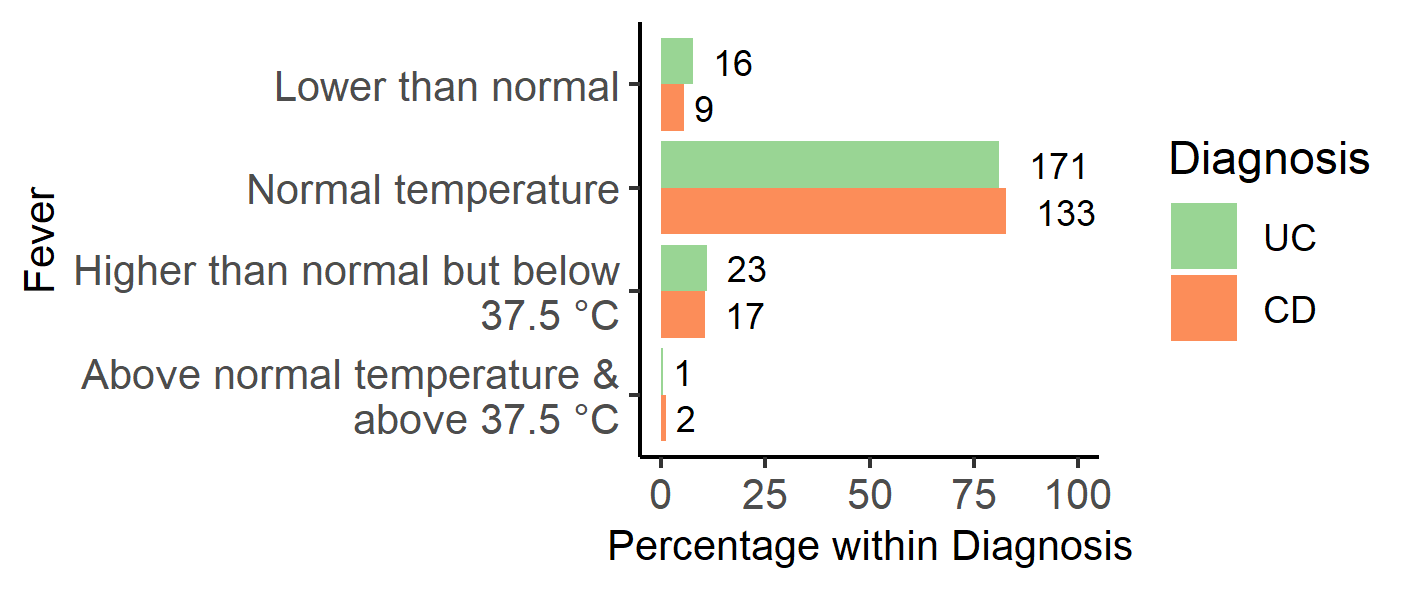
**

**D8.** How many relapses of UC/CD have occurred in the 1 year (365 days) before now?
**(single-answer)**


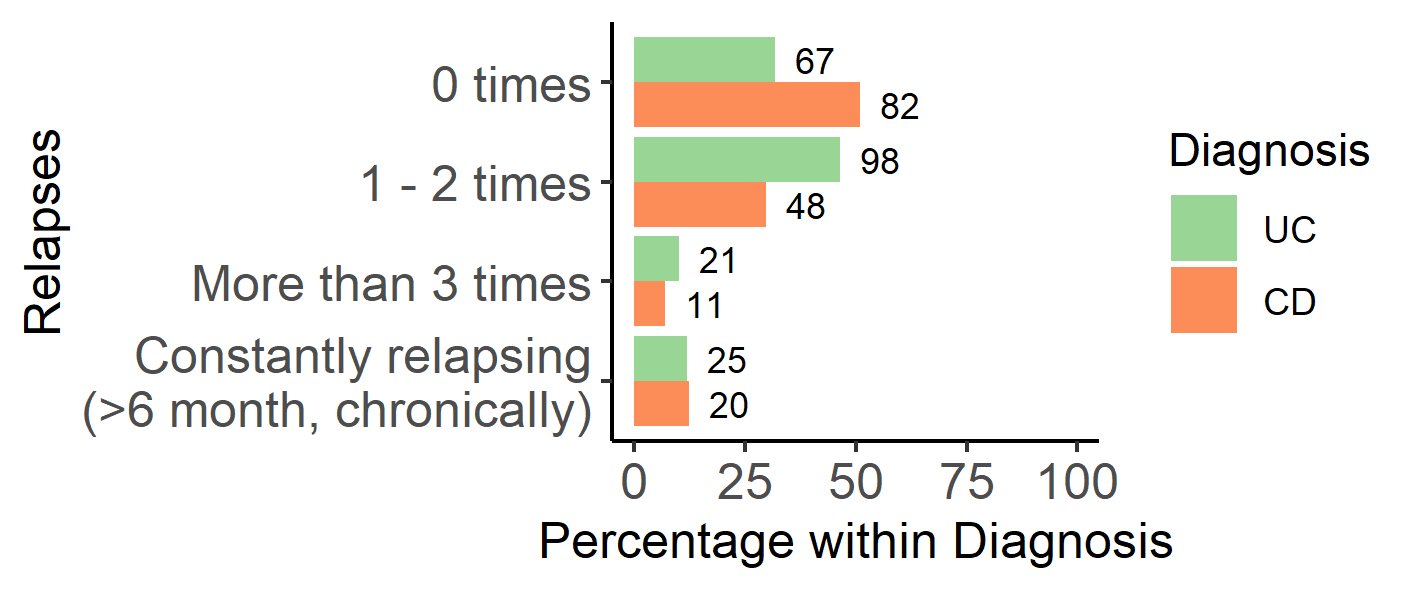


**D9.** Have you ever been hospitalized for treatment of UC or CD? **(single-answer)**


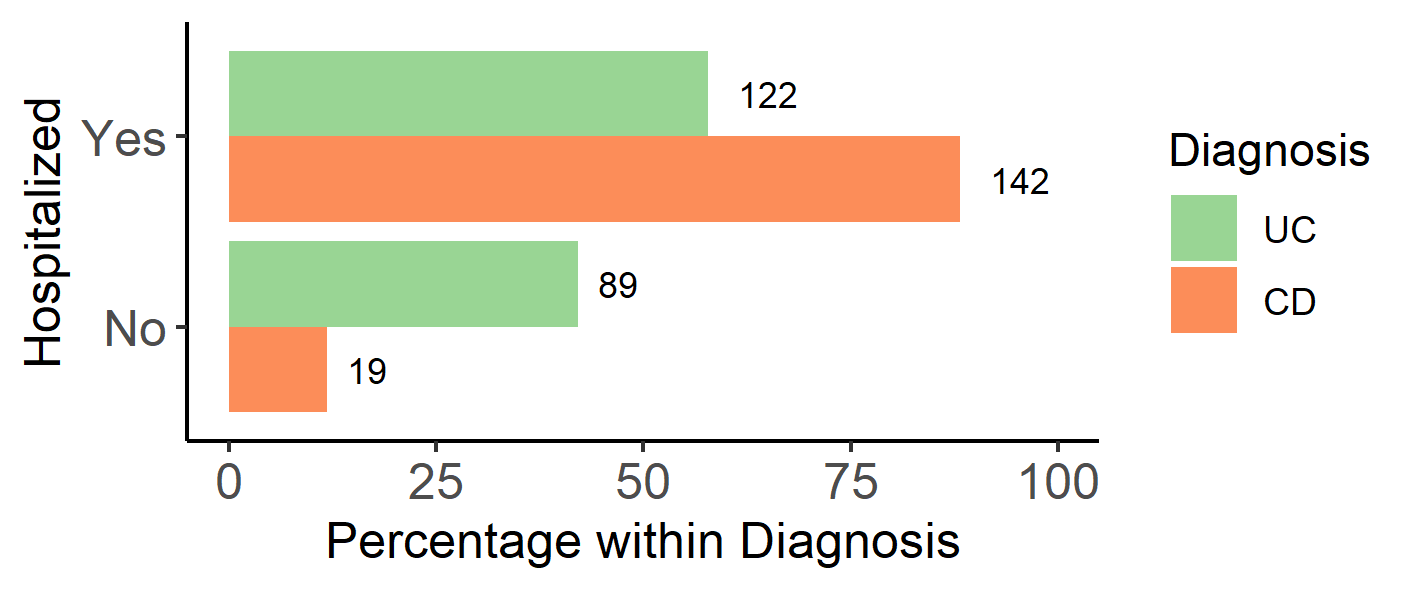


**D10.** Have you ever had surgery (resection of the intestine) to treat UC or CD? **(single-answer)**


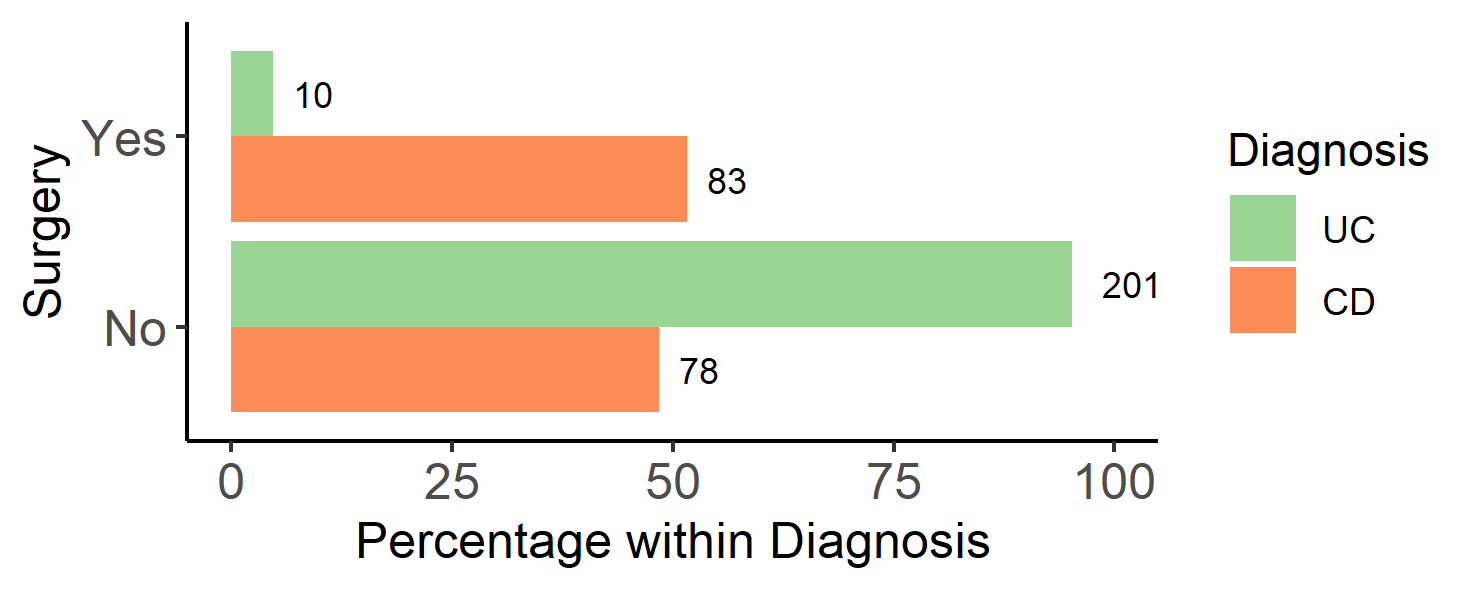


**D11.** Please tell us how you work now. **(single-answer)**

**
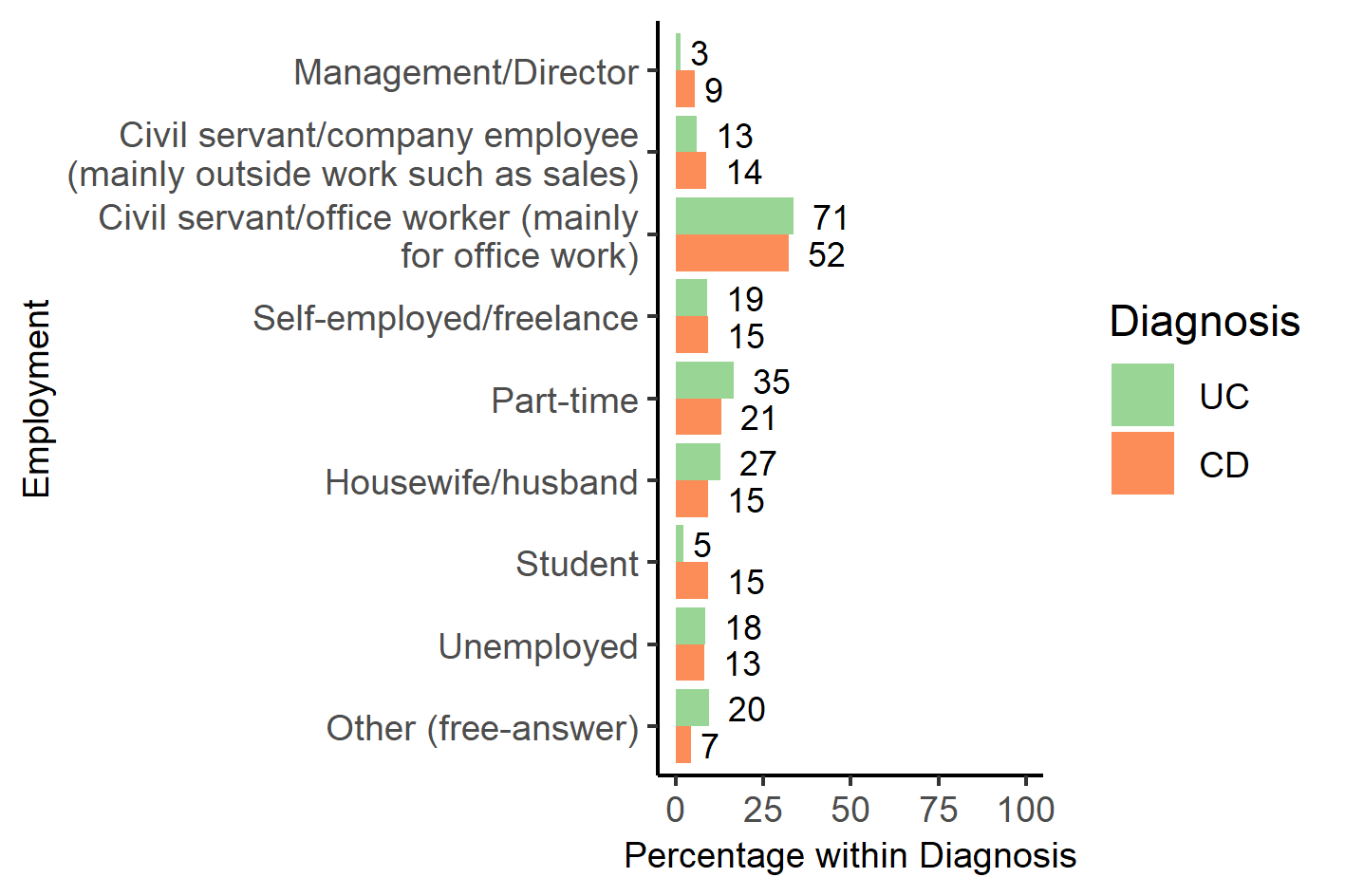
**

**D12.** Please tell us your current annual income as an individual. **(single-answer)**


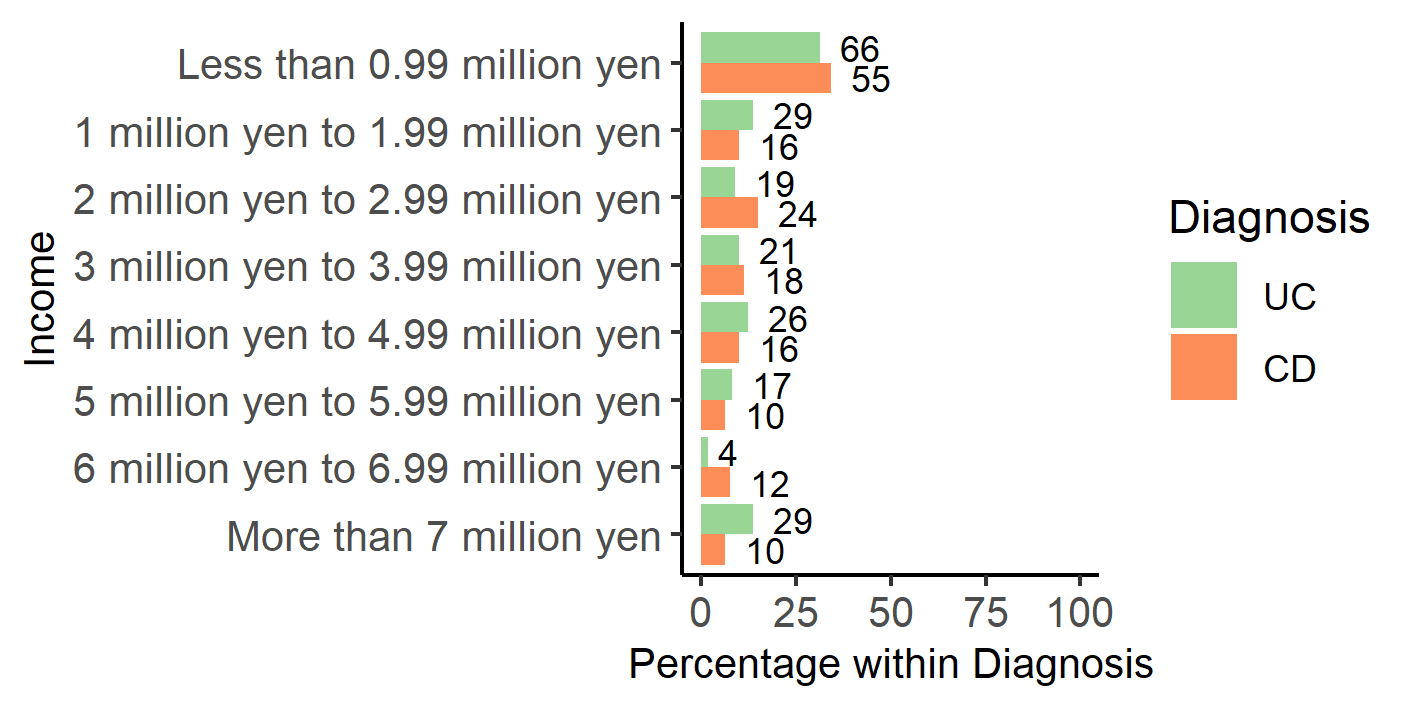


# Main Survey

The following items constitute the main analytical questions. Some items were classified into positive or negative responses, and categorical boundaries are represented here by (+) or (-). Note that these boundaries were not indicated to the participants.

## Part I: Diet

1. Do you keep a record of your meals (diary, photos, etc.)? **(single-answer)**


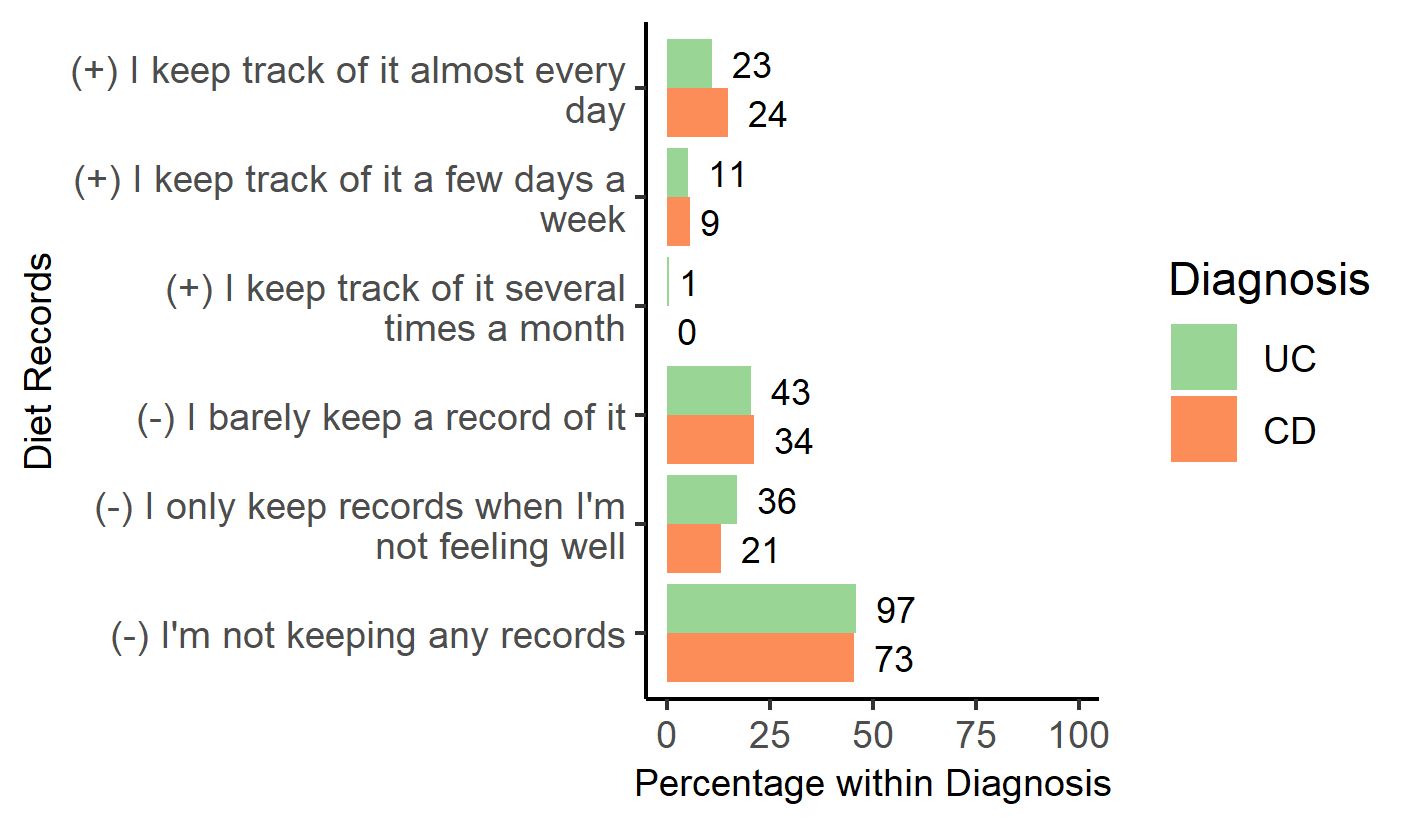


1. How often do you check the nutrition labelling when buying food? **(single-answer)**


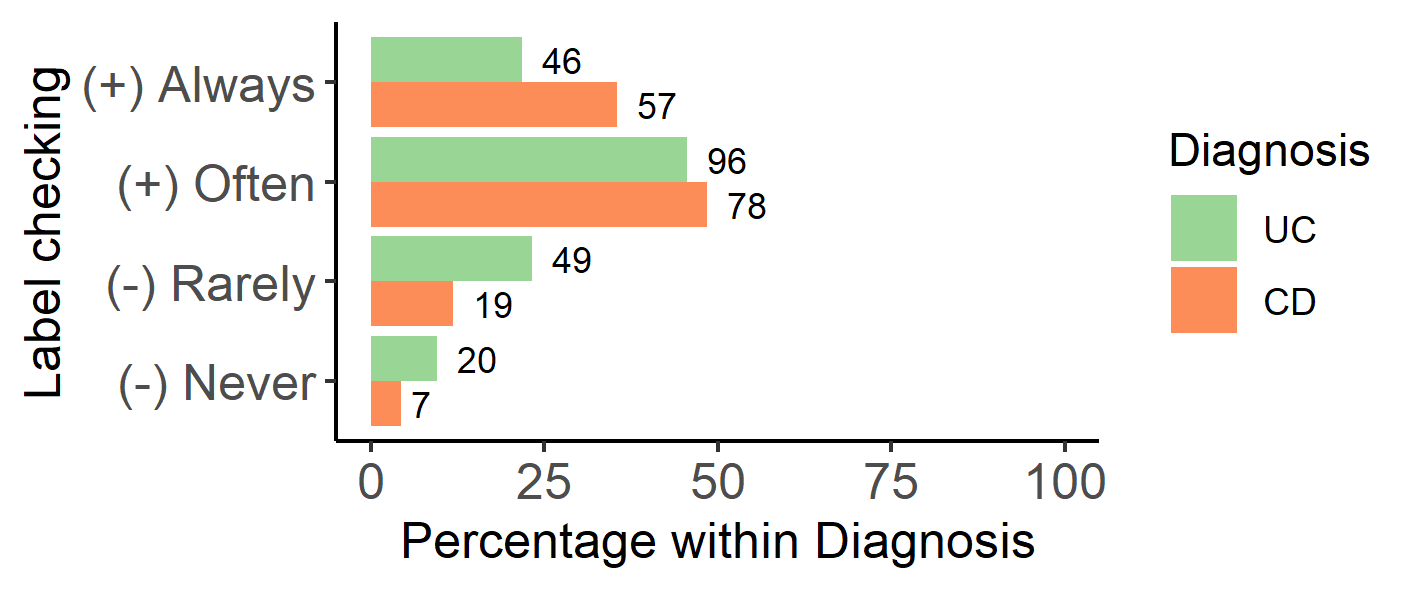


1. Are you able to manage the type and amount of food you eat depending on the situation? **(single-answer)**


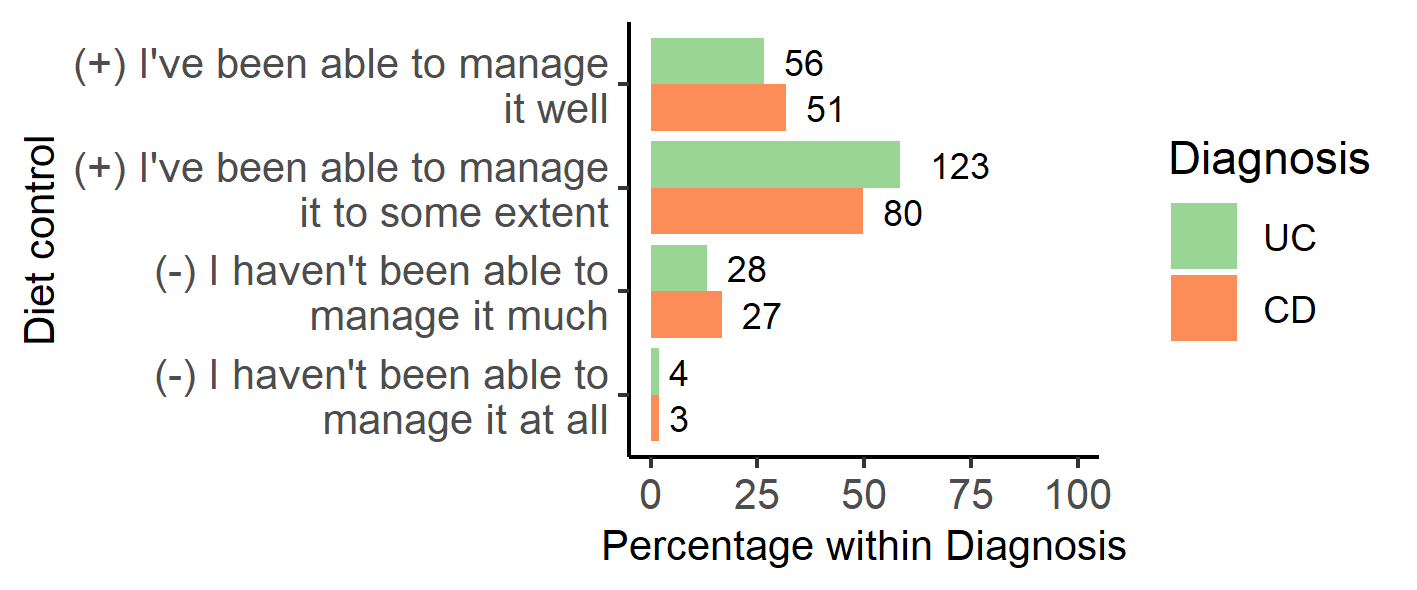


## Part II: Treatment

1. How much do you know about the treatment for UC or CD that you are receiving? **(single-answer)**


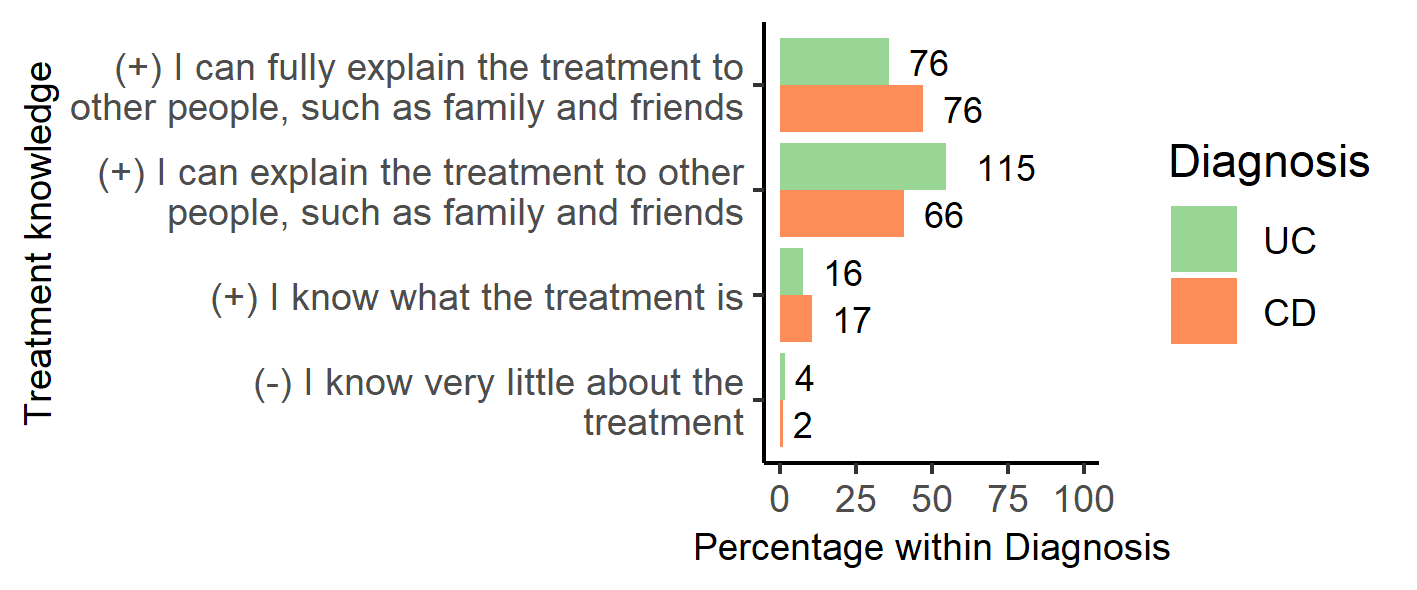


1. Do you understand what the test results mean for routine testing of UC or CD? **(single-answer)**


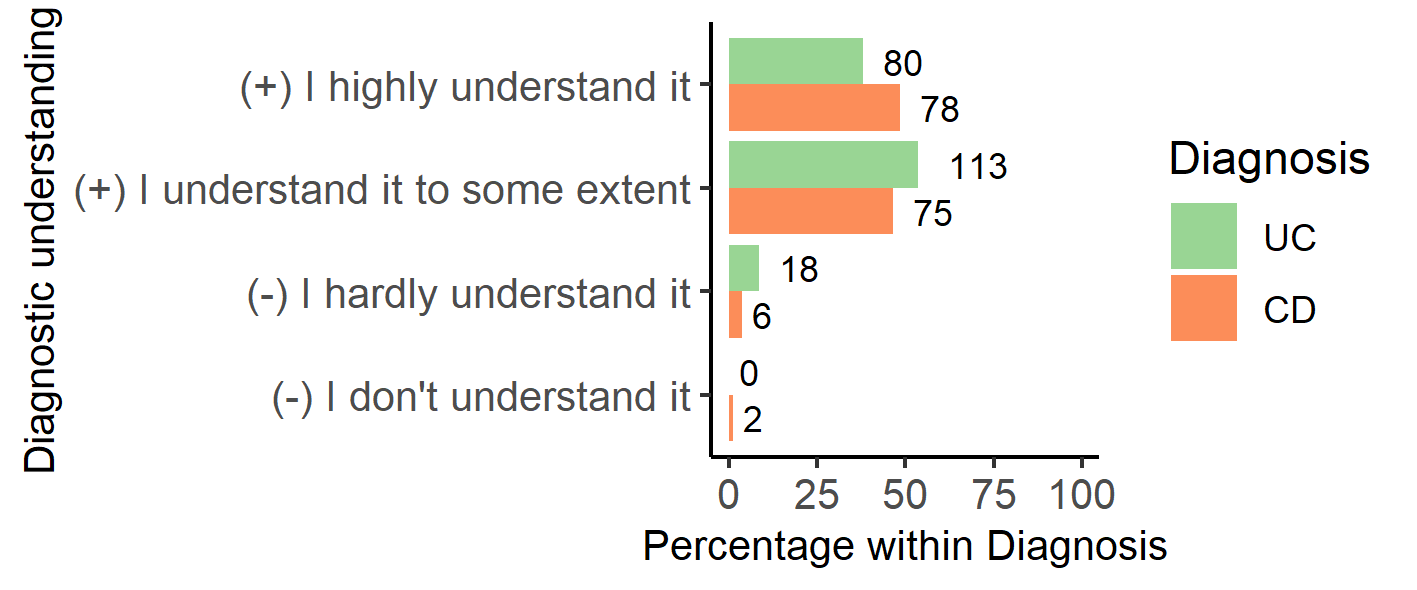


1. Do you know how to deal with forgetting to take medicine? **(single-answer)**


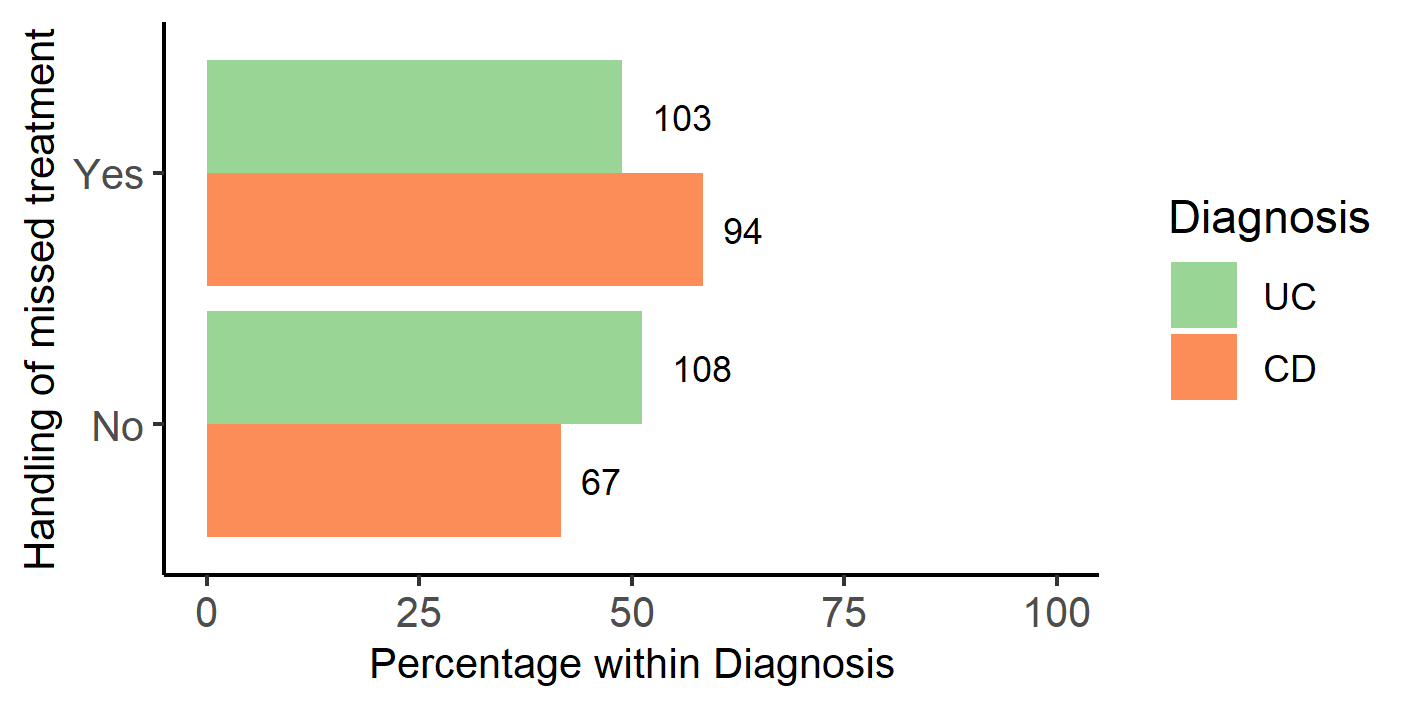


1. How satisfied are you with your current treatment? **(single-answer)**


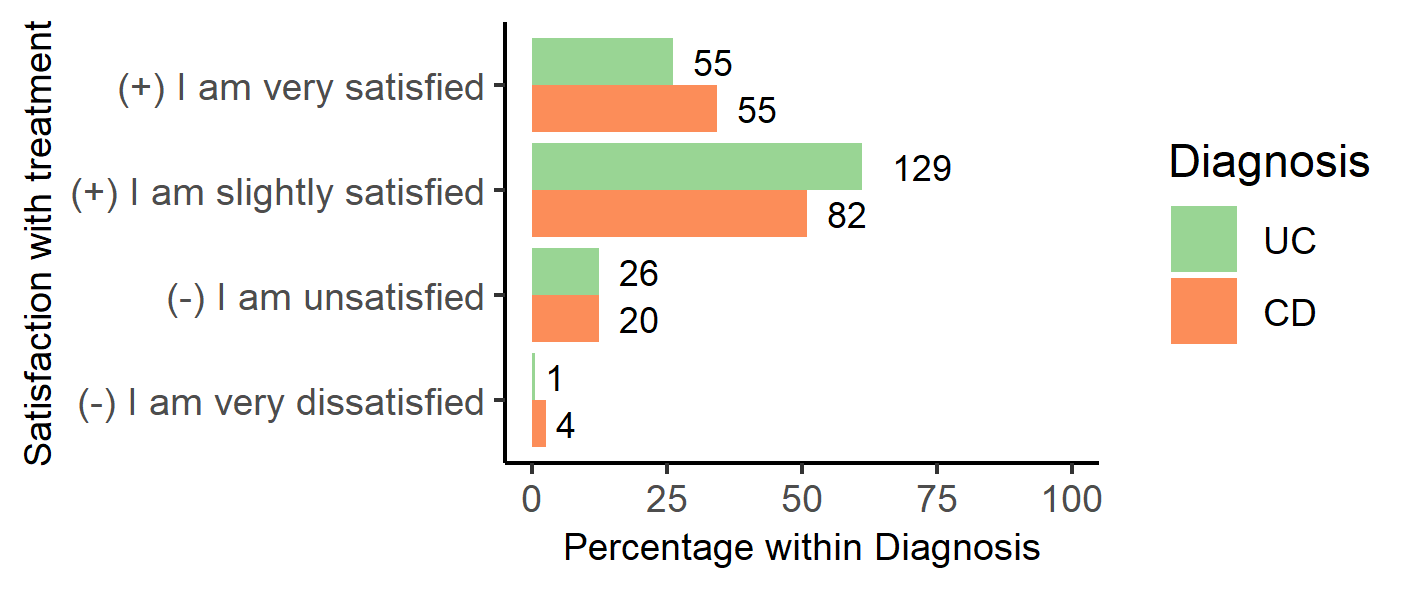


1. Please tell us why you have chosen the answer to the previous question. **(free-answer)**

(The datasets supporting this analysis are available from the corresponding author on reasonable request)

1. Who have you consulted about the treatment of UC or CD? Please select all the people you have consulted with. **(multi-answer)**


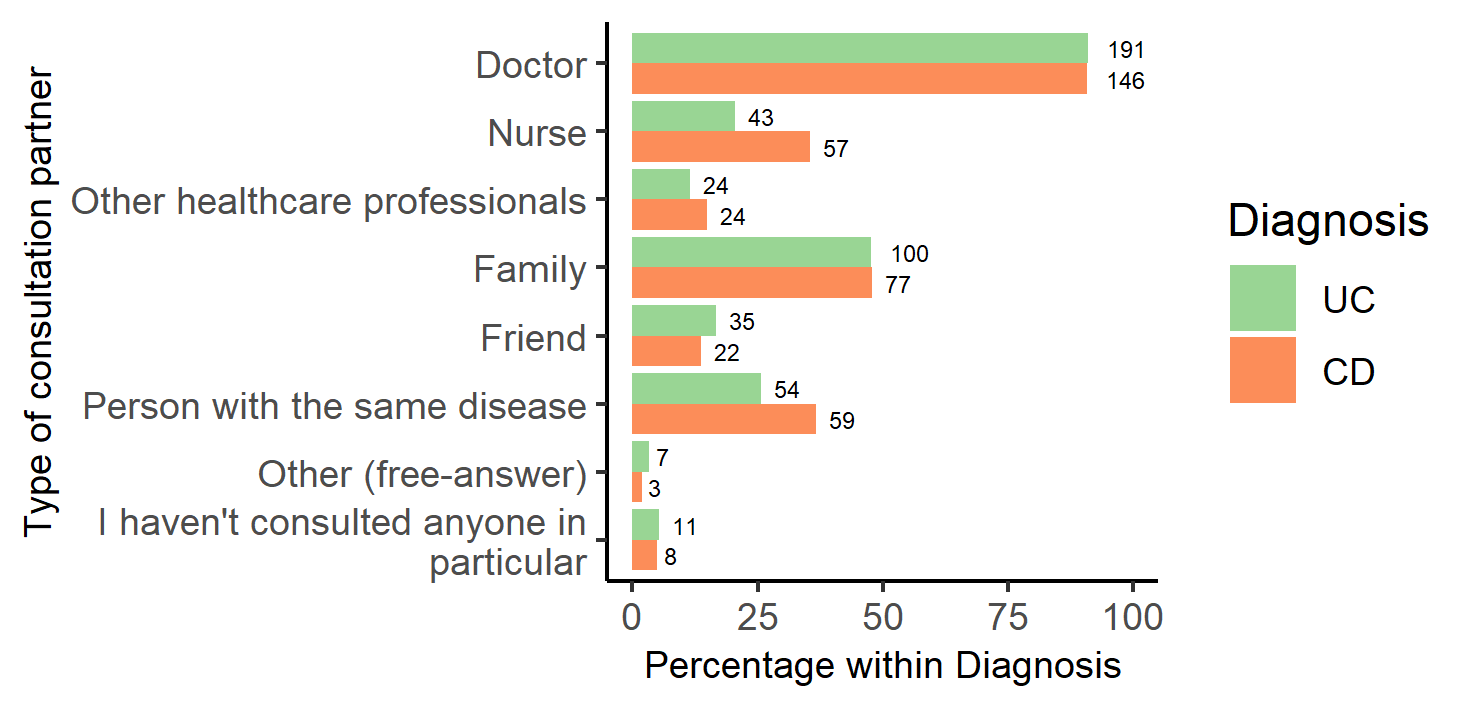


1. Is there anyone who can help you when you feel sick? **(multi-answer)**


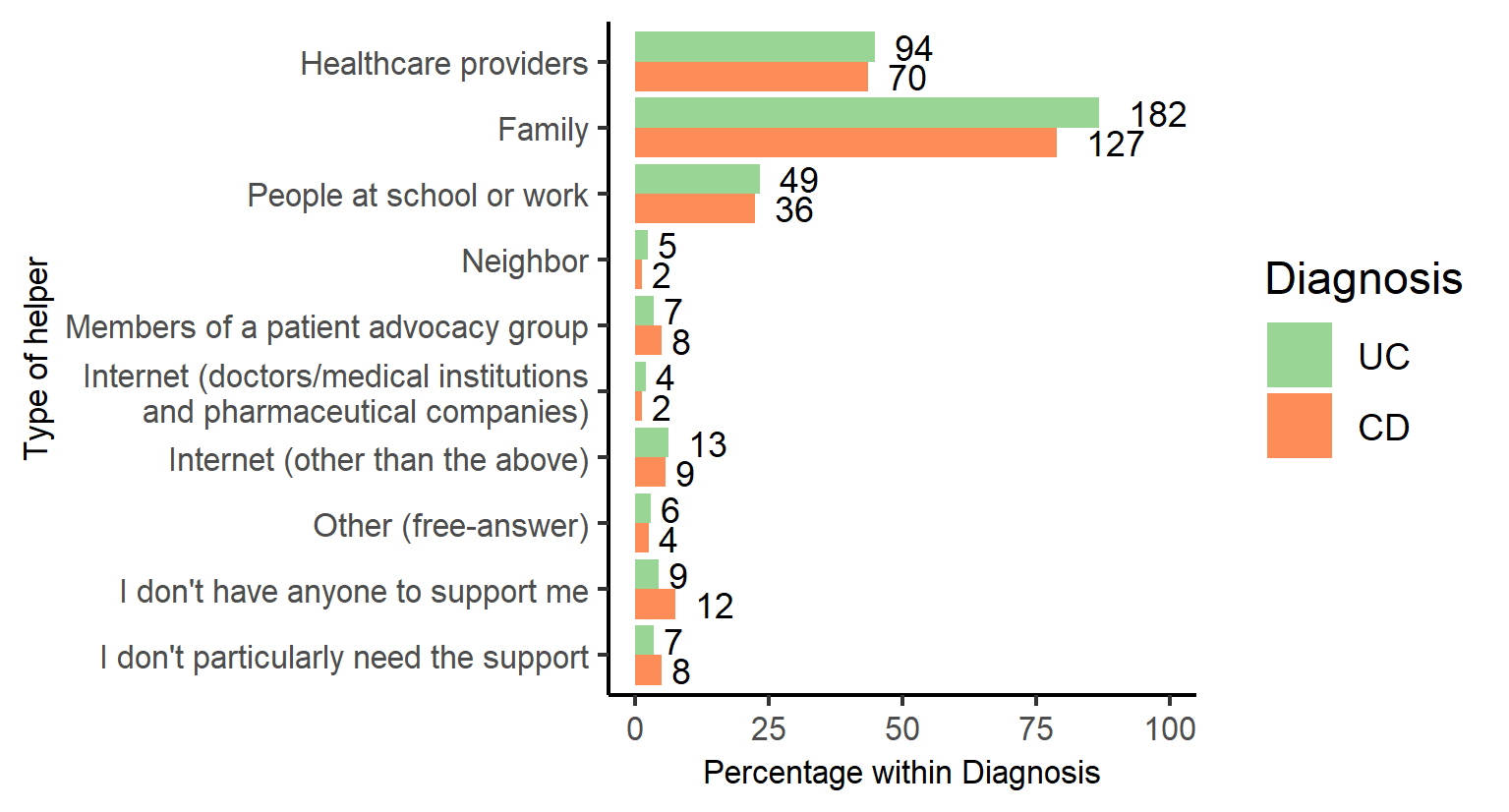


1. It is easy to consult with your doctor, nurse, or other healthcare professional who is involved in the treatment of UC or CD. **(single-answer)**


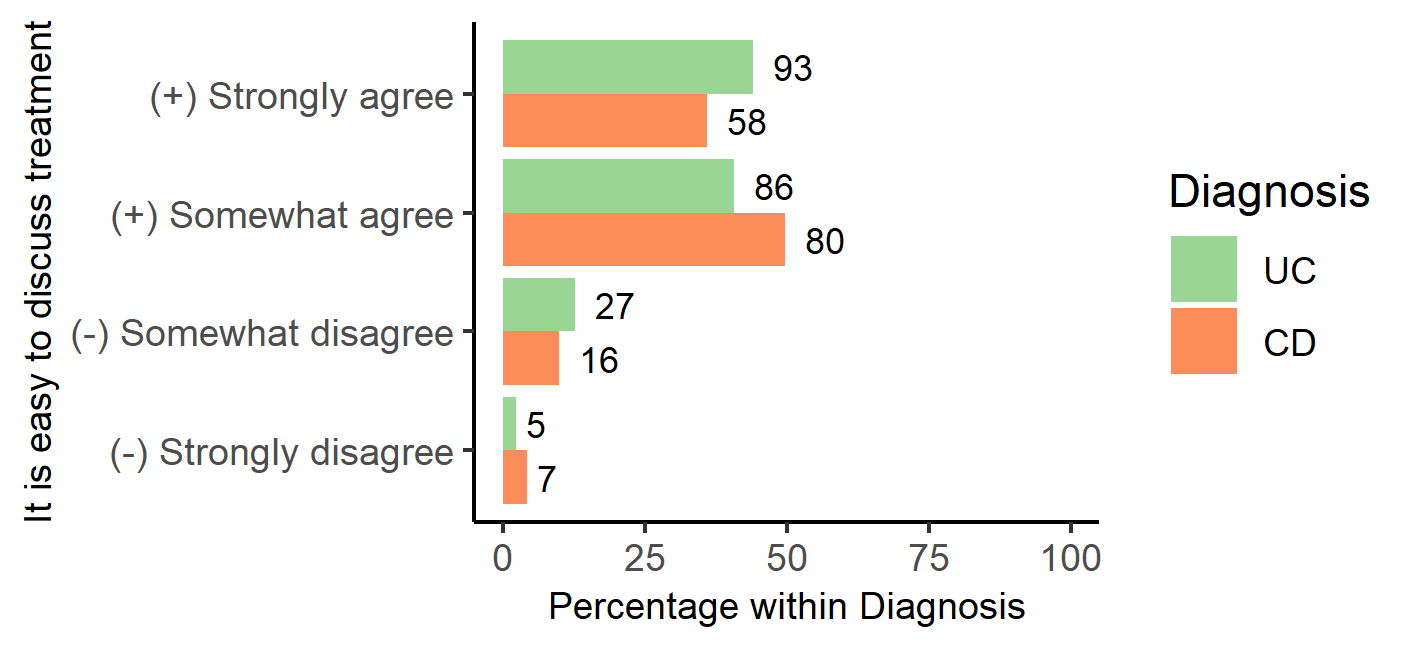


1. How long have you been able to continue the treatment for UC or CD that you have decided with your doctor? **(single-answer)**


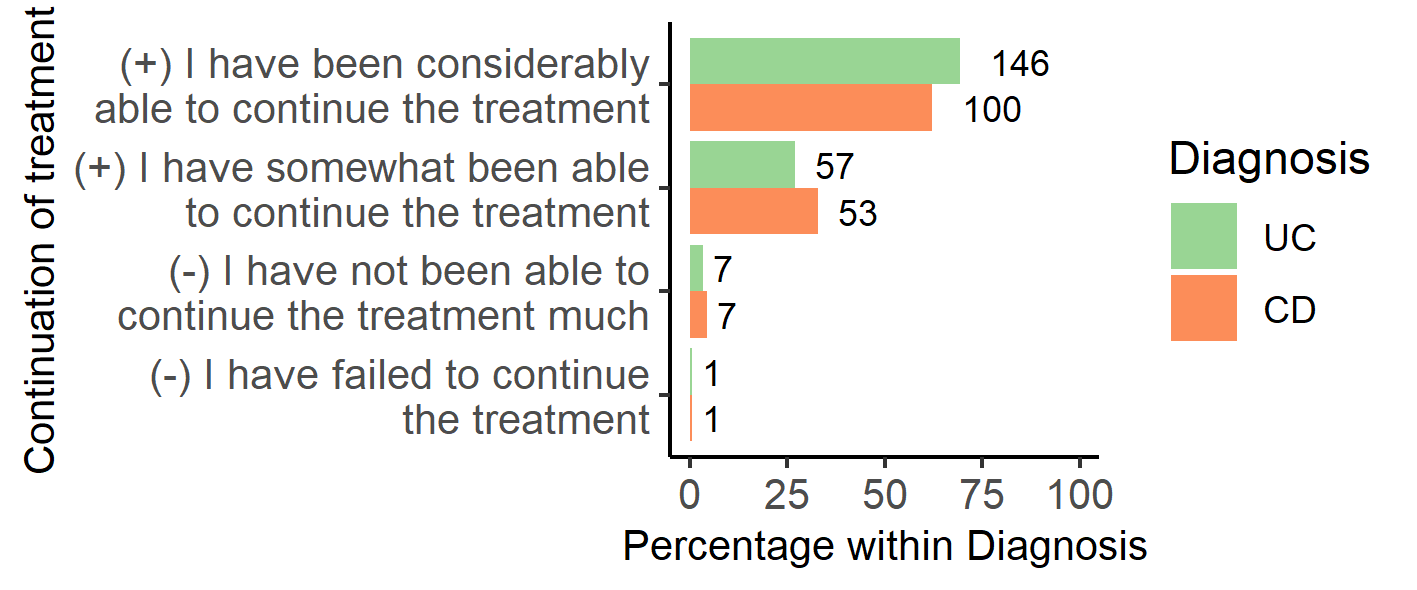


## Part III: Physical conditions and symptoms

1. Do you want to know more about UC or CD and its treatment? **(single-answer)**


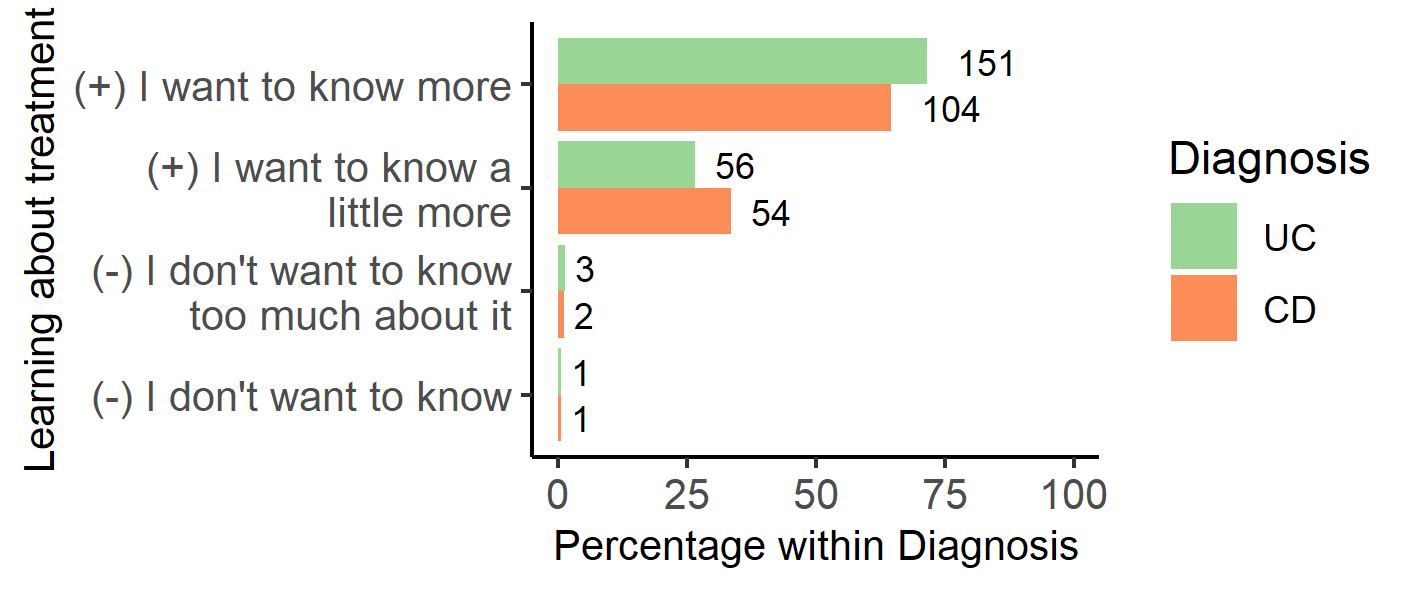


1. Who do you talk to about lifestyle (diet, exercise, sleep, etc.)? **(multi-answer)**


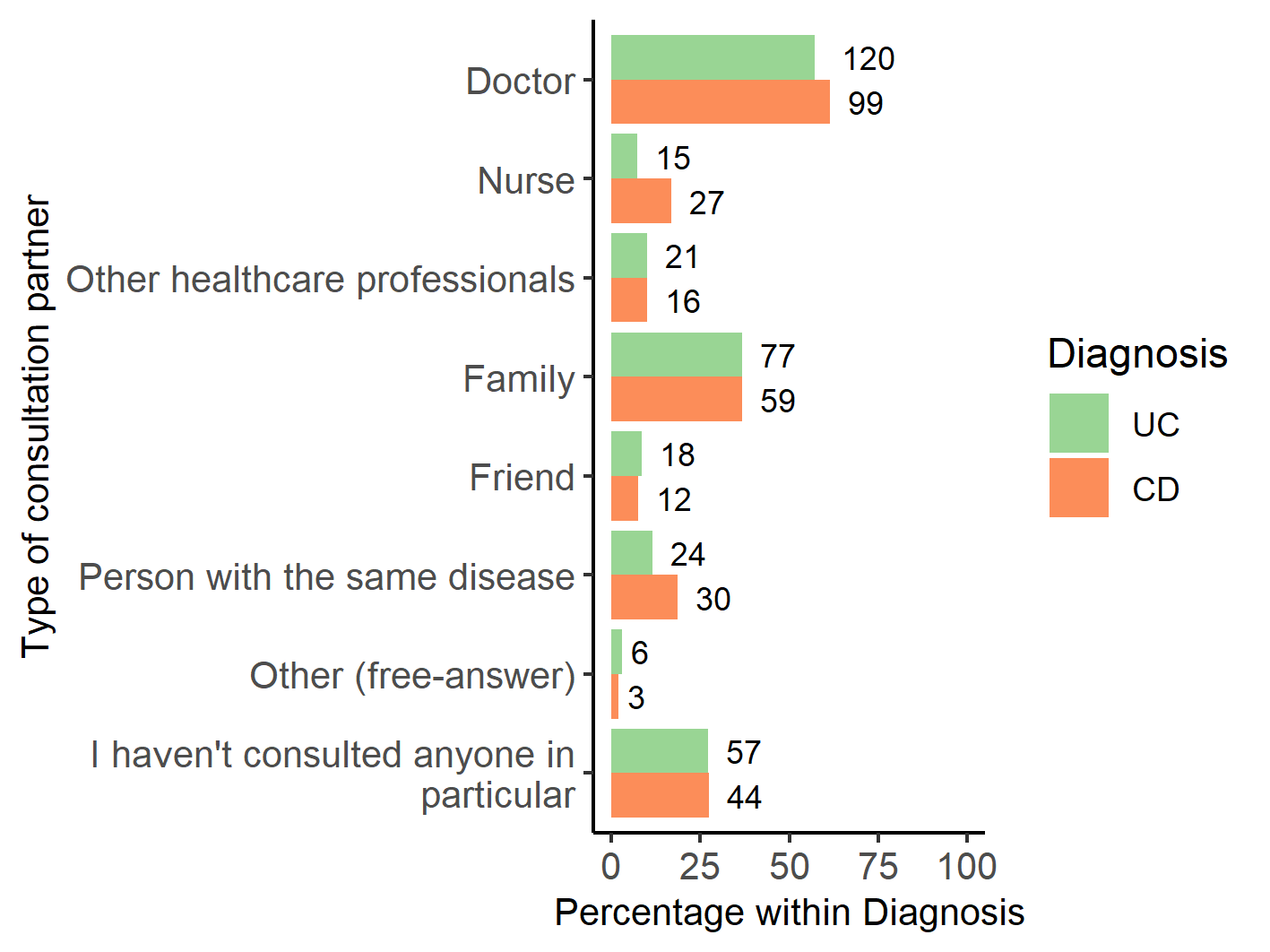


1. Who do you talk to about your physical condition and symptom management? Please select all the people you have consulted with. **(multi-answer)**


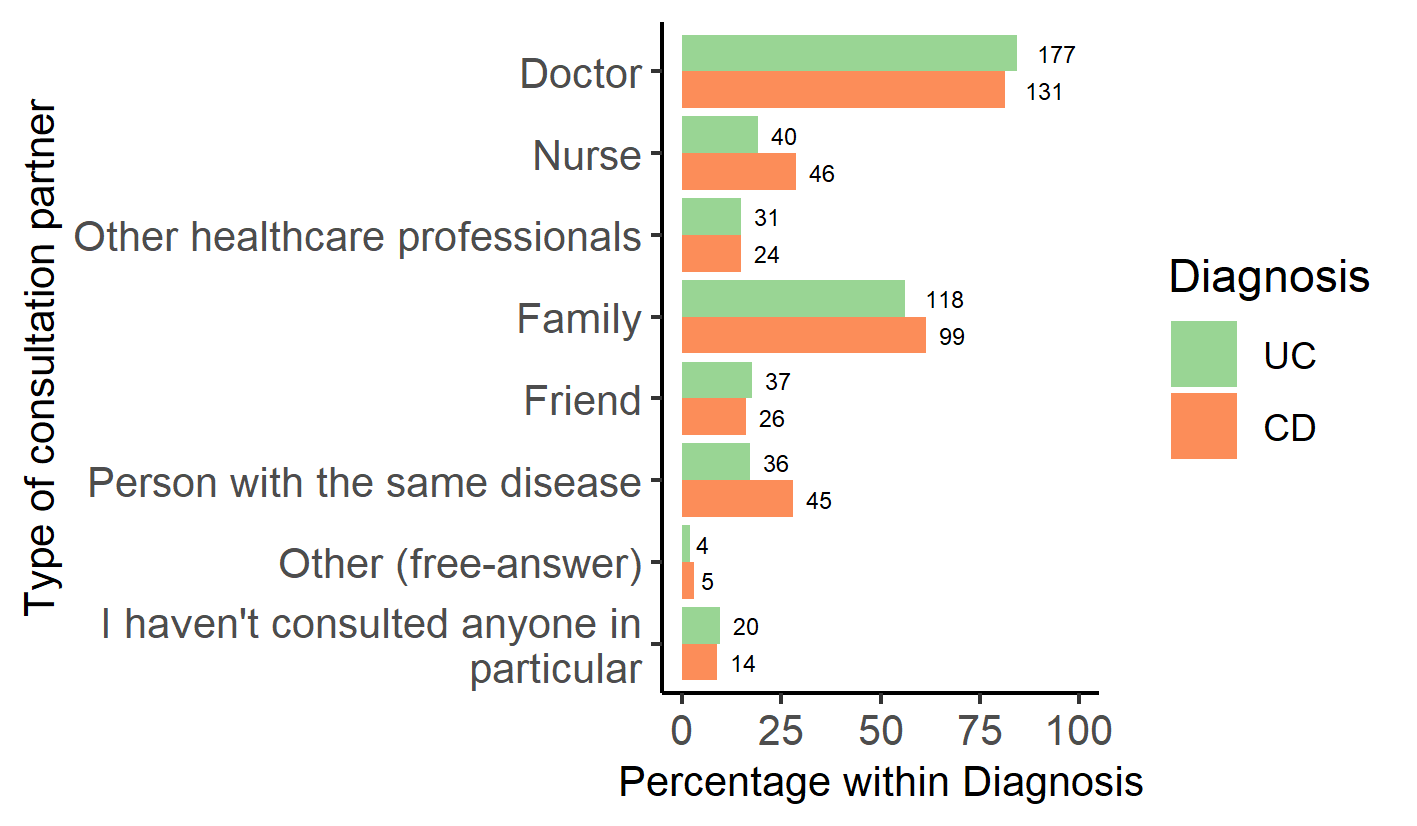


1. Have you recorded symptoms (stool frequency, condition, melena, abdominal pain, malaise, etc.) related to your UC or CD? **(single-answer)**


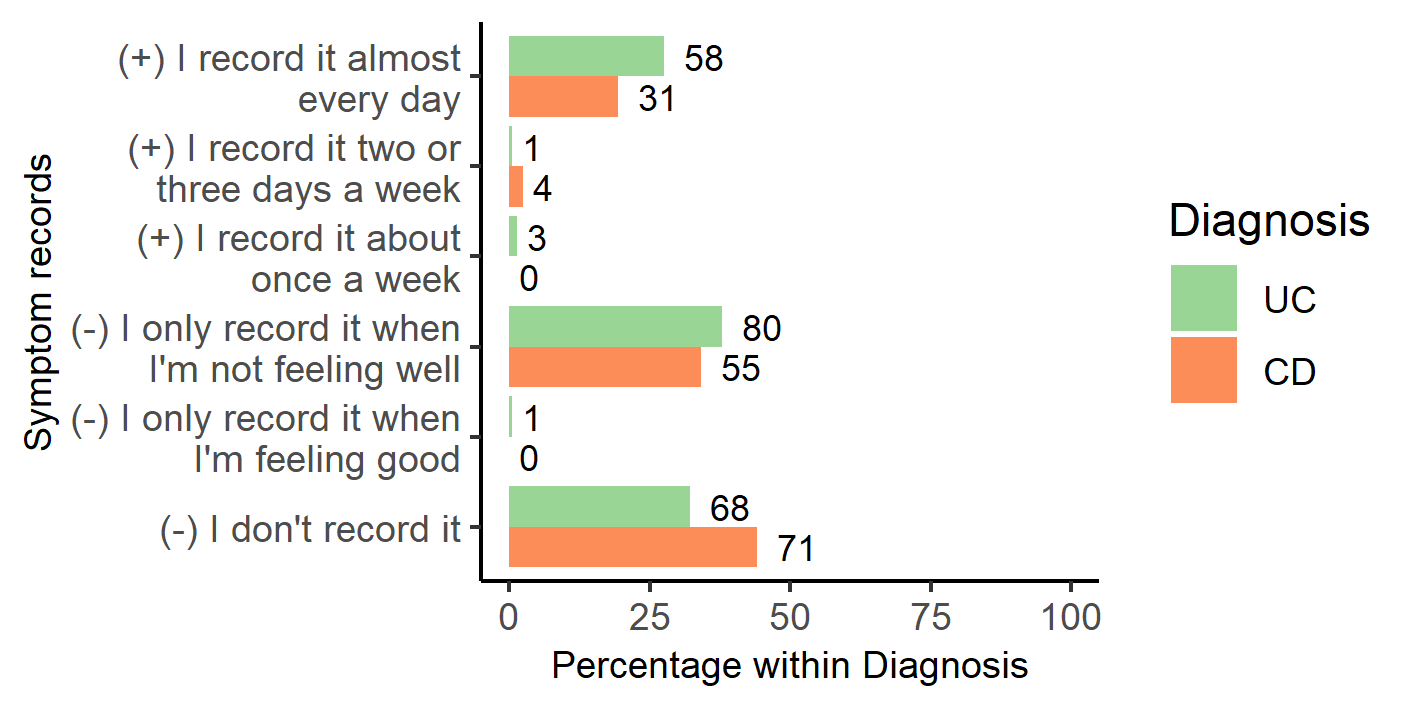


1. Do you find it helpful to understand your symptoms (stool frequency and condition, melena, abdominal pain, malaise, etc.) related to UC or CD in your daily life? **(single-answer)**


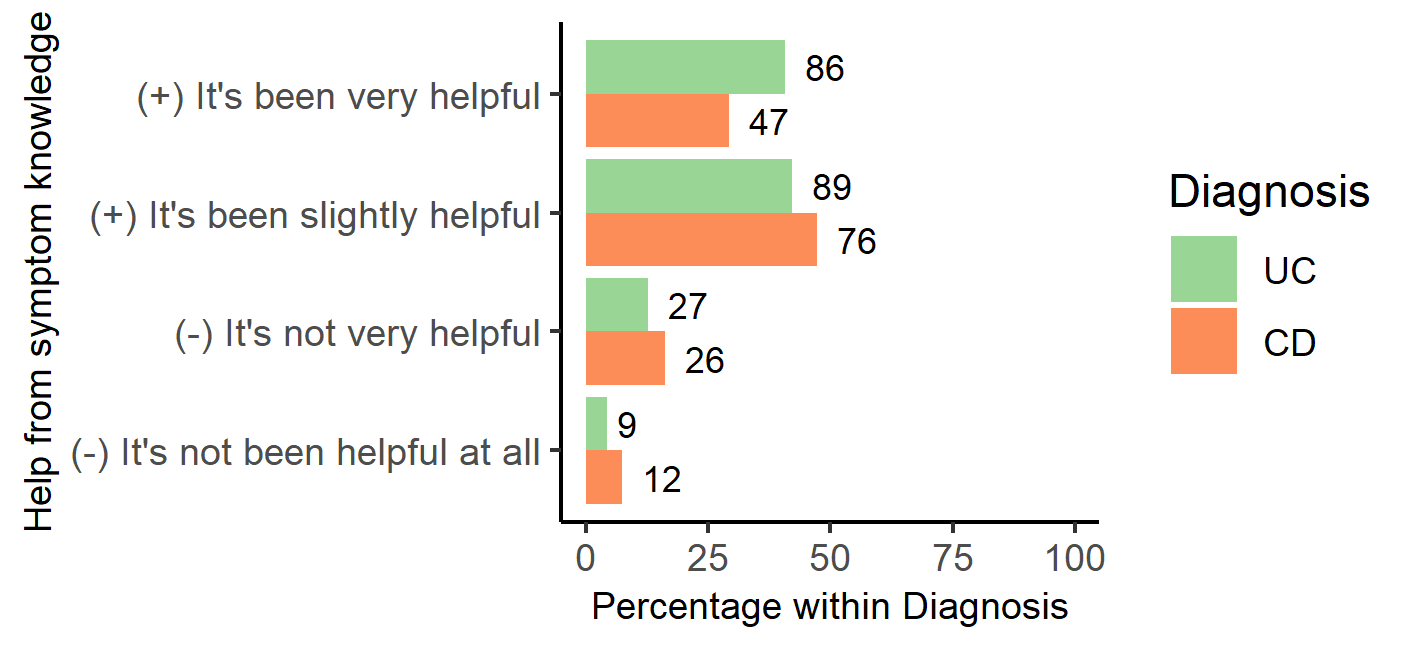


1. Are you able to change your diet, take leave or rest, or visit a doctor depending on your physical condition? **(single-answer)**


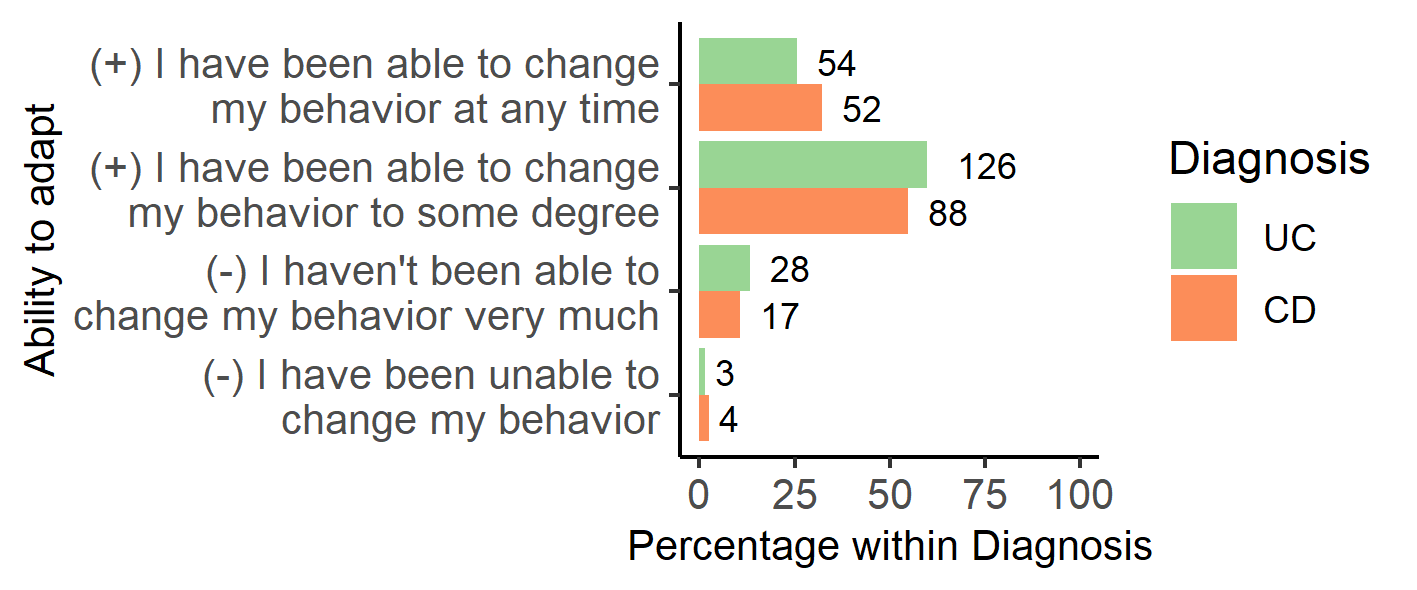


1. How confident are you in managing your physical condition/symptoms? **(single-answer)**


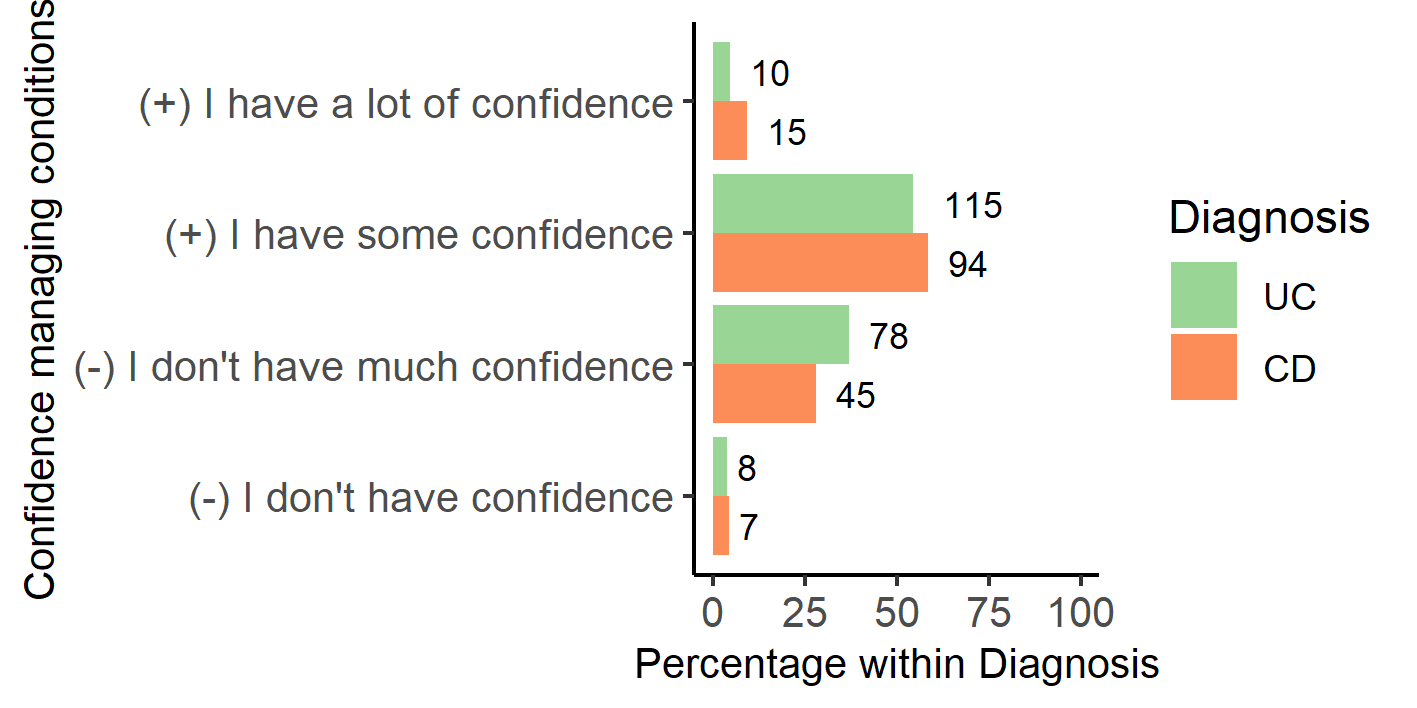


1. Knowing your symptoms related to UC or CD (e.g., stool frequency, abdominal pain, malaise, etc.) will help you achieve the life you want. **(single-answer)**


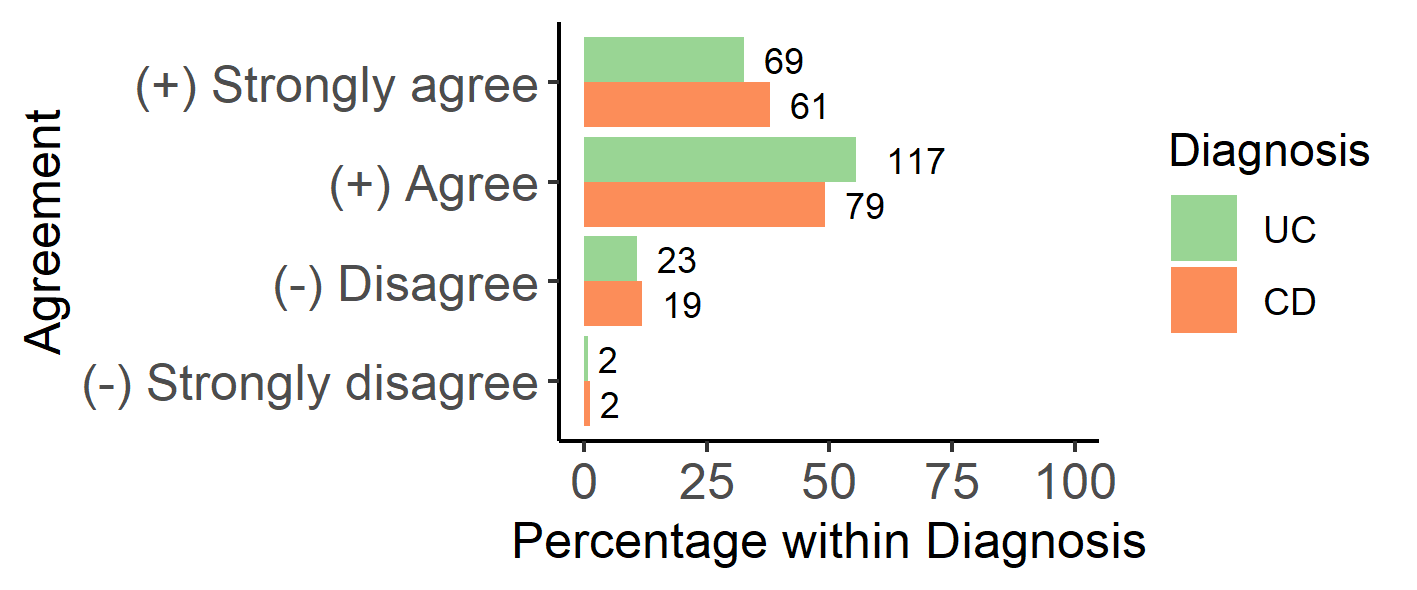


## Part IV: Stress

1. Do you feel anxious about the future because of UC or CD? **(single-answer)**


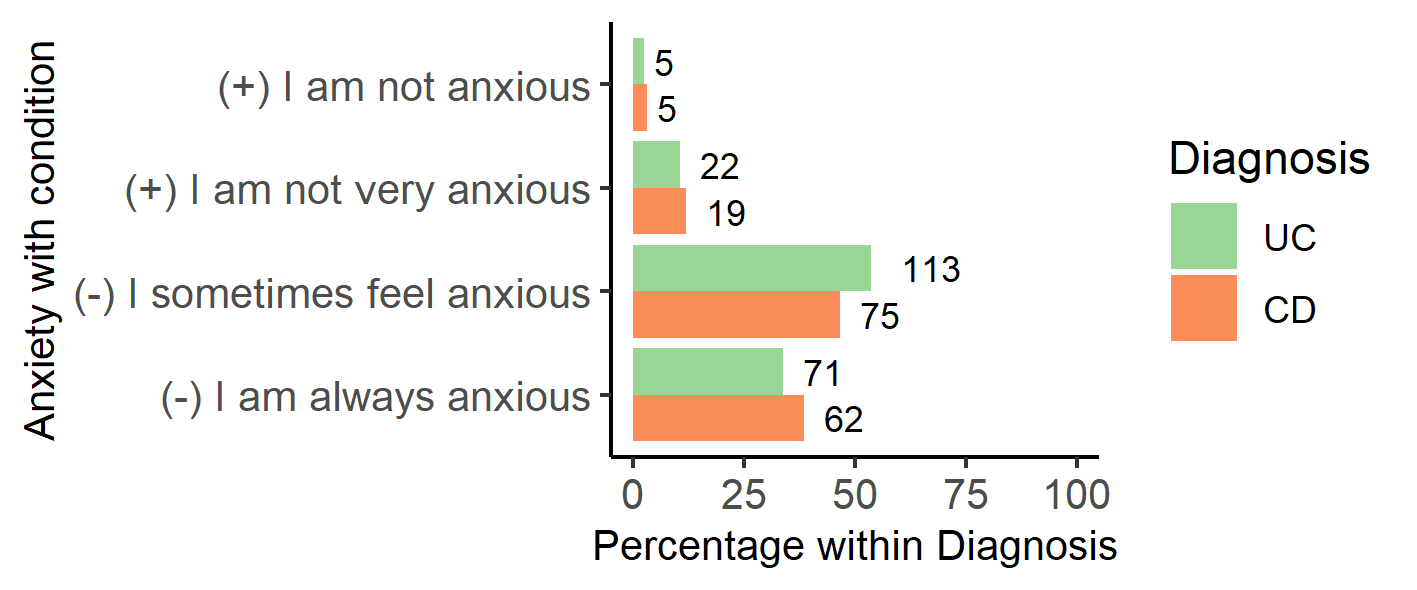


1. How much do you think you can control (divergence) stress when you feel it? **(single-answer)**


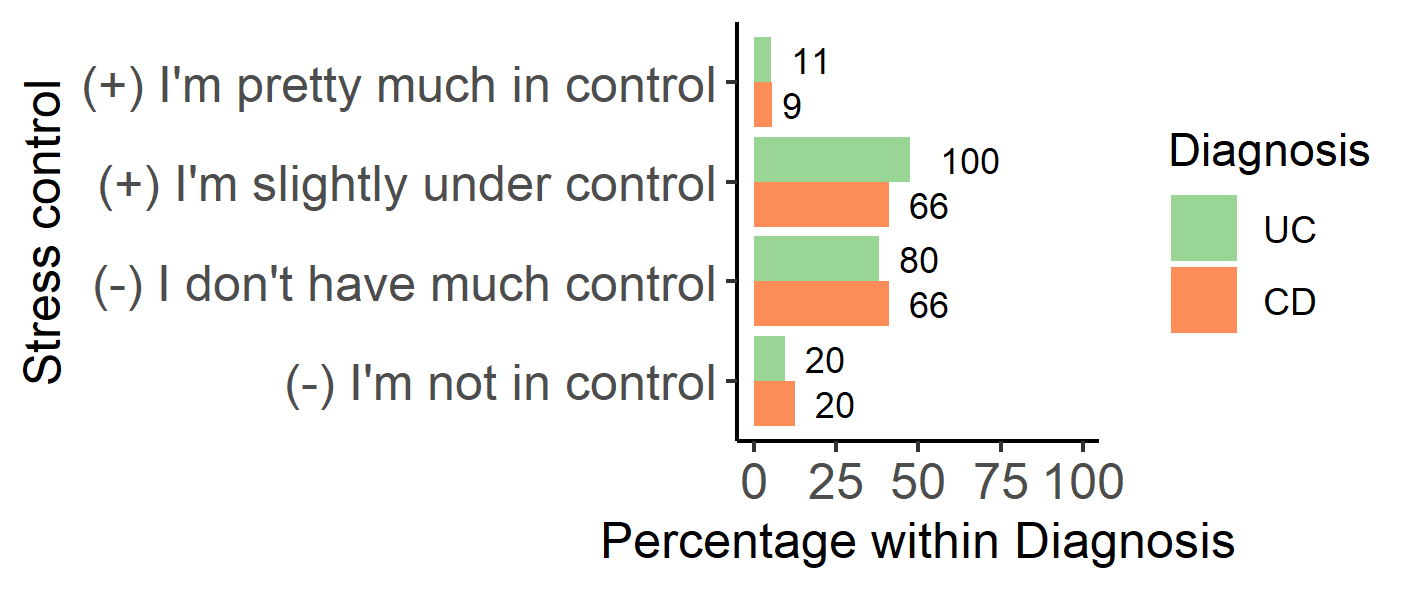


1. Do you think that controlling stress (de-stress) will help you achieve the life you want? **(single-answer)**


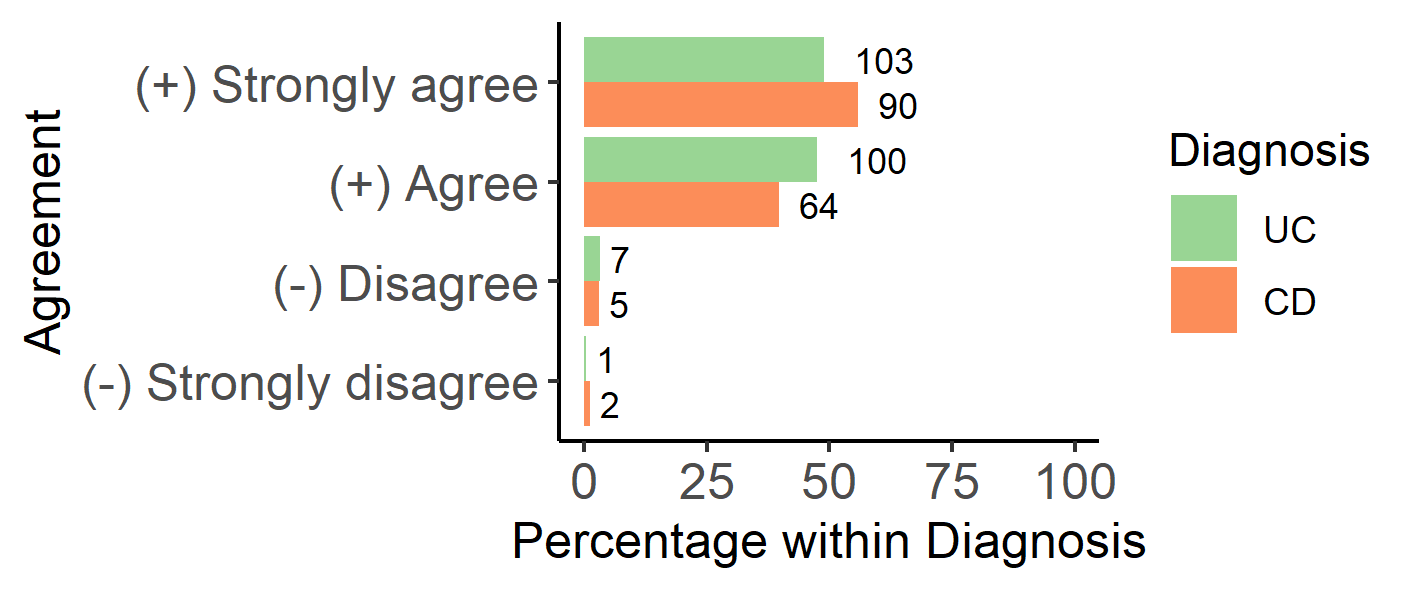


## Part V: Financial concerns

1. Do you know your monthly treatment costs (costs associated with UC or CD, including medical examination and drug costs)? **(single-answer)**


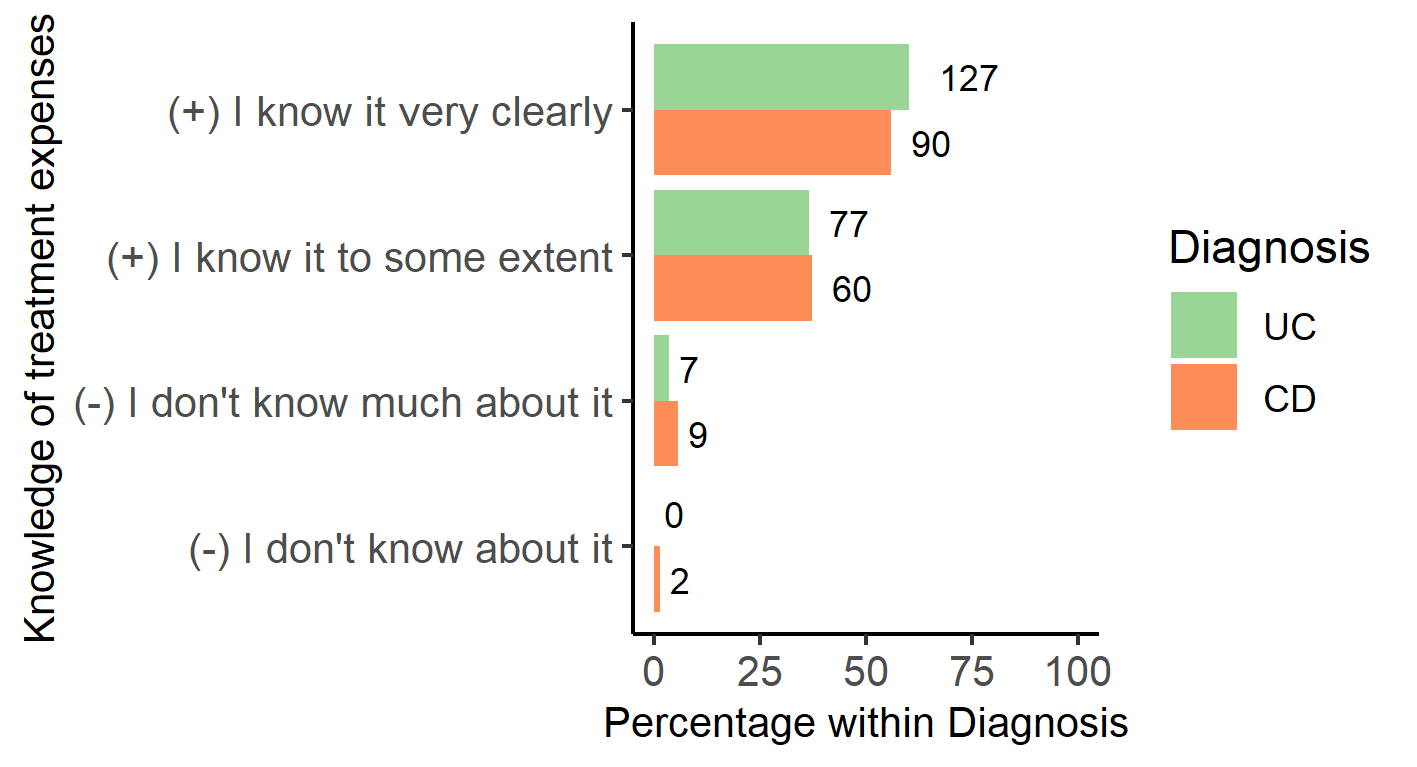


1. How many days out of work or school in the last year for reasons related to UC or CD? **(single-answer)**


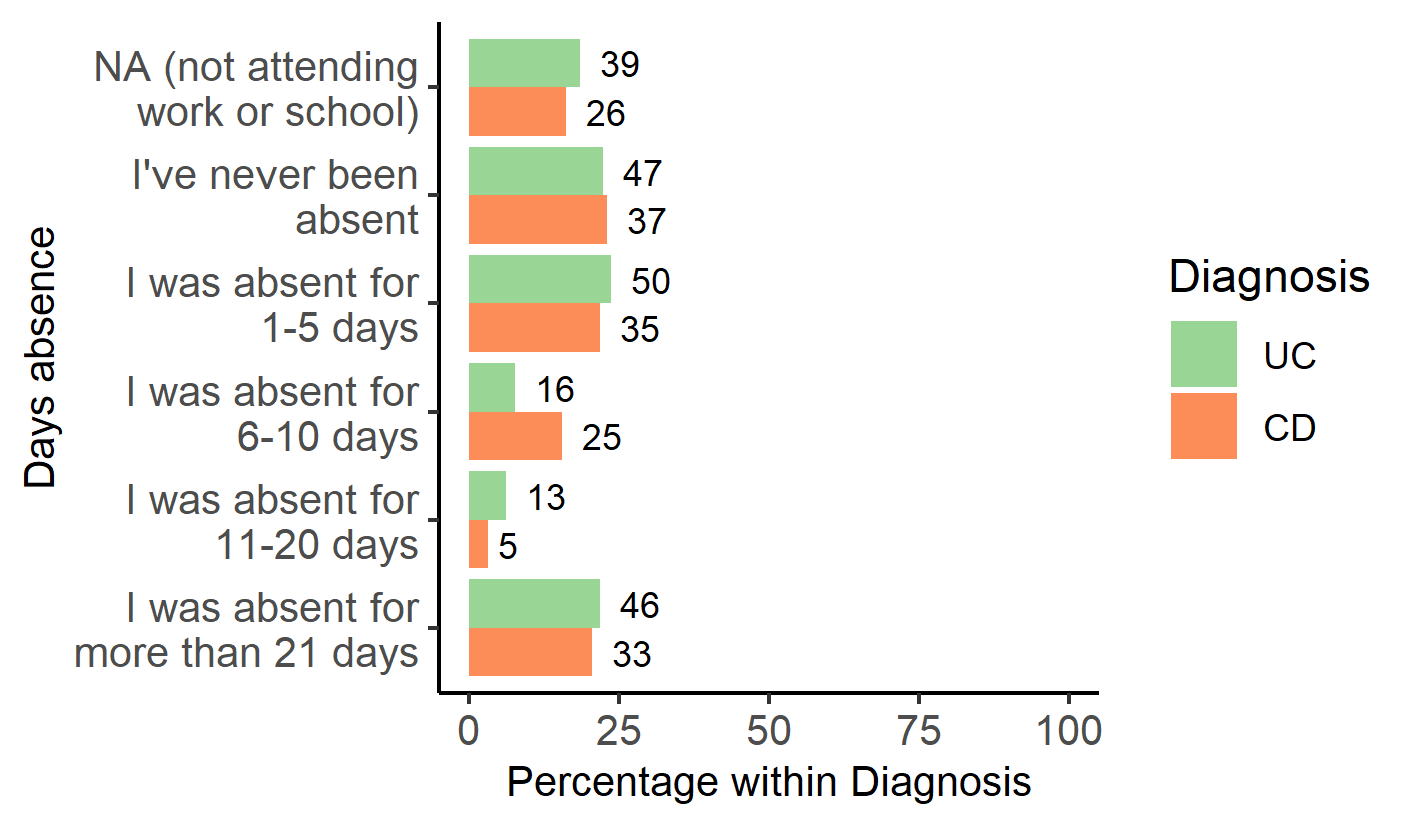


1. Who can you consult about employment? Please select all the people you have consulted with. **(multi-answer)**


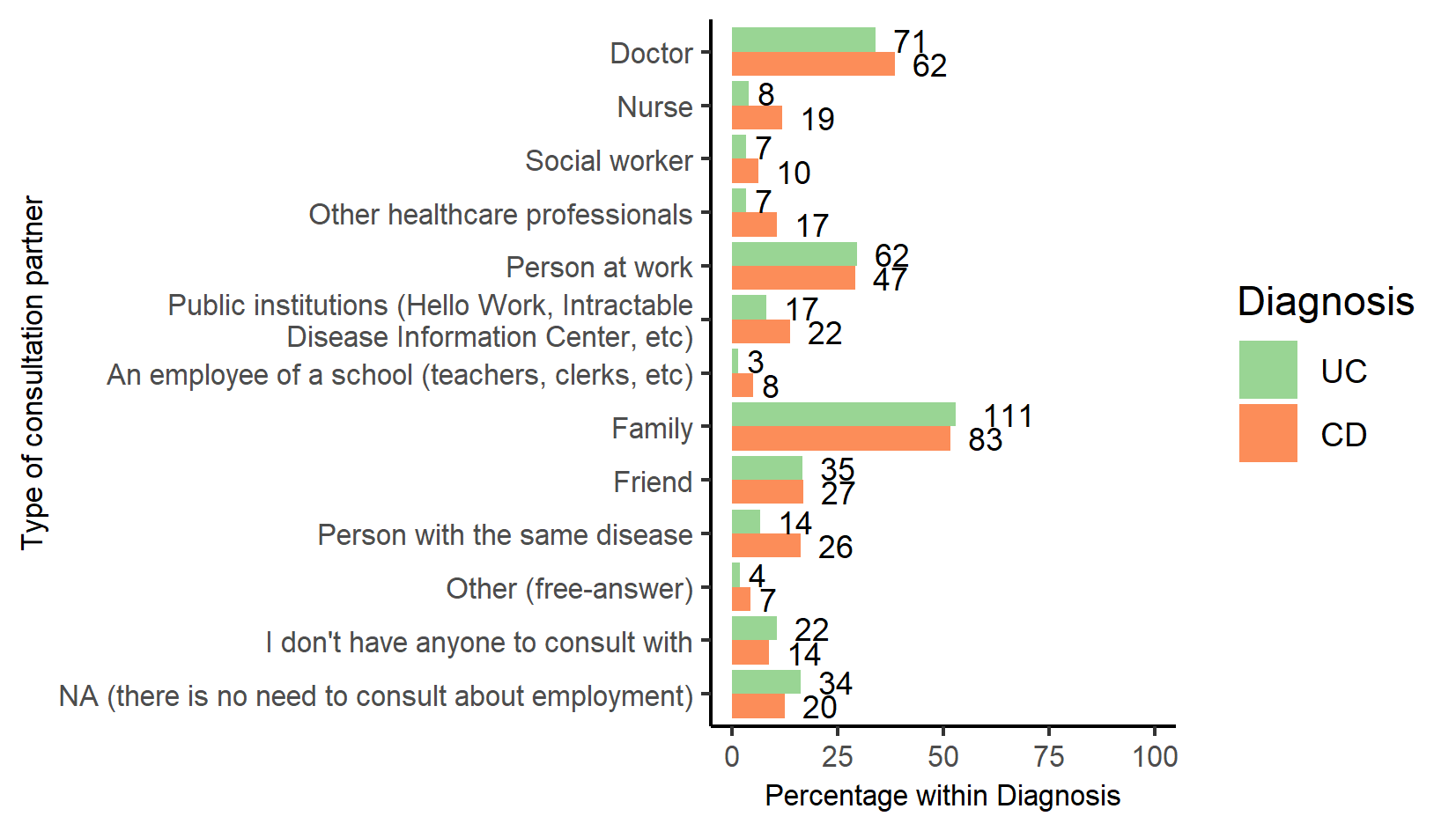


1. Any relief from the financial anxiety (work, money) in the treatment of UC or CD will help you achieve the life you want? **(single-answer)**


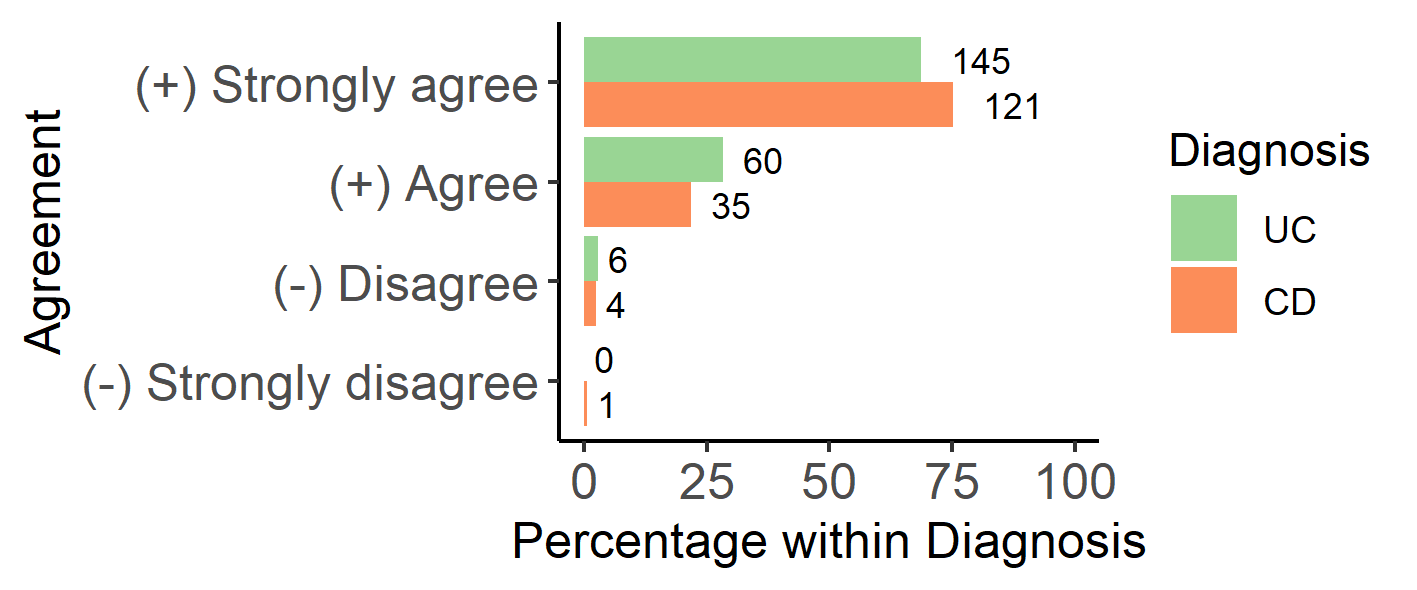


## Part VI: Support tools and services

1. Where do you get information about UC or CD? Please select all sources with experience. **(multi-answer)**


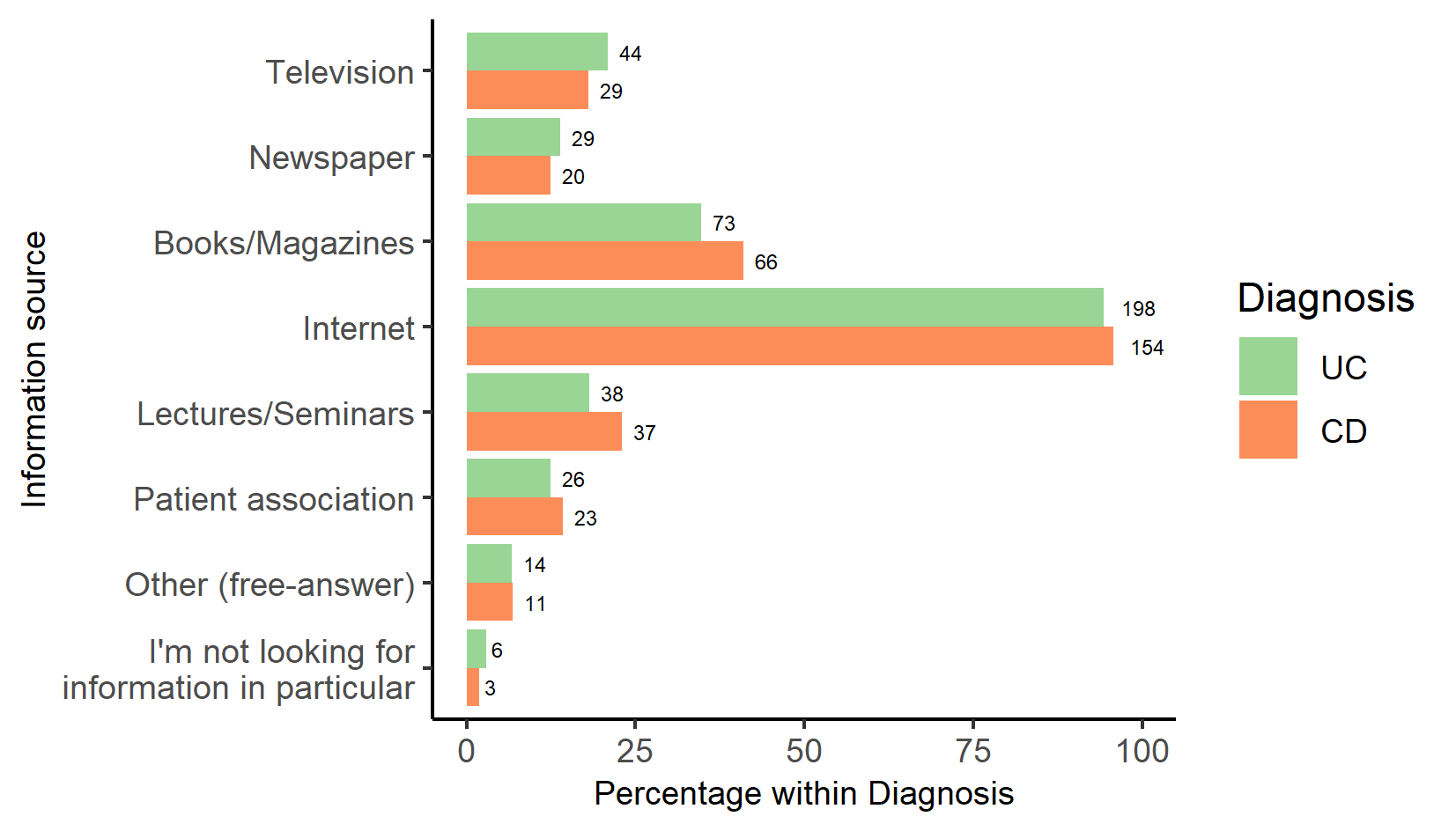


1. Who provides information about UC or CD? Select all of the people who have informed you. **(multi-answer)**


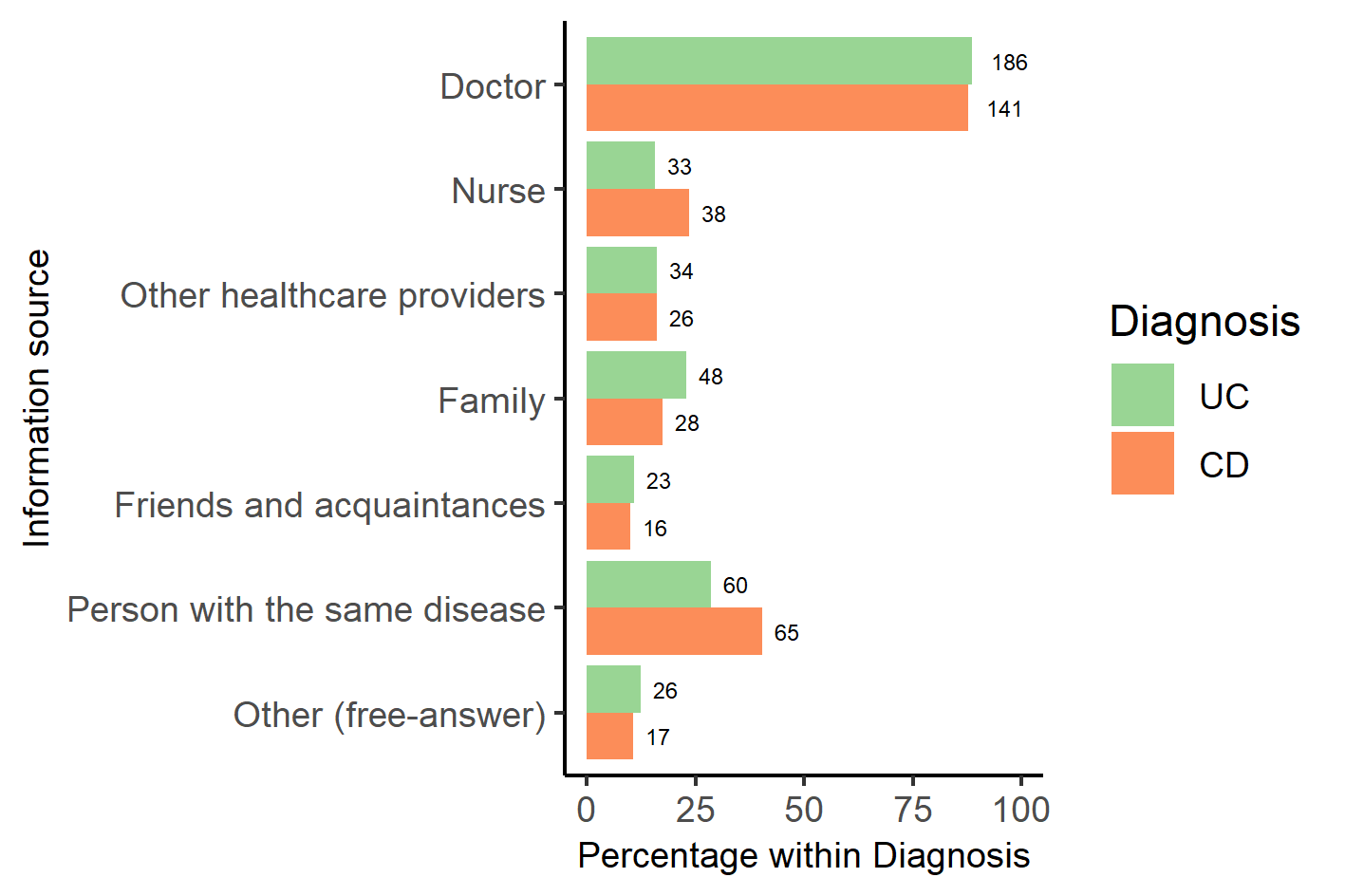


1. What kind of support do you want to get for UC or CD? Please select all the support you would like to receive. **(multi-answer)**


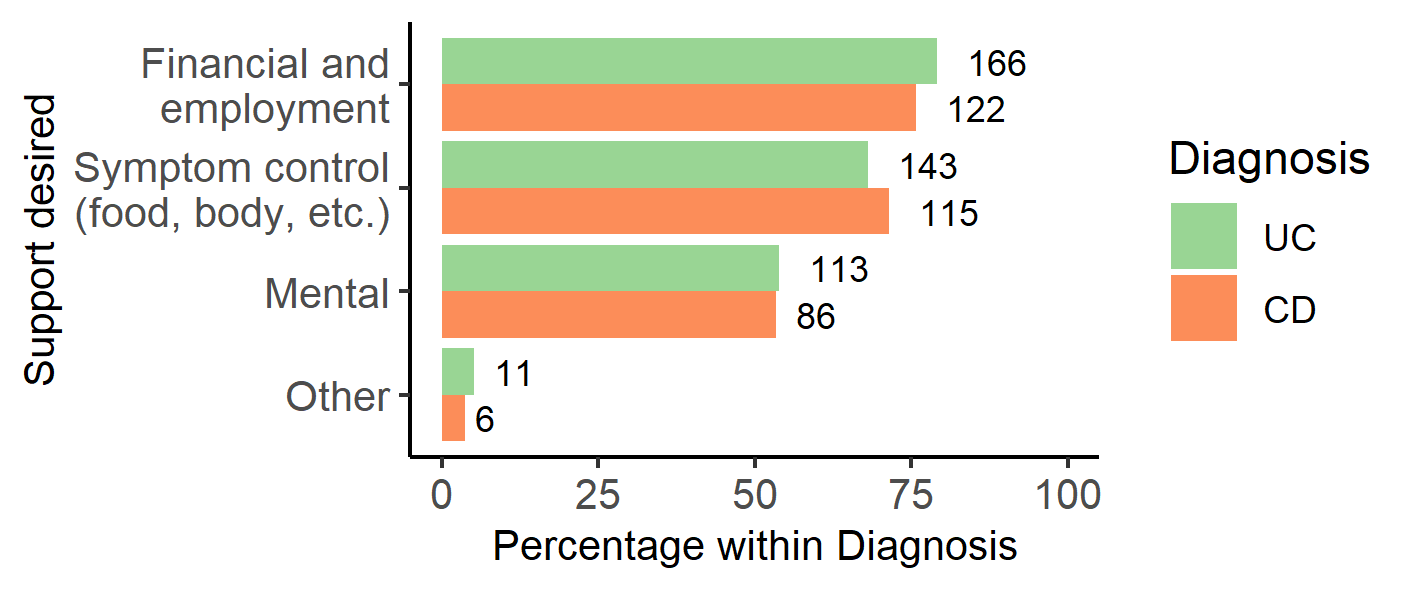


1. Please let us know what kind of support you have in mind for the answer you selected in the previous question. There is no character limit. **(free-answer).**

(See Supplementary Methods and Results 2.1 for coded answers, or available from the corresponding author on reasonable request.)

1. What tools or services (apps, helplines, etc.) are you actually using for self-management of UC or CD? There is no character limit. **(free-answer)**

(The datasets supporting this analysis are available from the corresponding author on reasonable request.)

## Part VII: Perception of daily life

1. How do you perceive your daily life with UC or CD? Please select the closest one. **(single-answer)**


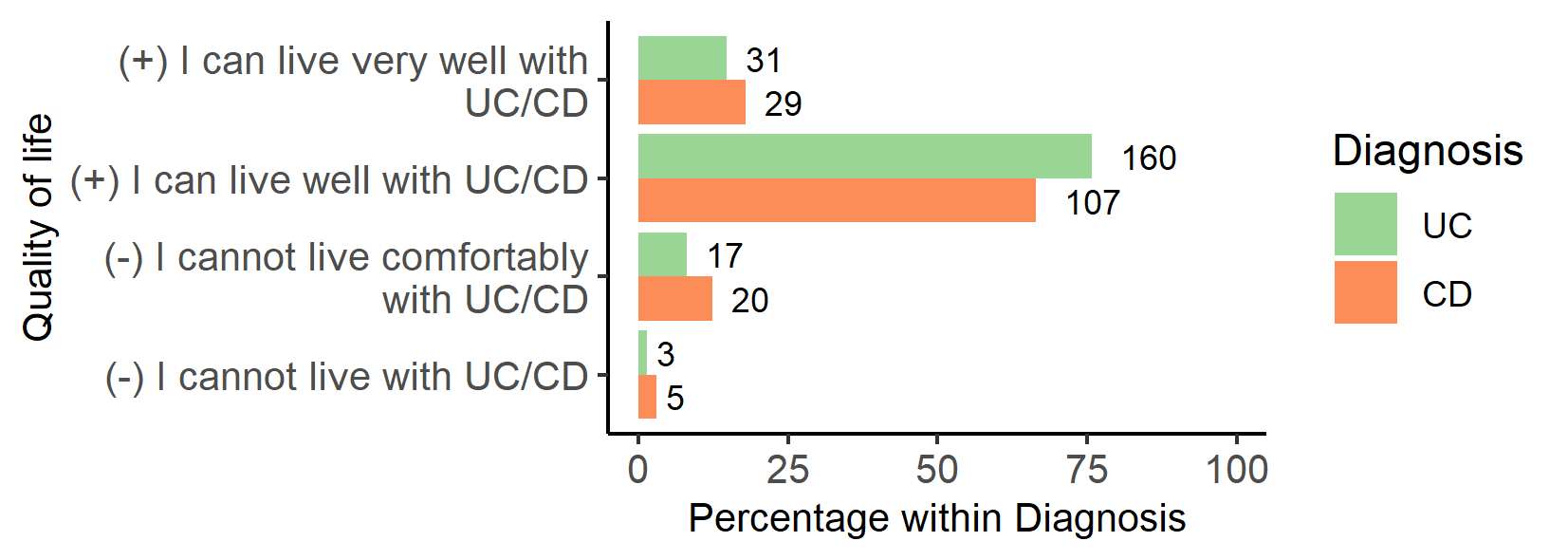


*5-ASA: mesalamine. Bio/JAKi: biologics or Janus kinase inhibitors. CD: Crohn’s disease. JAK: Janus kinase. NA: not applicable. UC: ulcerative colitis.*
